# Targeting ubiquitination in disease and therapy

**DOI:** 10.1038/s41392-025-02392-8

**Published:** 2025-12-23

**Authors:** Xiaojuan Yang, Tian Lan, Buzhe Zhang, Xue Tao, Weili Qi, Kunlin Xie, Yunshi Cai, Chang Liu, Junhong Han, Hong Wu

**Affiliations:** 1 Laboratory of Hepatic AI Translation, Frontiers Science Center for Disease-Related Molecular Network, West China Hospital, Sichuan University, Chengdu, 610041 China; 2 Department of Biotherapy, Cancer Center and State Laboratory of Biotherapy, and Frontiers Science Center for Disease-related Molecular Network, West China Hospital, Sichuan University, Chengdu, 610041 China; 3 Liver Transplant Center, Transplant Center, West China Hospital, Sichuan University, Chengdu, 610041 China; 4 Division of Abdominal Tumor Multimodality Treatment, Cancer Center, West China Hospital, Sichuan University, No. 37 Guoxue Alley, Chengdu, 610041 Sichuan China; 5 Intervention Center, West China Hospital, Sichuan University, No. 37 Guoxue Alley, Chengdu, 610041 Sichuan China

**Keywords:** Biophysics, Diseases

## Abstract

Ubiquitination, a critical posttranslational modification (PTM), involves the enzymatic covalent attachment of ubiquitin to target proteins. This process is fundamental for maintaining cellular homeostasis and regulating key biological functions. The ubiquitination pathway, orchestrated by ubiquitin and its associated enzymes, offers remarkable versatility, acting as a cellular sentinel to ensure precise spatiotemporal control of essential molecular processes. Importantly, the components and mechanisms of ubiquitination can be finely tuned in various ways. Dysregulation of this system can disrupt normal biological processes and contribute to the development of various serious human diseases. These findings underscore the importance of investigating ubiquitination to understand disease mechanisms and develop effective treatment strategies. In this review, we summarize the historical developments and key milestones in ubiquitination research, with a focus on its roles in both health and disease. We explore the components and mechanisms involved, the relevant signaling pathways and their crosstalk, and the multilayered regulatory functions of ubiquitination under physiological and pathological conditions. The pathological contexts discussed include cancer, neurodegenerative disorders, cardiovascular diseases, inflammatory conditions, autoinflammatory disorders and developmental disorders. Enhancing our understanding of ubiquitination could provide novel insights into disease pathogenesis and identify new therapeutic targets. We also highlight emerging strategies for cancer treatment, such as proteolysis-targeting chimeras (PROTACs) and molecular glues. Furthermore, we review therapeutic targets and recent progress in clinical research, including ongoing clinical trials and FDA-approved drugs, aimed at leveraging the ubiquitination pathway for disease treatment.

## Introduction

**Fig. 1:**
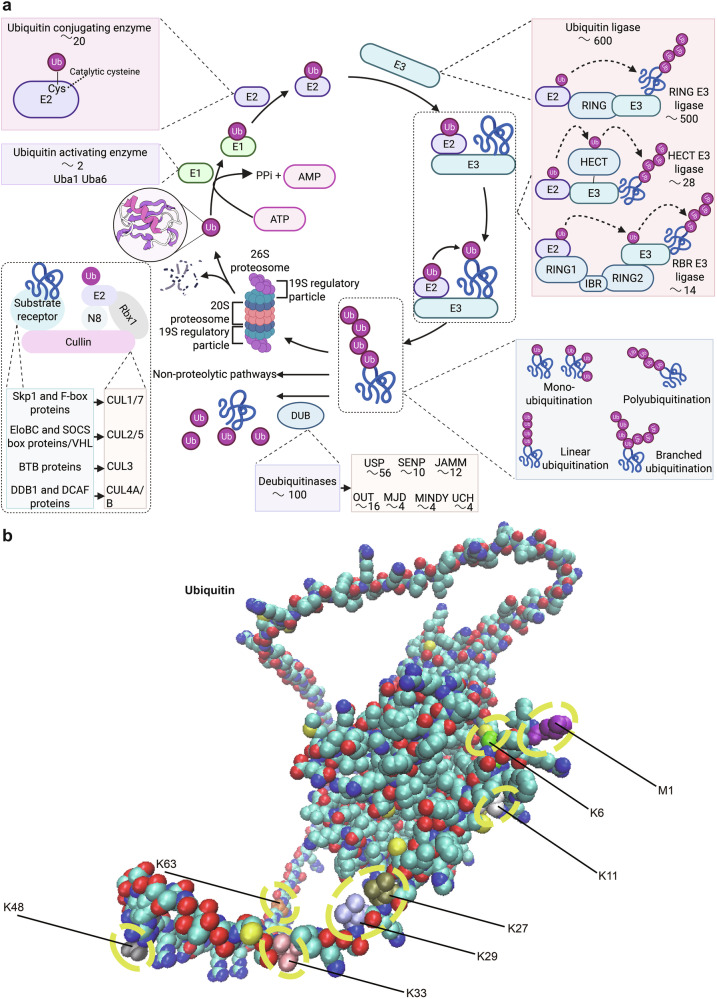
The components and processes of the UPS (**a**), as well as the specific locations of ubiquitin chain types within the three-dimensional structure of the ubiquitin molecule (**b**). **a** The components and processes of the UPS: this panel illustrates the key components involved in the UPS, which regulates protein degradation within cells. The process begins with the ubiquitin molecule, a small 76-amino acid protein that is activated by the ubiquitin-activating enzyme (E1) in an ATP-dependent manner. The activated ubiquitin is then transferred to the ubiquitin-conjugating enzyme (E2). The E2 enzyme, in collaboration with the ubiquitin ligase (E3), facilitates the transfer of ubiquitin to the substrate protein, often through an isopeptide bond to a lysine residue. Polyubiquitination, the addition of multiple ubiquitin molecules, is critical for directing proteins to the proteasome for degradation. The 26S proteasome, composed of a 20S core particle and a 19S regulatory particle, recognizes polyubiquitinated proteins. The 19S regulatory complex unravels and translocates the substrate into the 20S core, where it is broken down into small peptides. **b** Specific locations of ubiquitin chain types within the three-dimensional structure of the ubiquitin molecule: this panel displays the detailed structure of the ubiquitin molecule, highlighting the positions of key lysine residues where polyubiquitin chains (PUCs) can form. The three-dimensional structure of ubiquitin reveals distinct lysine residues that serve as linkages for various types of PUCs, each of which is responsible for specific cellular outcomes. DUB deubiquitinating enzymes, IBR in-between-RING, JAMM c-Jun activation domain binding protein 1 (JAB1)/Mpr1p Pad1p N-terminal (MPN)/moloney murine leukemia virus 34 (MOV34), MINDY motif-interacting with ubiquitin-containing novel DUB family, MJD Machado–Josephin domain proteases, OUT ovarian tumor protease, SENP sentrin/SUMO-specific protease, Ub ubiquitin, Uba1 ubiquitin-like modifier-activating enzyme 1, UCH ubiquitin carboxy-terminal hydrolase, UPS ubiquitin‒proteasome system, USP ubiquitin-specific protease, RING really interesting new gene, HECT homologous to the E6-AP carboxyl terminus. (https://app.biorender.com)

The concept of protein turnover was introduced just over 80 years ago, with the discovery of the lysosome marking a pivotal moment in the study of protein degradation.^1,2^ This discovery led to the hypothesis that proteins in the cells are degraded within this organelle.^3^ However, subsequent experimental findings indicated that nonlysosomal pathways are also involved in intracellular proteolysis.^3^ The identification of the ubiquitin‒proteasome system (UPS) ultimately resolved this issue.^3^ The UPS, which comprises ubiquitin (Ub) and the proteasome, accounts for approximately 80–90% of cellular proteolysis, whereas autophagy is responsible for the remaining 10–20%.^4^ Ub is a highly conserved protein found across eukaryotic tissues.^5,6^ The ubiquitination process is a highly specific, ATP-dependent mechanism that tags substrate proteins with Ub.^7^ Traditionally, protein modification by Ub involves a cascade of enzymatic activities: activation by E1 enzymes, conjugation by E2 enzymes, and ligation to target proteins by E3 ligases. This process is complemented by deubiquitinating enzymes (DUBs) that remove Ub molecules and chains from these substrates^8^ (Fig. 1a).

As illustrated in Fig. 1a, the 26S proteasome is a large multisubunit complex composed of the core protease, the 20S proteasome, and two symmetrically positioned 19S regulatory particles.^9^ The 19S regulatory particle contains a heterohexameric AAA+ ATPase ring composed of PSMC1, PSMC2, PSMC3, PSMC4, PSMC5, and PSMC6, which is essential for unfolding and translocating proteasomal substrates to proteolytically active sites.^10^ Four rings, each composed of seven subunits, assemble into a cylindrical structure to form the 20S proteasome.^11^ In the archaeon *Thermoplasma acidophilum*, a proteasome-like enzyme has also been identified.^11^ This enzyme comprises two types of related subunits, α and β, with the α subunits located in the outer two heptameric rings and the β subunits positioned in the inner rings.^11^ The proteolytic activities of the 20S catalytic particle are mediated by the β1 (also known as δ, PSMB6, and Y), β2 (known as MC14, PsMB7, and Z), and β5 (known as ε, MB1, X, and PSMB5) subunits, with the β5 subunit serving as a key rate-limiting factor.^10^ Given the need for the eukaryotic 20S proteasome to interact with diverse regulatory complexes at both termini of the proteasome cylinder, increased variability within the α subunits may allow interaction with a broader range of regulatory elements.^12^ In contrast, the incorporation of multiple β subunits may enable precise control over proteolytic specificity or increase the efficiency of substrate degradation across a broader spectrum.^12^

In vertebrates, the proteasome has several isoforms: the constitutive proteasome (cCP), which is found in all cells; the immunoproteasome (iCP), which is present mainly in immune cells; and the thymoproteasome (tCP), which is restricted to cortical thymic epithelial cells.^13^ The iCP is distinguished from the cCP by the replacement of its catalytic subunits with β1i (also termed LMP2 and PSMB9), β2i (also known as MeCL-1, LMP10, and PSMB10), and β5i (also known as LMP7 and PSMB8).^13^ While β2i and β5i retain the same proteolytic activities, β1i exhibits chymotrypsin-like activity. The proteolytic subunits of tCP are β1i, β2i, and a distinct β5t subunit (PSMB11).^13^ The iCP primarily processes antigen peptides for presentation *via* major histocompatibility complex class I (MHC I) molecules.^14^ This process is triggered by an influx of lymphocytes releasing high levels of interferon (IFN)-γ, which induces iCP production.^14^ However, in hematological cancers such as multiple myeloma (MM) and leukemia, iCP overexpression occurs without lymphocyte activation, as hematopoietic cells, both malignant and nonmalignant immune cells, directly produce iCP.^14^ The formation of iCPs and the PA28 activator complex, which is induced by IFN-γ, significantly enhances proteasome-mediated epitope processing, particularly for viral antigens.^15^ However, this effect does not generally apply to tumors or self-antigens, as immunoproteasomes often degrade or destroy epitopes derived from these proteins. Kloetzel PM and colleagues also identified a unique role for LMP2 (β1i) in primary Sjögren’s syndrome pathogenesis.^16^ They also concluded that the differences in MHC I-restricted antigen presentation between standard and iCPs are due to variations in the antigenic peptides generated by the proteasome.^17^ Importantly, defects in the subunits of the core particle (20S proteasome), such as PSMB8, PSMG2, POMP, PSMB10, PSMB9, PSMB4, and PSMA3, result in chronic atypical neutrophilic dermatosis with lipodystrophy and elevated temperature (CANDLE)/proteasome-associated autoinflammatory syndrome (PRAAS),^18^ which is thoroughly discussed in the “Autoinflammatory diseases” section.

In 1996, defective ribosomal products (DRiPs) were introduced to explain the rapid MHC I-mediated presentation of viral peptides to CD8⁺ T cells.^19^ DRiPs are polypeptides derived from in-frame translation events that fail to attain proper conformations due to intrinsic errors in transcription, translation, posttranslational modifications (PTMs), or protein folding.^19^ DRiPs exhibit exceptionally short half-lives (<10 min) and are estimated to represent ~30% of all polypeptides synthesized by ribosomes.^20^ Additionally, it has been suggested that some DRiPs are ubiquitinated, with ubiquitinated DRiPs originating from the human immunodeficiency virus Gag polyprotein, a relatively long-lived viral protein that serves as an antigenic peptide source.^21^

Antigen processing occurs via two classical pathways: endogenous processing, which leads to presentation *via* MHC I, and exogenous processing, which involves MHC-II molecules.^22^ Rock KL and colleagues proposed that the processing of endogenous antigens occurs in a series of steps, starting with the degradation of proteins into peptide fragments via the UPS.^23^ While most of these peptides are further processed into amino acids by peptidases, a small fraction is transported into the endoplasmic reticulum (ER) lumen via transporters associated with antigen processing (TAP) transporters.^23^ Within the ER, these peptides are trimmed to 8–10 amino acid fragments by ER aminopeptidase-1 (ERAP-1).^24^ These fragments then bind to newly synthesized MHC I molecules and are presented to CD8 + T cells.^23^ Thus, enhancing the efficiency of ubiquitin‒proteasome-mediated antigen degradation can increase MHC I-mediated antigen presentation and the CD8 + T-cell immune response.^25^ For example, Ling J et al. developed a novel mRNA vaccine by conjugating the ALAPYIP peptide sequence with antigens to promote antigen degradation via the UPS, thereby enhancing antigen presentation and the CD8 + T-cell immune response. To overcome the intrinsic instability of mRNAs and improve their cellular uptake, advanced lipid nanoparticles (LNPs) have been utilized as delivery systems.^26^ This approach demonstrates the potential of harnessing the UPS to improve immune responses, offering a promising strategy for developing more effective vaccines and immunotherapies.

Ubiquitination is a PTM that is essential for regulating protein quality and quantity.^27^ Among the most prevalent PTMs are ubiquitination, nitration, oxidation, methylation, and phosphorylation. Each of these modifications significantly expands the proteome.^27^ Ubiquitination, the second most common PTM after phosphorylation, involves the attachment of a Ub molecule or Ub chains to substrates.^28^ This process generates conjugates with distinct topologies that modulate the localization, interactions, stability, and activity of thousands of target proteins.^29^ Disruption of ubiquitination can lead to protein mislocalization, misfolded or damaged protein accumulation, defective complex assembly, aberrant enzymatic activity, or impaired signal transduction events.^30^

Ubiquitination regulates cell death, cell metabolism, DNA damage repair, and immunity.^27^ The expanding roles of deubiquitination and ubiquitination in regulating immune signaling pathways such as nuclear factor kappa-B (NF-κB),^31^ toll-like receptor (TLR), retinoic acid-inducible gene I (RIG-I)-like receptor (RLR), and stimulator of IFN genes (STING)-dependent pathways,^32^ as well as RAS, including mitogen-activated protein kinase (MAPK) and phosphoinositide 3-kinase-Ak strain transformation (PI3K-AKT)-mTOR,^33^ Wnt/*β*-catenin,^34^ Hippo pathways,^35^ and transforming growth factor-beta (TGF-β) pathways,^36^ alongside DNA damage repair signaling,^37^ have garnered increasing attention. For example, in the canonical NF-κB pathway, activation of the IκB kinase (IKK) complex results in the phosphorylation of IκB, leading to its ubiquitination and proteasomal degradation.^31^ Among immune signaling pathways, the TLR family consists of 13 members.^38^ For example, in the TLR4 signaling pathway, USP4 specifically interacts with TGF-β-activated kinase 1 (TAK1) and stabilizes its protein levels through deubiquitination.^38^ Various types of polyubiquitination (polyUb) on STING, which is mediated by ubiquitin ligases, have also been identified, including K27, K48, K63, and K11 modifications.^39^ In conventional MAPK cascades, signaling components are regulated both directly and indirectly through ubiquitination and deubiquitination, the latter facilitated by DUBs.^40^ Aberrations in ubiquitination and deubiquitination within the PI3K/AKT/mTOR signaling pathway are common in various diseases, underscoring the necessity for tight regulation of this pathway.^41^ When the Hippo pathway is activated (i.e., when kinases are phosphorylated and active), LATS1/2 phosphorylate Yes-associated protein (YAP)/transcriptional coactivator with a PDZ-binding motif (TAZ) at multiple sites, triggering their polyUb and subsequent degradation, thereby further regulating the Hippo pathway.^42^ In the TGF-β signaling pathway, OTUD1 regulates both SMAD3 and SMAD7 through distinct mechanisms: it enhances SMAD3/SMAD4 complex formation by removing Ub chains from SMAD3, promoting its nuclear translocation and transcriptional activity, and stabilizing the negative regulator SMAD7 by cleaving its Ub chains.^43^ Ub signaling coordinates and regulates cellular responses to double-strand breaks (DSBs) at multiple levels. For example, protein recruitment to DSB sites is mediated by the RNF8–RNF168 E3 Ub ligase pathway.^37^ However, abnormalities in the biological processes and pathways regulated by ubiquitination can lead to cancer, neurodegenerative disorders (NDDs), cardiovascular diseases (CVDs), inflammatory disorders, autoinflammatory disorders and developmental diseases. Consequently, targeting ubiquitination offers promising avenues for disease treatment.

In this review, we summarize the research history and milestones in the study of ubiquitination in health and disease. We discuss the components and processes, signaling pathways and crosstalk, multilevel regulatory mechanisms, and functions of ubiquitination in pathophysiological conditions. Additionally, we review and discuss therapeutic targets and clinical research progress, including clinical trials and FDA-approved drugs, aimed at targeting ubiquitination for the treatment of various diseases.

## From lysosomes to the UPS: evolution of the current targeted protein degradation technology

**Fig. 2:**
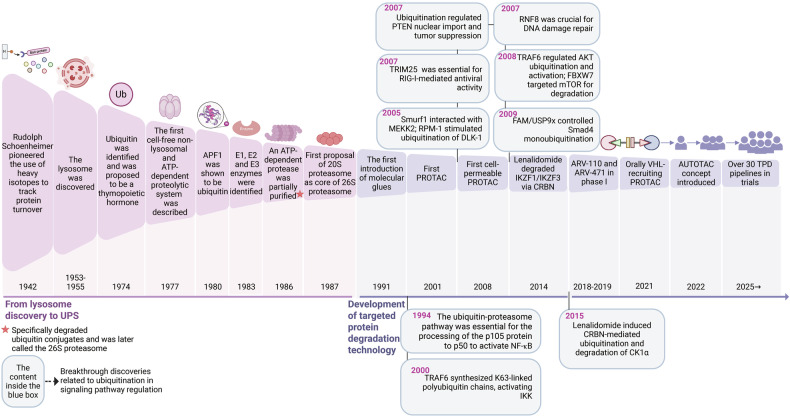
Timeline of the most important discoveries over the past eight decades, highlighting advances in intracellular protein degradation, TPD technology, and breakthrough findings related to ubiquitination in the regulation of signaling pathways. APF-1 ATP-dependent proteolysis factor 1, AUTOTAC autophagy-targeting chimera, PROTAC proteolysis-targeting chimera, TPD targeted protein degradation, UPS ubiquitin‒proteasome system, VHL von Hippel‒Lindau. The elements were obtained from BioRender.com

Over the past 80 years, protein biology has undergone remarkable evolution, beginning with the groundbreaking discovery of the dynamic nature of proteins, followed by the identification of the lysosome and the elucidation of the UPS. These foundational discoveries paved the way for the development of proteolysis-targeting chimera (PROTAC) technology, which has since transitioned from academic research to industrial application. Today, numerous biotech and pharmaceutical companies have advanced targeted protein degradation (TPD)-based programs into preclinical and clinical development, marking a transformative shift in therapeutic innovation and opening new frontiers in drug discovery (Fig. 2).

In 1942, Rudolph Schoenheimer’s seminal work was published, detailing the use of heavy isotopes to track protein turnover and laying the foundation for understanding protein dynamics.^3^ Between 1953 and 1955, the discovery of the lysosome led to the proposal that protein degradation in mammalian cells is an energy-dependent process.^1,2,44^ However, the concept of protein turnover remained contentious, with significant skepticism persisting into the mid-1950s. For example, Hogness and colleagues demonstrated that proteins in growing *E. coli* were stable, suggesting in 1955 that proteins in mammalian cells might also exhibit similar stability.^45,46^ By 1958, it was established that protein degradation in bacteria also requires metabolic energy, further solidifying the understanding of energy-dependent proteolysis.^47^

In 1974–1975, Ub (initially termed ubiquitous immunopoietic polypeptide, or UBIP) was discovered and proposed to function as a thymopoietic hormone.^48–50^ Studies between 1976 and 1979 revealed that lysosomes are not the principal site for basal intracellular protein degradation. It has been proposed that cells possess two distinct proteolytic systems: lysosomes, which degrade extracellular proteins, and an unidentified system, which is responsible for intracellular protein degradation.^51,52^ In 1977, the discovery of an isopeptide bond linking Ub to histone 2A in chromatin provided key insight into the cellular role of Ub.^53,54^ In the same year, a nonlysosomal, ATP-dependent proteolytic system was isolated from reticulocytes, providing a foundational model for investigating Ub-mediated protein degradation.^55^

In 1978, this ATP-dependent proteolytic activity was fractionated into two components: Fraction I, a heat-stable, nondialyzable protein with a molecular weight of ~9000, and Fraction II, which exhibited weak ATP-dependent neutral protease activity.^56^ By 1980, it was demonstrated that protein degradation required the conjugation of cellular substrates with multiple copies of heat-stable ATP-dependent proteolysis factor 1 (APF-1).^57,58^ In 1980, Ciechanover et al. confirmed that APF-1 exhibited Ub-like characteristics.^59^ That same year, AL Hass was the first to identify heat-stable polypeptides as Ub.^60^

The “multicatalytic proteinase complex” was identified in the early 1980s and later confirmed in 1987 to be the 20S core particle of the 26S proteasome.^61,62^ In 1983, three enzymes essential for Ub conjugation—E1, E2, and E3—were isolated, elucidating the enzymatic cascade of ubiquitination.^63^ An ATP-dependent protease specific for Ub-tagged substrates was partially purified and later confirmed in 1987 to be the 26S proteasome, completing the core framework of the UPS.^64^ In 2004, a report published in *Cell* by Pickart CM elucidated the mechanisms of E1/E2/E3-based ubiquitination.^65^

The therapeutic era of TPD began in 1991 with the introduction of the concept of molecular glues.^66^ A decade later, in 2001, the Crews group introduced PROTACs, which harness the UPS to degrade target proteins.^67^ The first PROTAC was engineered by covalently linking ovalicin, a small molecule that targets methionine aminopeptidase-2 (MetAP-2), to a 19-residue phosphopeptide degron derived from IκBα, a substrate of the Skp1-Cullin-F-box (SCF)-βTRCP E3 ligase. This construct facilitated the recruitment of MetAP-2 to SCF-βTRCP, inducing its ubiquitination and proteasomal degradation.^67–69^ However, the large molecular mass and high polarity of this PROTAC limit its cell permeability, prompting the development of more “drug-like” PROTACs using small-molecule E3 ligase recruiters.^70^ In 2008, the first small-molecule PROTAC, the SARM–nutlin PROTAC, was reported, enabling the recruitment of the androgen receptor (AR) to mouse double minute 2 (MDM2) and its subsequent degradation.^71,72^

In 2013, Arvinas was founded as the first company dedicated to developing PROTACs for cancer and neurological diseases.^73^ The following year, studies revealed that lenalidomide, a therapeutic agent for myeloma, induces cereblon (CRBN)-mediated degradation of the transcription factors IKZF3 and IKZF1, elucidating its mechanism of action in MM and other B-cell malignancies.^74,75^ In 2014, a novel peptide-based strategy was also introduced, leveraging the chaperone-mediated autophagy (CMA) pathway to enable the reversible, dose-dependent and time-dependent degradation of native proteins. This approach successfully targets proteins ranging from small (19 kDa, α-synuclein) to large (160 kDa, DAPK1), demonstrating its versatility.^76^

In 2015, C4 therapeutics were established to advance TPD technologies for cancer treatment.^73^ Since then, PROTAC development has increased, with degraders targeting critical proteins such as PI3K, ALK, and AR via E3 ligases such as CRBN, Von Hippel-Lindau (VHL), IAP, and MDM2.^77,78^ In 2018, the field of TPD achieved a pivotal milestone with the initiation of clinical trials for ARV-110, the first-in-class AR degrader. With this progress, both ARV-471 and ARV-110 advanced to phase I clinical trials in 2019, with ARV-110 further progressing to phase II trials the following year, underscoring the rapid clinical translation and therapeutic potential of AR-targeted degraders.^72^ The concepts of autophagosome-tethering compounds (ATTECs) and autophagy-targeting chimeras (AUTACs) were introduced in 2019, expanding the TPD toolkit.^79^ In 2021, an orally bioavailable VHL-recruiting PROTAC was developed, which demonstrated degradation of HMG-CoA reductase (HMGCR) and potent hypolipidemic activity in vivo.^80^ The concept of autophagy-targeting chimera (AUTOTAC) was introduced in 2022, further advancing autophagy-based therapeutic strategies.^73^

Today, TPD encompasses a diverse array of modalities, including PROTACs, molecular glues, lysosome-targeting chimeras (LYTACs), ATTECs, AUTACs, AUTOTACs, and degrader‒antibody conjugates (DACs). PROTAC degraders targeting proteins such as BCL-xL, IRAK4, STAT3, BTK, BRD9, and MDM2 are in clinical trials, highlighting the expanding therapeutic potential of this approach.^79^ Over 30 TPD-based pipelines are in clinical trials, predominantly in oncology, with promising applications in NDDs, autoimmune conditions, inflammatory diseases, and viral and bacterial infections, highlighting the potential of TPD in modern medicine.^79^

Notably, as shown in Fig. 2, breakthrough discoveries related to the role of ubiquitination in signaling pathway regulation have been reported. In 1994, Palombella VJ and colleagues identified the crucial function of the proteasome complex in two proteolytic steps needed for NF-κB activation.^81^ They showed that p105, the precursor of the p50 subunit, is processed through an ATP-dependent mechanism requiring both proteasome and Ub conjugation.^81^ The C-terminal portion of p105 is degraded, enabling the release of the N-terminal p50 domain and the subsequent activation of NF-κB.^81^ Owing to space limitations, other significant breakthroughs are detailed in the relevant sections of the ubiquitination-regulated signaling pathways.

## The components and processes of the UPS

### Ub

The human genome contains four genes responsible for encoding ubiquitin: RPS27A, UBA52, UBC, and UBB.^82^ The RPS27A and UBA52 genes each encode a single ubiquitin unit fused to the N-terminal region of the ribosomal proteins S27a and L40, respectively.^82^ In contrast, UBC and UBB give rise to polyubiquitin precursors, with UBB comprising three tandem ubiquitin repeats and UBC containing nine.^82^ Ubiquitin is synthesized either by ribosomal fusion—mediated by UBA52 and RPS27A—or by the action of DUBs, which cleave polyubiquitin chains encoded by UBC and UBB to generate free ubiquitin.^27^ Ub is characterized by seven lysine residues and one N-terminal methionine (Lys48, Lys63, Lys6, Lys11, Lys27, Lys29, Lys33, or Met1), which act as attachment points for polyubiquitin chains (PUCs), facilitating a range of conformations and interactions with target proteins.^83^ These lysine residues are crucial for forming isopeptide-linked Ub chains, with Lys48-linked chains being the most prevalent (comprising over 50% of linkages) and primarily involved in targeting proteins for proteasomal degradation.^5,84^ On the other hand, Lys63-linked chains, the second most common type,^85^ serve various nondegradative functions.^86^ Additionally, an eighth type of chain, referred to as Met1-linked or “linear” chains, is formed when the N-terminus of one Ub is linked to the N-terminus of another.^87^ More strikingly, the Ub molecule itself, a PTM, is subject to distinct PTMs, including phosphorylation and acetylation. Mining of available datasets revealed that six out of seven lysine residues in Ub are susceptible to acetylation.^88–90^ The phosphorylation of Ub at Ser65 is essential for mitophagy, whereas Ser57 phosphorylation was identified in early Ub proteomics studies.^87,91–93^ Ub can also be phosphorylated at additional sites, including Thr66, Tyr59, Ser20, Thr14, Thr12, and Thr7. These phosphorylation events, whether on monoubiquitin or PUCs, can influence their recognition by Ub-binding proteins or E3 ligases.^94^ Moreover, acetylation of Ub regulates various cellular processes, such as actin nucleation, nuclear transport, splicing, cell cycle progression, chromatin remodeling, and muscle contraction.^95^ These features collectively contribute to the complexity of the Ub code, encompassing three critical structural parameters: chain length, linkage heterogeneity, and branching architecture.

### Ubiquitination

Ubiquitination governs proteostasis in eukaryotic systems through covalent modification of substrate proteins with Ub.^96^ This versatile PTM dynamically regulates protein fate by directing substrates toward degradation, modulating subcellular trafficking, or altering functional states. The modification cascade is mediated through the coordinated actions of three enzyme classes, as shown in Fig. 1a.^8^ The human genome maintains a restricted repertoire of E1 ubiquitin-activating enzymes, with only two evolutionarily conserved members—ubiquitin-like modifier-activating enzyme 1 (Uba1) and Uba6—identified across vertebrate lineages (Fig. 1a).^97^ Mutations in the *UBA1* gene give rise to vacuoles, the E1 enzyme, and X-linked, autoinflammatory, somatic (VEXAS) syndrome,^98^ which is further described in the “Autoinflammatory disorders” section. The human Ub system employs 38 distinct E2-conjugating enzymes, all of which harbor a conserved catalytic ubiquitin-binding domain yet exhibit remarkable partner specificity (Fig. 1a).^99^ Over 600 E3 ligases—the most diverse components of the UPS—coordinate substrate recognition and linkage determination, with most regulatory contexts requiring E3-mediated catalysis despite limited autonomous E2 transfer capacity (Fig. 1a).^100^

### Ubiquitination linkage

Figure 1a illustrates four principal Ub linkage architectures: monoubiquitination, canonical polyUb, linear ubiquitin assembly complex (LUBAC)-mediated M1-linked chains, and topologically complex branched polymers.^101^ Monoubiquitination dynamically modulates protein conformation, catalytic activity, interactome networks, and spatial partitioning to orchestrate critical cellular functions spanning membrane trafficking, genome integrity, and chromatin topology.^102^ For example, monoubiquitination coordinates DNA repair through chromatin remodeling and repair factor recruitment. H2B ubiquitination exemplifies this regulation by enabling nascent D-loop formation via histone eviction while stabilizing recombination intermediates through nucleosome reorganization coupled with Rad9-mediated resection control to prevent genomic instability.^103^ H2BK120 ubiquitination drives chromatin epigenetic regulation,^104^ whereas multisite monoubiquitination of Ras coordinates the MAPK and PI3K-AKT signaling axes.^105^

In contrast to monoubiquitination, polyUb is remarkably diverse, as Ub molecules can be interconnected through any of their seven lysine residues or via the N-terminal methionine (Met1).^106^ Early paradigms positioned K48-linked ubiquitination as the archetypal proteasomal degradation signal, representing the sole characterized polyubiquitin topology. Subsequent systems-level analyses revealed an expanding Ub code, with eight conserved linkage types (Met1, K63, K48, K33, K29, K27, K11, and K6) now mapped to distinct cellular functions—from K63-mediated kinase activation cascades to Met1-linear chain immune signaling.^86,107^ Figure 1b shows the positions of the eight types of Ub chains within the three-dimensional structure of the Ub molecule. In addition to the canonical proteolytic role of K48-linked ubiquitination, the Ub code exhibits remarkable topological plasticity in cellular regulation. K6-linked chains orchestrate homology-directed repair,^108^ whereas K11-specific linkages license mitotic checkpoint control through anaphase-promoting complex/cyclosome (APC/C)-mediated substrate recognition.^109^ Mitochondrial quality control is governed by K27-autophagic flux coordination,^110^ in contrast with K29-channels, which gatekeeper ubiquitin-fusion degradation (UFD) pathway selectivity.^111^ In immune modulation, K33-linked polymers fine-tune TLR signaling cascades,^112^ whereas K63-chromatin scaffolds integrate kinase activation hubs.^113^ Met1-linear chains—exclusively assembled by the LUBAC complex—license NF-κB signalosome activation through N-terminal Ub conjugation, establishing a paradigm for linear Ub signaling in TNFα responses.^114^ This topological plasticity establishes Ub as a master regulator of cellular signaling fidelity, with chain architecture dictating functional specificity across biological systems.

Branched polyUb has emerged as a critical regulatory mechanism in various cellular processes. For example, branched Ub chains incorporating both K11- and K63-linked Ub moieties have been shown to facilitate Epsin1-mediated endocytosis of MHC I,^115^ highlighting the functional complexity and specificity conferred by these unique chain architectures.

### E3 ligases

E3 Ub ligases play a key role in determining the specificity of substrate ubiquitination, orchestrating Ub attachment to target proteins with remarkable precision. On the basis of their catalytic mechanisms and structural features, E3 ligases are traditionally classified into three major families: the homologous to the E6-AP carboxyl terminus (HECT) family; the really interesting new gene (RING) family; and the RING homology-in-between-RING (RBR) family, which combines features of both HECT and RING mechanisms, utilizing a hybrid approach to catalyze ubiquitination (Fig. 1a).^115^ These distinct families exemplify the structural and functional diversity of E3 ligases, underscoring their critical role in regulating cellular processes through targeted ubiquitination.

#### RING-type E3 ligases

As illustrated in Fig. 1a, the human genome encodes over 600 E3 Ub ligases, a number that surpasses even the 518 protein kinase genes, underscoring their extensive regulatory potential in cellular processes.^116^ Among these, the RING family is the most extensive and heterogeneous, comprising ~270 members.^117^ The typical RING finger domain is a zinc (Zn²⁺)-binding domain characterized by a conserved arrangement of histidine (His) and cysteine (Cys) residues spaced at specific intervals.^118^ This domain plays a critical role in mediating E2-dependent ubiquitination by facilitating the direct transfer of Ub from E2 enzymes to substrate proteins, thereby maintaining the fidelity of the ubiquitin-regulated pathway.^118^ RING finger E3 ligases demonstrate significant structural and functional adaptability, functioning as monomers, dimers, or components of larger multisubunit assemblies.^117^ RING domain-mediated dimerization enables both homomeric (e.g., cIAP1/BIRC2 in apoptosis regulation, RNF4 in small ubiquitin-like modifier (SUMO)-targeted degradation) and heteromeric (e.g., MDM2-HDMX in p53 destabilization, BRCA1–BARD1 in DNA repair) assemblies.^119–122^ The MDM2-HDMX heterodimer exemplifies this paradigm, coordinating K48-linked ubiquitination of p53 through complementary RING domain interactions,^123^ whereas RNF152 exemplifies the functional specificity of K63-linked ubiquitination by modulating mTORC1 signaling through the precise modification of RagA.^124^ Diverging from dimeric architectures, multisubunit E3 complexes such as SCFs and APC/Cs integrate catalytic and substrate-recognition modules into highly organized structures. The SCF complex features a modular design comprising (1) Rbx1 for E2 (UBE2M/R) recruitment; (2) Cul1 as a scaffold; (3) Skp1 as an adapter; and (4) F-box proteins (69 human variants) that determine substrate specificity.^125^ F-box and WD repeat domain-containing 7 (FBXW7), a tumor-suppressive F-box protein, exemplifies this mechanism by targeting phosphorylated degrons in oncoproteins such as epidermal growth factor receptor (EGFR) and c-Myc.^126,127^ Conversely, the oncogenic F-box protein SKP2 drives cell cycle progression by mediating the SCF-dependent degradation of CDK inhibitors, including p27 and p21.^128^ The APC/C complex orchestrates precise cell cycle transitions through timed coactivator switching: CDC20 activates the metaphase-to-anaphase transition by targeting securin for degradation,^129^ whereas CDH1 terminates mitosis by eliminating Cyclin B1 and Aurora kinases.^130^ These architectural innovations—ranging from dimeric specificity to modular complexity—establish ubiquitination as a master regulatory mechanism in cellular homeostasis.

#### HECT-type E3 ligases

Diverging from RING-type E3 ligases, the HECT family employs a catalytic HECT domain that transiently accepts Ub through a conserved Cys residue before transferring it to substrate proteins (Fig. 1a). This evolutionarily conserved family is stratified into three functional subclasses: (1) neuronal precursor cell-expressed developmentally downregulated 4 (NEDD4)/NEDD4-like E3s, which contain WW domains (tryptophan-rich β-sheet motifs) that bind PY motifs in targets such as ion channels; (2) HERC E3s, characterized by RCC1-like domains (RLDs) important for membrane binding and GTPase control; and (3) noncanonical HECT E3s, which lack both WW and RLD motifs and include HUWE1, a crucial E3 ligase that regulates MYC protein turnover.^131^ Genomic studies indicate that the human genome encodes approximately 30 HECT E3 genes, markedly fewer than the more than 600 genes encoding RING-type E3s, indicating their distinct evolutionary histories and specialized roles.^117^

The HECT family illustrates catalytic adaptability through conserved C-terminal domains that allosterically affect ubiquitin chain architecture, setting them apart from RING-type E3s, which depend on E2 enzyme specificity.^132^ E6-AP/UBE3A pioneers this paradigm: its HECT domain (with the Cys843 catalytic triad) mediates HPV E6-driven K48-linked p53 degradation. Additionally, genomic imprinting defects in UBE3A underlie Angelman syndrome (AS),^133,134^ a developmental disorder that will be discussed in detail in the “Developmental abnormalities” section. NEDD4 subfamily members have divergent specializations—Rsp5’s C-terminal β-hairpin configures K63 chains for endocytic trafficking.^133,135^ This structural adaptability extends to NEDD4’s WW domain-mediated phosphoinositide recognition, where its β2-β3 loop positions K63 chains on PTEN to regulate membrane turnover,^136^ whereas WWP2’s tandem WW domains recruit LATS1 phosphodegrons to coordinate Hippo-autophagy integration.^137^ This functional dichotomy—from viral oncoprotein hijacking to phosphoinositide metabolic control—stems from evolutionary sculpting of three architectural modules: C2 membrane-targeting domains, WW substrate readers, and HECT catalytic engines, collectively establishing HECT ligases as sentinels of cellular homeostasis through chain topology-encoded signaling.

#### RBR-type E3 ligases

RBR E3 Ub ligases were initially characterized via sequence analysis, which revealed a unique three-part structure consisting of zinc (Zn²⁺)-binding domains: two typical RING domains (RING2 and RING1) separated by an in-between-RING (IBR) domain.^138^ Like the canonical RING domains, the RING1 domain within the RBR module binds to E2 enzymes conjugated with Ub (E2~Ub). In contrast, the RING2 domain harbors a critical active-site Cys residue that accepts Ub from the E2~Ub complex, forming an E3~Ub intermediate—a mechanism that is otherwise exclusive to HECT-type E3 ligases.^139^ These dual characteristics establish RBR E3s as RING–HECT hybrids, combining features of both families to achieve Ub transfer. This hybrid mechanism was first elucidated for Parkin and HHARI, and subsequent studies have confirmed the functional importance of the active-site Cys for catalytic activity in other RBR family members, including TRIAD1, HOIP, HOIL-1 L, and RNF144A.^140–142^ The presence of this Cys is now recognized as a defining feature of RBR E3 ligases, highlighting their unique role in ubiquitin-dependent cellular regulation.

Among the RBR family, LUBAC stands out as a particularly striking example. LUBAC is a multisubunit E3 ligase complex composed of Sharpin, HOIL-1 L, and HOIP.^142^ It specializes in the assembly of linear (M1-linked) Ub chains, which play critical roles in regulating NF-κB activation and cell death mechanisms.^143^ The unique ability of LUBAC to generate linear Ub chains underscores the functional versatility and biological importance of RBR E3 ligases in orchestrating complex cellular processes.

### Nonclassical ubiquitination

Qui et al. revealed a groundbreaking noncanonical ubiquitination mechanism orchestrated by the SidE family of effector proteins in *Legionella pneumophila*. The mono-ADP-ribosyltransferase (mART) domain of SidE catalyzes the NAD⁺-dependent ADP-ribosylation of ubiquitin (ADPr-Ub) at Arg_42_. The phosphodiesterase (PDE) domain subsequently cleaves the phosphodiester bond of ADPr-Ub, generating phosphoribosylated ubiquitin (Pr-Ub). This Pr-Ub is then covalently attached to serine (Ser) residues of the substrate Rab33 through a transesterification reaction.^144–146^ High-resolution crystal structures capture key intermediates: the prereaction state (apo-SidE), the mART-Ub-NAD complex (representing the first-step reaction), and the PDE-Ub-ADP-ribose complex (representing the second-step reaction).^147^

In parallel, another *L. pneumophila* effector, MavC, facilitates a transglutamination reaction between Ub’s Q40 and K92 (and less frequently K94) of the host E2 enzyme UBE2N (also known as ubiquitin-conjugating enzyme 13, UBC13), forming an isopeptide bond.^148^ Furthermore, the *Mycobacterium tuberculosis* effector PknG independently executes ATP-dependent ubiquitination by conjugating G76 of Ub to K82 of the host E2 enzyme UBE2L3 (UbcH7), with concurrent hydrolysis of ATP at the γ-phosphate.^149^ These findings collectively expand our understanding of the diverse and sophisticated strategies employed by bacterial pathogens to hijack the host ubiquitination machinery.

### Ub-like proteins

Ubiquitin-like proteins (UBLs) represent a structurally conserved family of small protein modifiers that functionally parallel Ub through covalent attachment to target substrates. Like ubiquitination, most UBL modifications involve a three-step enzymatic cascade: E1 UBL-activating enzymes, E2 UBL carrier proteins, and E3 UBL ligases. Over a dozen UBLs have been characterized, including SUMO1-4 (involved in nuclear processes), NEDD8 (regulates cullin-RING ligases), ISG15 (antiviral response), Atg8/Atg12 (autophagy), FAT10 (innate immune response), Ufm1 (ER-phagy and ribosome-associated quality control), and Urm1 (a regulator of oxidative stress).^150^ These UBLs exhibit diverse modes of conjugation—some form polymeric chains (e.g., SUMO chains), whereas others attach as monomers—to modulate protein localization, stability, or interaction networks across cellular pathways. These processes, while distinct from ubiquitination, share mechanistic similarities and significantly influence protein function, stability, and interactions. Given the large number of relevant publications and space limitations, we are unable to provide a detailed discussion of these molecules.

### DUBs

Deubiquitination refers to the removal of Ub from substrate proteins, a tightly regulated enzymatic process mediated by DUBs^151^ (Fig. 1a). More than 100 DUBs have been characterized and are broadly categorized into two main subclasses: metalloproteases and cysteine proteases (CPs). The CP group comprises eight distinct families: permuted papain-fold peptidases of dsDNA viruses and eukaryotes (PPPDEs), monocyte chemotactic protein-induced proteins (MCPIPs), the MIU-containing novel DUB family (MINDY), Machado–Joseph disease protein domain proteases (MJDs), Otubain domain-containing ubiquitin-binding proteins (OTUs), ubiquitin C-terminal hydrolases (UCHs), ubiquitin-specific proteases (USPs), and zinc finger with UFM1-specific peptidase domain proteins (ZUFSPs). The metalloprotease subclass is represented by the Jab1/MPN domain-associated metalloisopeptidase (JAMM) family.^152^ The deubiquitination of CPs is driven by a catalytic triad consisting of three conserved amino acids: His, Cys, and asparagine/aspartic acid (Asn/Asp). In contrast, the deubiquitination activity of metallopeptidases depends on the coordination of Ser, aspartic acid, and His residues with zinc ions, highlighting the distinct yet precise molecular strategies employed by these enzyme classes to regulate Ub signaling.^153^ These enzymes play critical roles in modulating protein degradation by cleaving Ub chains, thereby preventing proteasomal targeting. They also recycle Ub by breaking down polyubiquitin into monomers, ensuring the availability of free Ub for subsequent signaling and regulatory processes.

The intracellular functions of DUBs extend far beyond their role in proteosomal regulation. They are integral to cell cycle regulation, protein trafficking, the DNA damage response (DDR), and the modulation of gene expression. This multifaceted involvement underscores their essential contribution to maintaining cellular homeostasis and orchestrating complex biological pathways.^154^ For example, USP8 and USP32 play pivotal roles in protein trafficking by regulating key factors such as EGFR and Rab7, respectively.^155,156^ DUBs also affect the ataxia-telangiectasia mutated (ATM) kinase–mediated DDR pathway by acting on downstream targets such as ATR, CHK2, CHK1, and 53BP1.^157,158^ Moreover, USP11 and USP9X control gene expression by regulating the stability of the translation initiation factors eIF4B and eIF4A, respectively.^159,160^ Furthermore, USP22 regulates cell proliferation through the deubiquitination of far upstream element (FUSE)-binding protein 1 (FBP1), highlighting the diverse and essential roles of DUBs in cellular signaling and homeostasis.^161^

## Ubiquitination and deubiquitination: pathways and crosstalk in cell signaling

### The NF-κB signaling pathway and its regulation by ubiquitination and deubiquitination

**Fig. 3:**
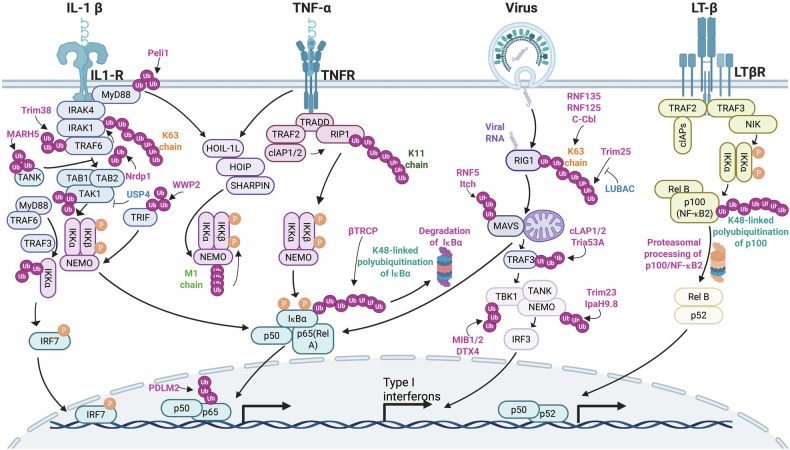
Ubiquitination and deubiquitination in the NF-κB signaling pathway and its crosstalk with the Toll-like receptor (TLR) and retinoic acid-inducible gene I (RIG-I)-like receptor (RLR) signaling pathways. This figure provides a comprehensive overview of the roles of ubiquitination and deubiquitination in regulating the NF-κB signaling pathway and its intricate crosstalk with the TLR and RLR signaling pathways, which are crucial for the immune response, inflammation, and pathogen recognition. The elements were obtained from BioRender.com

NF-κB is a dimeric TF consisting of proteins that contain a conserved Rel homology domain (RHD). Members of this family include p65 (RelA), c-Rel, RelB, and the precursor proteins p100 and p105, which are processed into p52 (NF-κB2) and p50 (NF-κB1), respectively (Fig. 3).^162^ In resting cells, NF-κB dimers are maintained in the cytoplasm through interactions with inhibitory IκB proteins, such as IκBε, IκBβ, and IκBα, as well as with the precursors p100 and p105.^162^ Upon cellular activation, IκB proteins are phosphorylated and subsequently ubiquitinated by the SCF-βTRCP E3 ligase. This ubiquitination marks IκBs for proteasomal degradation, thereby releasing NF-κB heterodimers to enter the nucleus and initiate gene transcription.^163^ This activation primarily occurs through the canonical and noncanonical NF-κB pathways.^162^

The canonical NF-κB pathway is activated by various stimuli, such as interleukin-1 beta (IL-1β) and TNF-α, as well as pathogen-associated molecular patterns (PAMPs).^164^ These signals lead to the activation of the IKK complex, which is composed of two kinase subunits—IKKβ and IKKα—and the regulatory component known as NF-κB essential modulator (NEMO) or IKKγ. Once activated, the IKK complex phosphorylates IκBα at two N-terminal Ser residues. This modification promotes its recognition and Ub-dependent degradation by the proteasome, allowing the p50/c-Rel and p50/p65 dimers to translocate into the nucleus, where they serve as principal transcriptional regulators in this pathway (Fig. 3).^165^

On the other hand, the noncanonical NF-κB pathway is activated by a more limited set of stimuli, including ligands of the tumor necrosis factor receptor (TNFR) superfamily, such as the lymphotoxin β receptor (LT-βR).^164^ In the absence of ligand‒receptor stimulation, TNFR-associated factor 2 and 3 (TRAF2 and TRAF3) downregulate NF-κB-inducing kinase (NIK) levels by promoting its ubiquitination and degradation. This continuous degradation, marked by lysine 48-linked Ub chains, prevents NIK accumulation.^166^ Upon ligand‒receptor activation via the noncanonical pathway, the cellular inhibitor of apoptosis protein (cIAP) catalyzes the lysine 48-linked ubiquitination and degradation of TRAF3.^166^

Unlike the canonical pathway, the noncanonical pathway does not involve IκBα degradation. Instead, it relies on processing the NF-κB2 precursor p100 into its mature form, p52.^167^ Upon stimulation, NIK activates IKKα, which then phosphorylates p100 ^166^. IKKα phosphorylates Ser residues 870 and 866 near the C-terminus of p100, creating a binding site for the SCF complex βTRCP Ub E3 ligase.^166^ Ubiquitination at lysine 856 results in polyUb, which triggers the limited proteasomal degradation of p100 at the C-terminus, ultimately generating the functionally active TF p52.^166^ This phosphorylation induces polyUb and partial degradation of the C-terminal ankyrin repeats of p100, resulting in the generation of p52.^167^ The p52 protein forms a dimer with RelB, and this complex enters the nucleus to regulate gene expression (Fig. 3).^167^

Although these pathways operate through distinct mechanisms, both are critically regulated by ubiquitination. Various forms of ubiquitination tightly modulate NF-κB signaling, playing a central role in its activation.^168,169^ Their regulation is intricately controlled by several types of polyUbs, including Met1-linked, K63-linked, K48-linked, and unanchored PUCs.^164^ As illustrated in Fig. 3, the noncanonical pathway is predominantly regulated by K48-linked PUCs, which drive the proteasomal degradation of p100, thereby activating downstream signaling. In contrast, the canonical pathway utilizes a more diverse array of Ub chain types, each of which plays a distinct role in fine-tuning signaling events. For example, the degradation of IκBα is mediated by K48-linked polyUb facilitated by βTRCP, whereas IKK complex activation and NF-κB nuclear translocation involve other Ub linkages. This intricate diversity in Ub chain types highlights the complexity and adaptability of ubiquitin-dependent mechanisms in regulating NF-κB signaling pathways.^168^ A20 serves as a negative regulator by removing K63-linked and linear Ub chains from target proteins, such as IKKγ and RIPK1.^170^ Patients with haploinsufficiency of A20 (HA20) have been identified in cases of familial autoinflammatory syndromes with Behçet-like features.^171^ This condition is discussed in detail in the “Autoinflammatory disorders” section.

Given its central role in immune and inflammatory responses, NF-κB signaling involves extensive crosstalk with immune pathways mediated by TLRs, RLRs, and STING-dependent pathways.

### Crosstalk between the NF-κB and TLR signaling pathways: ubiquitin-dependent regulation

These interactions enable NF-κB to integrate signals from diverse immune stimuli, thereby coordinating a broad spectrum of cellular responses to infection, inflammation, and other immune challenges. A key example of this interplay is observed in the TLR4/7/8/9 signaling pathways, where NF-κB activation is tightly regulated through ubiquitin-dependent mechanisms. In the TLR4 pathway, activation of the MyD88-dependent cascade increases TRAF6 activity, which catalyzes the formation of K63-linked PUCs. These chains serve as critical scaffolds for recruiting and activating the TAK1 complex and the IKK complex, ultimately driving NF-κB activation and the upregulation of proinflammatory gene expression (Fig. 3).^172,173^ Similarly, in the TLR7/8/9 pathways, K63-linked PUCs facilitate the assembly of TRAF3, IKKα, and interferon regulatory factor 7 (IRF7), promoting the production of IFNs. NF-κB signaling is further modulated in the TRIF-dependent TLR pathway, where K63-linked polyUb of receptor-interacting Ser/threonine-protein kinase 1 (RIPK1), which is mediated by the E3 Ub ligase Peli1, plays a crucial role.^174^ This modification enhances NF-κB activation, thereby regulating the transcription of immune and inflammatory response genes (Fig. 3). Additionally, MARCH5, a mitochondria-localized E3 Ub ligase, amplifies TLR signaling by catalyzing the K63-linked polyUb of TRAF family member-associated NF-κB activator (TANK).^175^ These mechanisms highlight the central role of K63-linked polyUb in bridging TLR signaling with NF-κB activation, enabling the coordination of both inflammatory and antiviral responses. By integrating signals from TLR pathways, NF-κB acts as a master regulator of innate immunity, fine-tuning cellular responses to diverse immune challenges.

Ubiquitination functions as a crucial safeguard against tumorigenesis by preventing excessive activation of NF-κB at multiple regulatory checkpoints. Key E3 Ub ligases, such as neuregulin receptor degradation protein 1 (Nrdp1), tripartite motif-containing 38 (Trim38), WW domain-containing E3 Ub-protein ligase 2 (WWP2), and PDZ and LIM domain 2 (PDLIM2), play essential roles in this regulatory network. Nrdp1 targets MyD88,^176^ Trim38 ubiquitinates TRAF6,^177^ WWP2 modifies TRIF,^178^ and PDLIM2 acts on p65,^179^ with each catalyzing K48-linked polyUb of their respective substrates. This modification marks target proteins for proteasomal degradation, effectively dampening NF-κB signaling and ensuring the maintenance of cellular homeostasis.

### Crosstalk between the NF-κB and RLR signaling pathways: ubiquitin-dependent regulation

The NF-κB signaling pathway plays a key role in mediating immune responses to viral infections, particularly through its crosstalk with the RLR family, which includes laboratory of genetics and physiology 2 (LGP2), melanoma differentiation-associated protein 5 (MDA5), and RIG-I.^180^ Upon recognition of viral RNA, MDA5 and RIG-I initiate the mitochondrial antiviral signaling (MAVS)-TANK-binding kinase 1 (TBK1) -IRF3 signaling cascade, which not only drives the expression of antiviral genes but also intersects with NF-κB signaling to amplify immune responses.^180^ Many E3 ligases, including Trim25,^181^ LUBAC,^182^ and RNF135,^183^ also regulate the RLR immune signaling pathway. For example, Trim25 catalyzes the K63-linked polyUb of RIG-I, facilitating the recruitment of MAVS and the formation of the MAVS signalosome, which activates downstream signaling, including NF-κB.^180^ On the other hand, LUBAC negatively regulates RIG-I activation by inhibiting Trim25 binding to RIG-I or promoting Trim25 degradation, thereby fine-tuning the immune response.^182^ Similarly, RNF135 (Riplet) enhances RIG-I signaling through K63-linked polyUb, further linking RLR activation to NF-κB signaling.^184^ Moreover, the E3 ligase MIB1/2 regulates TBK1 via K63-linked polyUb, a key step in activating both the IRF3 and NF-κB pathways.^185^ Furthermore, K27-linked polyUb of NEMO, which is mediated by the Shigella effector IpaH9.8 or Trim23, promotes the activation of the IKK and TBK1 complexes, which are essential for NF-κB signaling.^186,187^ These intricate ubiquitination events highlight the central role of NF-κB in integrating signals from the RLR pathway, ensuring a robust and coordinated antiviral immune response (Fig. 3). When simulated with an RNA virus, NF-κB signaling is intricately regulated through ubiquitination events that modulate the RLR pathway. For example, Siglec-G recruits the E3 ligase c-Cbl, which catalyzes the K48-linked polyUb of RIG-I, leading to its proteasomal degradation.^188^ This process attenuates RIG-I-mediated activation of downstream signaling, including NF-κB, thereby fine-tuning the antiviral response. MAVS, a central adapter in the RLR pathway, is also subject to ubiquitination-mediated regulation. The poly(rC)-binding protein PCBP2 recruits the E3 ligase AIP4 (ITCH), which, in turn, results in the catalysis of the K48-linked polyUb of MAVS, promoting its degradation and suppressing the MAVS-dependent activation of NF-κB and IFN responses.^189^ Furthermore, downstream, multiple E3 ligases regulate the stability of MAVS signaling components to modulate NF-κB activity. For example, NLRP4 recruits the E3 ligase DTX4 to promote the ubiquitination and degradation of TBK1, a key kinase that activates both IRF3 and NF-κB.^190^ Similarly, Triad3A catalyzes the K48-linked polyUb of TRAF3,^191^ whereas RNF5 mediates the K48-linked ubiquitination and degradation of MAVS itself.^192^ These ubiquitination events collectively serve as critical checkpoints to prevent excessive NF-κB activation, ensuring a balanced immune response to viral infections (Fig. 3).

### Crosstalk between the NF-κB- and STING-dependent signaling pathways: ubiquitin-dependent regulation

NF-κB signaling is intricately linked to the STING pathway, a key innate immune-sensing mechanism for tumor surveillance.^193^ STING, an ER-resident transmembrane adapter protein, plays a pivotal role in detecting cytoplasmic DNA, which is often a hallmark of cellular stress or infection.^194^ Cytoplasmic DNA is recognized by cyclic GMP-adenosine 3’,5’-monophosphate (AMP) synthase (cGAS), which activates the STING-TBK1-IRF3 signaling axis, leading to the production of IFNs.^195^ Ubiquitination plays a pivotal role in regulating the activation, stability, and function of cGAS, a process orchestrated by multiple E3 Ub ligases. RNF185, the first identified E3 Ub ligase for cGAS, primarily mediates K27-linked polyUb, thereby increasing cGAS enzymatic activity.^196^ TRIM56 catalyzes monoubiquitination of cGAS at lysine 335 (K335), enhancing its dimerization, DNA-binding affinity, and cGAMP production, which are critical for cytoplasmic DNA sensing and the induction of IFN-α/β production to combat DNA viral infections.^197^ Additionally, TRIM14, which is induced by type I IFN, stabilizes cGAS by recruiting USP14 to cleave Ub chains at lysine 414 (K414).^198^ Furthermore, USP15 activates cGAS through two distinct mechanisms in response to DNA: facilitating cGAS deubiquitination and enhancing its phase separation, a mechanism that diverges from conventional deubiquitination pathways such as those mediated by USP14.^199^ Furthermore, the deubiquitinating enzyme USP27X interacts with cGAS and removes K48-linked PUCs, thereby stabilizing cGAS and ensuring its functional integrity.^200^ These findings underscore the intricate and multifaceted regulation of cGAS by ubiquitination and deubiquitination, highlighting their critical roles in innate immune responses.

Importantly, this pathway also intersects with NF-κB signaling, as STING activation can promote NF-κB-dependent inflammatory responses.^201^ Upon sensing cytoplasmic DNA, STING dimerizes and translocates from the ER to perinuclear microsomes, where it recruits and activates TBK1.^39^ The formation of the STING-TBK1 complex at these perinuclear sites is critical for TBK1 activation, which in turn phosphorylates and activates both IRF3 and NF-κB.^39^ This spatially coordinated mechanism underscores the central role of NF-κB in amplifying innate immune signaling, bridging DNA sensing with inflammatory and antiviral responses.

**Fig. 4:**
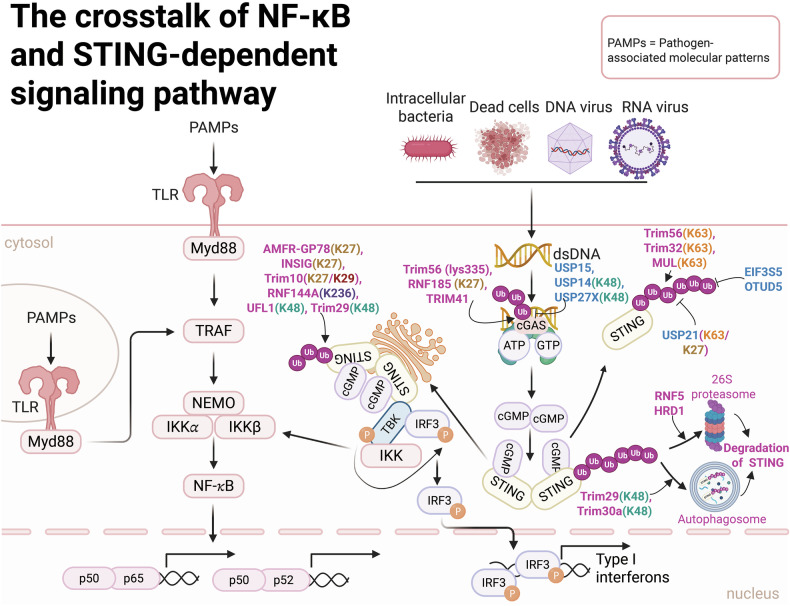
Ubiquitination and deubiquitination of the stimulator of interferon (IFN) gene (STING)-dependent signaling pathway and its crosstalk with the NF-κB signaling pathway. This figure illustrates the dynamic regulation of the stimulator of interferon genes (STING)-dependent signaling pathway through ubiquitination and deubiquitination and its intricate crosstalk with the NF-κB pathway, highlighting a central node in the host innate immune response against cytosolic DNA from pathogens or damaged host cells. The elements were obtained from BioRender.com

Emerging research has revealed that STING undergoes diverse polyUb modifications, including K48-, K63-, K11-, K29-, and K27-linked PUCs, each of which plays a pivotal role in orchestrating the innate immune response to a broad spectrum of threats, such as intracellular bacteria, dead cells, and viral RNA or DNA infections.^39,202^ The unique structural configurations of these PUCs not only increase the functional versatility of STING but also precisely modulate the amplitude and duration of its activity in driving the transcriptional activation of type I IFN genes (Fig. 4).^202^ The subcellular localization and functional dynamics of STING are intricately regulated by a network of E3 Ub ligases,^202^ which mediate its ubiquitination, thereby regulating its stability, intracellular trafficking, and signaling efficacy in response to pathogen-associated nucleic acids. Under conditions of exogenous DNA stimulation, TRIM56 catalyzes the K63-linked polyUb of STING, facilitating its dimerization and subsequent recruitment to TBK1, thereby driving the production of IFN-1b.^203^ Similarly, RNF115, TRIM32, and mitochondrial E3 Ub ligase 1 (MUL1) promote the K63-linked polyUb of STING, which enhances its efficient trafficking and the activation of downstream signaling pathways.^204^ In contrast, USP21, a key deubiquitinating enzyme, negatively regulates STING activity by hydrolyzing K63- and K27-linked PUCs.^205^ This deubiquitination process suppresses the synthesis of IFNs in response to DNA viral infection, highlighting a counterregulatory mechanism that fine-tunes STING-mediated innate immune signaling.^205^

In addition to its role in signal transduction, K48-linked polyUb serves as a key regulatory mechanism in STING signaling by targeting the protein for proteasomal degradation. Upon stimulation by RNA or DNA, RNF5 catalyzes the K48-linked polyUb of STING at lysine residues 150 and 48.^206,207^ This modification functions as a proteolytic signal, directing STING for degradation via the 26S proteasome pathway.^206,207^ In contrast, UFL1, the only known E3 Ub ligase for UFM1, antagonizes this process by competitively binding to TRIM29 and STING.^208,209^ This interaction diminishes K48-linked ubiquitination at lysine residues 338, 347, and 370, thereby preventing STING degradation, maintaining its protein stability, and amplifying the antiviral immune response.^208,209^ On the other hand, TRIM30a and RNF5 promote the K48-linked polyUb of STING, leading to its proteasomal degradation and subsequent inhibition of the DNA-triggered signaling cascade.^210,211^ Moreover, DUBs, including eukaryotic translation initiation factor 3 subunit 5 (EIF3S5) and OTUD5, play critical roles in regulating STING stability by removing K48-linked PUCs.^212,213^ EIF3S5 is recruited by inactive rhomboid protein 2 (iRhom2) and directly interacts with STING,^213^ while OTUD5 specifically cleaves K48-linked PUCs at the Lys347 site of STING.^212^ By counteracting K48-linked ubiquitination, both EIF3S5 and OTUD5 protect STING from proteasomal degradation, thereby maintaining its stability and ensuring robust downstream innate immune responses.^212,213^

The E3 ligase RNF26 catalyzes the K11-linked polyUb of STING.^214^ During the early stages of viral infection, RNF26-mediated K11 PUCs compete with K48-linked ubiquitination, thereby preventing RNF5-induced STING degradation and increasing type I IFN expression.^214^ However, in the later stages of infection, RNF26 suppresses type I IFN production by promoting lysosomal degradation of IRF3.^214^ STING degradation is also regulated through a microautophagy mechanism dependent on the endosomal sorting and transport complex (ESCRT).^215^ During this process, STING undergoes K63-linked ubiquitination at lysine 288, a modification critical for its degradation to prevent excessive immune activation.^215^

Additionally, 3-hydroxy-3-methylglutaryl reductase degradation protein 1 (HRD1), another E3 Ub ligase, primarily mediates K27-linked ubiquitination of STING, facilitating the degradation of ER-resident STING proteins and thereby attenuating STING-mediated immune responses.^216^ In addition, TRIM10 mediates the ubiquitination of STING at lysine residues 289 and 370 through K27- and K29-linked PUCs, respectively.^217^ This modification facilitates the translocation of STING from the ER to the Golgi apparatus, promotes its aggregation within the Golgi, and enhances the recruitment of the downstream kinase TBK1.^217^ The E3 Ub ligase complex, comprising AMFR-GP78 and INSIG1 and localized within the ER, facilitates the K27-linked polyUb of STING.^218^ This modification not only recruits TBK1 but also initiates the production of IFN.^218^ Additionally, RNF144A-mediated K6-linked polyUb of STING at K236 plays a critical role in STING translocation, thereby regulating STING-dependent antiviral responses.^219^ These findings reveal a multilayered regulatory network of ubiquitination that finely tunes STING activity and ensures balanced immune responses.

### The MAPK signaling pathway and its regulation by ubiquitination and deubiquitination

**Fig. 5:**
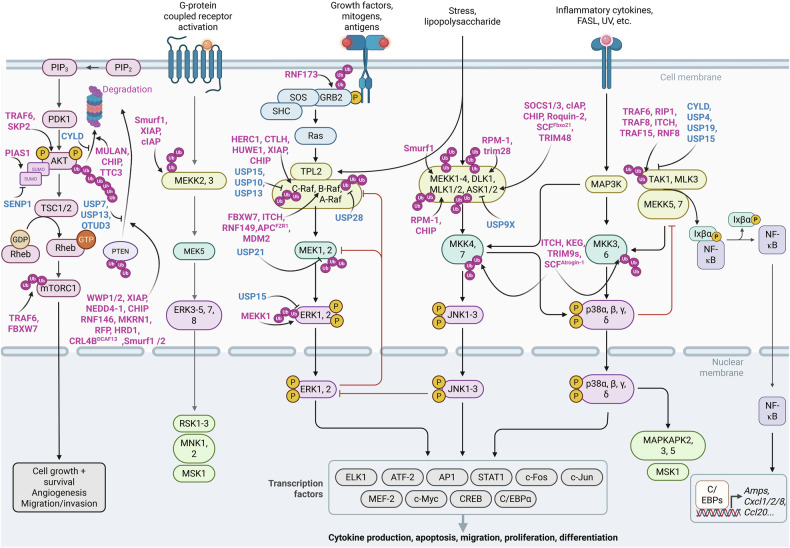
Ubiquitination and deubiquitination in the mitogen-activated protein kinase (MAPK) and phosphoinositide 3-kinase-Ak strain transformation (PI3K-AKT) signaling pathways and their crosstalk with the NF-κB signaling pathway. This figure illustrates the crucial roles of ubiquitination and deubiquitination in the regulation of the MAPK and PI3K-AKT signaling pathways and their functional crosstalk with the NF-κB signaling cascade. These pathways collectively govern key cellular processes, including cell proliferation, differentiation, and survival, and are tightly regulated by posttranslational modifications such as ubiquitination. The elements were obtained from BioRender.com

The MAPK pathway represents the core signaling cascade downstream of RAS. Upon activation, RAS proteins initiate a sophisticated network of downstream MAPK signaling pathways, which are essential for mediating cellular responses to extracellular signals, including cytokines, cytokine receptors, hormones, protein kinases, and TFs.^33^ This pathway regulates cell proliferation, survival, and differentiation, underscoring its central role in cellular physiology and disease (Fig. 5).^33^

In the MAPK signaling cascade, RNF173 functions as a negative regulator of the RAF/MEK/ERK pathway by ubiquitinating and targeting GRB2 for degradation, thereby attenuating downstream signaling.^220^ Additionally, E3 Ub ligases and DUBs targeting C-RAF and B-RAF have been identified as key regulators within the RAF family. Several E3 Ub ligases, including FBXW7, ITCH, RNF149, MDM2, RNF43, and the APC/C complex activator APC^FZR1^, have been identified as key regulators of B-RAF ubiquitination. These ligases critically modulate B-RAF stability, kinase activity, and downstream signaling, thereby exerting profound effects on the ERK1/2 pathway and its associated cellular processes. Among these, RNF149 was the first E3 ligase discovered to target the B-RAF.^221^ APC^FZR1^ specifically interacts with B-RAF and A-RAF but induces proteasomal degradation exclusively of B-RAF, highlighting its substrate specificity.^222^ ITCH, another E3 ligase, regulates B-RAF kinase activity and promotes K27-linked polyUb of B-RAF, particularly in MM.^223^ RNF43 mediates the ubiquitination of B-RAF at lysine 499 (K499), targeting it for proteasome-dependent degradation.^224^ Additionally, although not a direct DUB for B-RAF, USP28-mediated deubiquitination of FBXW7 enhances B-RAF ubiquitination and subsequent degradation by the SCF Ub ligase complex.^225^ C-RAF is regulated by ubiquitination through several E3 ligases, including the C-terminus of HSC70-interacting protein (CHIP), HERC1, the C-terminal to LisH (CTLH) complex, and HUWE1, UBA, and HECT. Among these, HERC1 specifically targets C-RAF and not only influences the MAPK signaling pathway but also modulates the p38 signaling cascade.^40^ HUWE1 regulates RAF activity by ubiquitinating C-RAF. Additionally, HUWE1 ubiquitinates the scaffold protein SHOC2, which is essential for mediating C-RAF ubiquitination and modulating ERK1/2 activity.^226^ In contrast to other E3 ligases, CHIP and XIAP control C-RAF stability independently of their E3 ligase activity, promoting C-RAF ubiquitination and degradation through the Hsp-90-mediated quality control system.^227^ On the deubiquitination side, USP15 binds to C-RAF and protects it from 26S proteasome-mediated degradation.^228^ USP13, the most recently identified DUB for C-RAF, directly interacts with and stabilizes C-RAF.^229^ Interestingly, USP15 does not regulate C-RAF stability through its DUB activity but instead modulates C-RAF mRNA stability and translational competence.^228^

The E3 ligases regulating MEK1/2, key MAPKKs in the ERK1/2 signaling cascade, remain largely unidentified. Currently, USP21 is the only known DUB controlling MEK1/2. USP21 stabilizes MEK2 by removing K48-linked PUCs, thereby activating the ERK signaling cascade and promoting hepatocellular carcinoma (HCC) progression.^230^

The ubiquitination of ERK1/2 is regulated by USP15 and MEKK1. MEKK1, an upstream activator of the MKK4-JNK signaling pathway, contains a plant homeodomain (PHD) at its N-terminus. This PHD domain features a RING finger-like structure that functions as an E3 ligase, enabling MEKK1 to promote the polyUb of ERK1/2.^231^ In contrast, USP15, a recently identified DUB for ERK2, removes Ub chains from ERK2 without affecting its protein stability. Additionally, USP15 enhances ERK1/2 phosphorylation and activates the TGF-β/SMAD2 signaling pathway, revealing its dual role in modulating ERK1/2 activity and crosstalk with TGF-β signaling.^232^

In the ERK5 signaling cascade, XIAP, Smurf1, and cIAP are the only known E3 ligases that regulate MEKK2/3. Smurf1 specifically targets MEKK2 and contributes to the activation of the JNK signaling cascade.^233^ cIAP and XIAP, previously described as regulators of ERK1/2 activity through C-RAF stabilization, are linked to increased ERK5 activity in IAP-deficient cells. Furthermore, XIAP competitively disrupts the interaction between MEKK2/3 and MEK5 by directly binding to MEKK2.^234^ This binding promotes K63-linked polyUb of MEKK2/3, maintaining their activity and driving ERK5 activation.^234^ These findings underscore the intricate regulatory mechanisms of the ERK5 pathway and its crosstalk with other signaling cascades.

The p38 and JNK1/2/3 signaling cascades share several upstream regulators, including MAPKKKs (such as MEKK1-4, TAK1, DLK1, and MLK1-4) and MAPKKs (such as MKK4/5/6), which play critical roles in mediating cellular responses to stress and inflammation.^40^ These pathways are tightly regulated by ubiquitination. For example, in inflammatory responses, the E3 ligase Smurf1, in conjunction with NDR2 and STK38, ubiquitinates MEKK2 via K48-linked polyUb, targeting it for proteasomal degradation.^235^ Similarly, DLK1, a MAPKKK involved in both p38 and JNK signaling, is regulated by the E3 ligases TRIM28 and RPM-1.^236,237^ RPM-1 also regulates MLK1, another MAPKKK within these cascades, highlighting the conserved role of ubiquitination in modulating MAPK signaling.^238^

The MKK family of MAPKKs, which are essential for activating both the JNK and p38 pathways, serves as a central node for crosstalk between these signaling cascades. Specific E3 ligases have been identified for MKK6, MKK5, and MKK4. ITCH regulates MKK4,^239^ Keep ON GOING (KEG) targets MKK4/5,^240^ and the short isoform of TRIM9 and the SCF^Atrogin-1^ complex regulate MKK6.^241^ These Ub ligases fine-tune the activity and stability of MKKs, thereby modulating downstream signaling outputs.

### The PI3K-AKT signaling pathway and its regulation by ubiquitination and deubiquitination

The RAS–PI3K–AKT–mTOR pathway represents another core signaling cascade downstream of RAS. This pathway also regulates critical processes such as cell survival, proliferation, and differentiation^33^ (Fig. 5).

Growth factors are sensed primarily through the PTEN-AKT signaling pathway, a critical axis that regulates cellular growth, survival, and metabolism in response to extracellular stimuli. PTEN plays a central role in suppressing tumorigenesis by inhibiting the PI3K signaling pathway. The regulation of PTEN is tightly controlled through ubiquitination and deubiquitination. A diverse array of E3 ubiquitin ligases, including WWP1/2, XIAP, NEDD4-1, CHIP, RNF146, MKRN1, Ret finger protein (RFP), HRD1, CRL4^DCAF13^ (Cullin-RING E3 Ub ligase), and Smurf1/2, have been identified as specific regulators of PTEN ubiquitination. In addition to NEDD4-1, the E3 ligase WWP1 also ubiquitinates PTEN, impairing its dimerization, membrane association, and tumor-suppressive functions.^242^ Pharmacological inhibition of WWP1 restores PTEN activity, effectively suppressing MYC-driven tumorigenesis, highlighting WWP1 as a potential therapeutic target in MYC-dependent cancers.^243^ Furthermore, WWP2 has been shown to ubiquitinate PTEN, modulating its stability and thereby regulating apoptosis through PTEN degradation.^244^ In addition to the HECT family, other E3 ligases, including XIAP, also target PTEN for ubiquitination and degradation.^245^ NF146, an E3 Ub ligase, recognizes ribosylated PTEN and targets it for ubiquitination and subsequent degradation. This process specifically involves the modification of lysine residues Lys342, Lys344, and Lys349 on PTEN, marking it for proteasomal degradation.^246^ Recent studies have expanded the repertoire of E3 ligases that regulate PTEN stability. For example, oncogenic MKRN1, which is upregulated through the EGF-AKT signaling pathway, promotes PTEN degradation via ubiquitination at lysine residues Lys48 and Lys289, targeting PTEN for proteasomal degradation.^247^ Additionally, RFP, a RING-type E3 Ub ligase, has been identified as a novel regulator of PTEN, which primarily mediates ubiquitination at Lys27, along with other lysine residues, to modulate PTEN function.^248^ RFP-mediated polyUb specifically inhibits the catalytic activity of PTEN without affecting its stability or subcellular localization, thereby relieving the negative regulation of AKT signaling by PTEN.^248^

In addition to the complexity of PTEN regulation, HRD1 has emerged as another E3 ligase that ubiquitinates and destabilizes PTEN.^249^ Similarly, the CRL4^DCAF13^ E3 ligase complex has been implicated in the proteasomal degradation of PTEN, further highlighting the diverse mechanisms by which PTEN stability is controlled.^250^ In glioblastoma (GB), upregulation of Smurf1 induces ubiquitination-mediated degradation of PTEN, leading to sustained aberrant activation of the PI3K/AKT/mTOR pathway, a hallmark of tumor progression.^251^ The reversibility of ubiquitination has drawn significant attention to the role of DUBs in regulating PTEN. USP7, a prominent DUB, directly deubiquitinates PTEN, modulating its subcellular localization rather than affecting its protein stability.^252^ Recent studies have shown that USP7 deletion or mutation causes Hao-Fountain syndrome (HAFOUS),^253^ which is described in the “Developmental abnormalities” section. Additionally, USP13 and OTUD3 have been shown to interact with PTEN and remove its PUCs, thereby stabilizing PTEN and preventing its degradation.^254,255^ This stabilization of PTEN inhibits the activation of the AKT signaling pathway, ultimately suppressing the growth of tumors. These results underscore the critical balance between ubiquitination and deubiquitination in controlling the function of PTEN and its downstream oncogenic signaling pathways.

The activity, stability, and downstream signaling outputs of AKT, a pivotal kinase in cellular signaling pathways, are intricately regulated by ubiquitination. A diverse array of E3 Ub ligases, including MULAN, NEDD4, CHIP, tetratricopeptide repeat domain 3 (TTC3), SKP2, and TRAF6, are critical regulators of AKT ubiquitination. TRAF6, for example, functions as a direct E3 ligase for AKT upon stimulation with IGF-1, catalyzing K63-linked polyUb.^256^ This modification is essential for AKT membrane recruitment, phosphorylation, and subsequent activation. Cancer-associated AKT mutations frequently exhibit increased ubiquitination, further highlighting the importance of this PTM in oncogenic signaling.

SKP2, another E3 ligase, mediates AKT ubiquitination downstream of ErbB receptor activation. In models of breast cancer metastasis, SKP2 deficiency attenuates AKT activation, underscoring its role in tumor progression.^257^ Unlike K63-linked ubiquitination, which primarily regulates AKT activation, K48-linked ubiquitination governs AKT stability, targeting it for proteasomal degradation. Additional E3 ligases, including CHIP, MULAN, and TTC3, have also been implicated in AKT ubiquitination, fine-tuning its signaling dynamics through degradation.^258–260^

Conversely, DUBs serve as “erasers” that counteract AKT ubiquitination, influencing its degradation, subcellular localization, activation, and protein‒protein interactions. As a well-characterized tumor suppressor, cylindromatosis (CYLD) directly interacts with AKT and removes K63-linked PUCs in response to growth factor stimulation.^261^ This deubiquitination promotes K48-linked polyUb, which is mediated by TTC3, leading to AKT degradation. When the function of CYLD is lost, it results in accelerated tumorigenesis and confers cisplatin resistance in oral squamous cell carcinoma and melanoma, underscoring its critical role in regulating AKT signaling.^262,263^

In addition to ubiquitination, other PTMs play pivotal roles in regulating AKT activity. Lysine 276, located within a SUMOylation consensus motif, is vital for the activation of AKT. Mutation of this residue (K276R) diminishes AKT SUMOylation, impairing its function.^264^ Furthermore, the oncogenic AKT E17K mutation drives cell proliferation, migration, and tumorigenesis, illustrating the complexity of the regulatory networks that govern AKT activity.^264^

Undoubtedly, mTOR plays a central role in the amino acid-induced activation of the AKT-mTOR signaling pathway. The lysosomal localization of mTORC1, facilitated by RagA deubiquitination—its reversible process regulated by the E3 ligases RNF152 and SKP2—is a critical prerequisite for its activation.^124,265^ In addition, the E3 Ub ligase TRAF6 has been shown to regulate mTOR translocation to the lysosome in response to amino acid stimulation. TRAF6 achieves this by catalyzing the K63-linked polyUb of mTOR, which functions within the p62-TRAF6 heterodimer complex.^266^ This mechanism not only activates mTORC1 but also modulates autophagy and cancer cell proliferation, underscoring the multifaceted role of TRAF6 in cellular homeostasis and oncogenesis. In addition to K63-linked ubiquitination, other polyubiquitin linkages also regulate mTOR. For example, K48-linked ubiquitination is implicated in controlling mTOR stability. The E3 ligase FBXW7 directly interacts with mTOR and mediates its K48-linked polyUb, targeting it for proteasomal degradation.^267^ These findings highlight the pivotal role of ubiquitination in the mTORC1 pathway, demonstrating that distinct Ub linkages—K63 for activation and K48 for degradation—dictate divergent functional outcomes.

### Crosstalk between MAPK, PI3K-AKT and other signaling pathways: ubiquitin-dependent regulation

Ubiquitination plays a pivotal role in integrating and modulating signaling pathways, including those involving MAPK, PI3K-AKT-mTORC, and NF-κB. For example, the ubiquitination of MEKK2/3 is intricately linked to biphasic NF-κB activation, underscoring its role in coordinating cellular responses across multiple signaling networks.^268^ This regulatory mechanism highlights how ubiquitination serves as a critical node for crosstalk between pathways, fine-tuning cellular outcomes in response to diverse stimuli.

Recent studies have revealed that SMAD-specific E3 ubiquitin-protein ligase 2 (Smurf2), an E3 Ub ligase, polyubiquitinates endosome-associated PTEN, targeting it for degradation.^269^ This process not only destabilizes PTEN but also promotes the activation of the noncanonical NF‒κB pathway, linking PTEN loss to alternative oncogenic signaling cascades. These findings illustrate how the ubiquitination of key tumor suppressors can redirect signaling dynamics, contributing to cancer progression.

TAK1, a MAPKKK essential for both JNK/p38 and NF-κB signaling, is one of the most extensively studied components of the MAPK pathway because of its regulation by K63-linked polyUb, which is crucial for its activation. TRAF6, a central E3 ligase, directly binds TAK1 and catalyzes its K63-linked polyUb, facilitating signal transduction.^270^ Inflammatory cytokines further modulate TAK1 activity: TNFα induces the cytoplasmic translocation of Trim8, while IL-1β enhances the K63-linked polyUb of TAK1, driving JNK and NF-κB activation.^271,272^ In contrast, Trim15 acts as a negative regulator, inhibiting NF-κB activation by reducing the K63-linked polyUb of TAK1 and blocking Trim8 translocation.^273^ Additionally, RNF8, which is activated by Tax, assembles K63-linked PUCs in concert with the E2 enzyme Ubc13: UBL1 activating enzyme (UEV1A)/Uev2, indirectly promoting NF-κB activation.^274^

The regulation of TAK1 is further fine-tuned by DUBs, which counteract ubiquitination to maintain signaling homeostasis. For example, the CYLD and ITCH complex sequentially cleaves K63-linked PUCs and promotes the K48-linked polyUb of TAK1, targeting it for proteasomal degradation.^275^ Other DUBs, including USP4, USP15, USP18, and CYLD, also play critical roles in regulating TAK1 activity.^276–278^ This dynamic interplay between ubiquitination and deubiquitination underscores the complexity of signaling regulation and highlights the importance of PTMs in controlling cellular responses.

### The Wnt/β-catenin signaling pathway and its regulation by ubiquitination and deubiquitination

In the canonical Wnt signaling pathway, key components include low-density lipoprotein receptor-related proteins 5 and 6 (LRP5/6), the Frizzled (Fz) receptor, Disheveled (Dvl), β-catenin, glycogen synthase kinase 3 (GSK3), casein kinase 1 (CK1), Axin, adenomatous polyposis coli (APC), and the DNA-bound T-cell factor/lymphoid enhancer factor (TCF/LEF).^279^ In the absence of Wnt ligands, β-catenin is phosphorylated by a destruction complex composed of APC, GSK3, CK1, and Axin. This phosphorylation targets β-catenin for ubiquitination by the βTRCP E3 ligase, leading to its proteasomal degradation. Consequently, the nuclear translocation of β-catenin is inhibited, preventing the activation of Wnt target gene expression. Upon Wnt ligand binding, the destruction complex is recruited to the activated receptor complex, which prevents β-catenin phosphorylation and ubiquitination. This stabilization allows β-catenin to accumulate and translocate to the nucleus, where it interacts with TCF/LEF TFs to initiate the expression of Wnt target genes.^280^

**Fig. 6:**
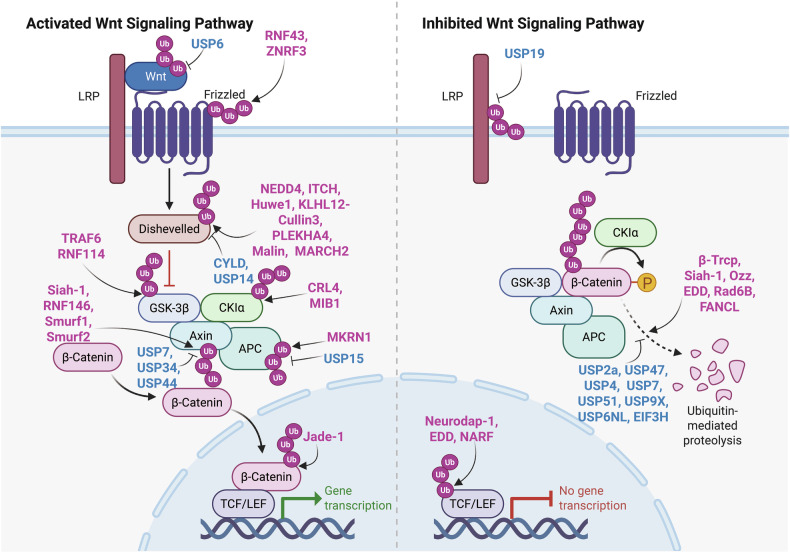
Ubiquitination and deubiquitination in the Wnt/β-catenin signaling pathway. This figure illustrates the dynamic interplay between ubiquitination and deubiquitination in the regulation of the Wnt/β-catenin signaling pathway, emphasizing the importance of these posttranslational modifications in maintaining the balance between Wnt activation and deactivation, which is critical for normal cellular function and disease progression. The elements were obtained from BioRender.com

Among the myriad of PTMs, ubiquitination and deubiquitination are pivotal regulators of most Wnt signaling components (Fig. 6). Ubiquitination regulates the Wnt/β-catenin signaling pathway through two key mechanisms.^281^ First, Wnt signaling phosphorylates β-catenin, triggering its ubiquitination and subsequent proteasomal degradation. As a result, low β-catenin levels in the nucleus prevent the activation of Wnt target genes by transcriptional repressors.^281^ Second, the Ub ligases RNF43 and ZNRF3 facilitate the ubiquitination of Wnt receptors, thereby reducing their availability on the cell surface.^281^

Ubiquitination, orchestrated by E3 ligases, conjugates Ub molecules to target proteins, often leading to the proteasomal degradation of critical Wnt signaling elements such as Dvl, GSK3, Axin, and β-catenin. In stark contrast, deubiquitination, which is mediated by DUBs, detaches Ub moieties, thereby modulating the stability of these signaling factors. Within the Wnt signaling cascade, the UPS is indispensable for regulating the stability of β-catenin, a central signaling molecule that drives the expression of Wnt target genes.^279^ For example, βTRCP serves as a key regulator that targets β-catenin for ubiquitination.^282^ Siah-1 has been demonstrated to directly polyubiquitinate β-catenin, underscoring its role as an E3 ligase in regulating β-catenin stability. In contrast, the p53-induced SCF-like complex (SCF(TBL1)) has been shown to counteract this process by protecting β-catenin from Siah-1-mediated ubiquitination in vitro and shielding it from proteasomal degradation in cellular environments.^283^ While Siah-1 does not influence the degradation of cytosolic β-catenin or its nuclear translocation, it specifically enhances the degradation of membrane-bound β-catenin in *Helicobacter pylori*-infected gastric cancer cells.^284^ The Ozz-E3 ligase facilitates the ubiquitination of sarcoplasmic-associated β-catenin, whereas Jade-1 directs the ubiquitination-mediated degradation of nuclear β-catenin.^285^ In contrast, ubiquitination mediated by EDD/Rad6B/FANCL enhances the stability of β-catenin. Specifically, EDD catalyzes the formation of K11- and K29-linked PUCs, whereas Rad6B facilitates the attachment of K63-linked PUCs.^285^ These diverse ubiquitination mechanisms highlight the intricate regulation of β-catenin stability and function across different cellular compartments. The deubiquitination of β-catenin is a finely regulated process, with several DUBs playing pivotal roles in modulating its stability, localization, and transcriptional activity. A notable example of β-catenin regulation is its interaction with USP4, a specific DUB for β-catenin that modulates its expression and fine-tunes β-catenin-mediated transcriptional programs. The catalytic domain at the C-terminus of USP4 facilitates its binding to β-catenin, driving the nuclear translocation of β-catenin and influencing Wnt signaling outcomes.^286^ The interaction of USP51 with β-catenin reduces its ubiquitination level.^280^ Additionally, USP47 prevents β-catenin ubiquitination. Intriguingly, USP47 can deubiquitinate itself, while βTRCP promotes its ubiquitination through interaction with an atypical motif within USP47.^287^ Furthermore, EIF3H interacts with β-catenin, deubiquitinates it, and stabilizes it by acting as a DUB. Mechanistically, EIF3H removes K48-linked Ub chains from β-catenin by binding to its N-terminal region.^288^ USP7, a versatile regulator of the Wnt signaling pathway, plays dual roles in modulating β-catenin activity. On the one hand, it suppresses Wnt/β-catenin signaling by stabilizing Axin, a core component of the β-catenin destruction complex, thereby reducing β-catenin levels and attenuating the expression of its downstream targets.^289^ On the other hand, USP7 can also act as a positive regulator of β-catenin stability. For example, ZMIZ2 recruits USP7, which deubiquitinates and stabilizes β-catenin, promoting its accumulation and contributing to colorectal tumorigenesis.^290^ Furthermore, USP9X enhances Wnt signaling by deubiquitinating BCL9, a coactivator of β-catenin. This deubiquitination promotes the assembly of the β-catenin-BCL9-PYGO complex, amplifying the transcriptional activation of Wnt/β-catenin target genes.^291^ Together, these findings underscore the context-dependent and complex roles of DUBs in regulating β-catenin stability, localization, and function, highlighting their critical importance in fine-tuning Wnt signaling dynamics.

The transmembrane E3 Ub ligases RNF43 and ZNRF3 are pivotal regulators of Wnt receptor degradation, acting as tumor suppressors by modulating Wnt signaling. These ligases control the ubiquitination-dependent stability of the Fz receptor, and mutations in RNF43 or ZNRF3 lead to sustained β-catenin stability, resulting in constitutive activation of Wnt signaling. Additionally, RNF43 and ZNRF3 facilitate the interaction between the Wnt receptor and the signaling protein Dvl, further influencing pathway activity.^292^ In contrast, USP6 regulates the Wnt/β-catenin pathway through its deubiquitinating activity, directly interacting with Fz to modulate its levels.^293^ Similarly, USP19 stabilizes LRP6, a coreceptor in the Wnt pathway, thereby impacting downstream signaling events.^294,295^

The TCF family plays a pivotal role in nuclear processes by competitively interacting with β-catenin and the TLE family, directly influencing the expression of Wnt target genes. TCF functions as a key regulator, adopting distinct roles depending on the absence or presence of Wnt signaling. When Wnt signals are absent, TCF acts as a repressor, facilitating the binding of the TLE family and promoting the ubiquitination-mediated degradation of cytoplasmic β-catenin. Conversely, upon Wnt activation, β-catenin translocates to the nucleus, where it partners with TCF to activate the transcription of Wnt target genes.^296^ Adding complexity to this regulatory network, TCF/LEF1 is targeted for ubiquitination and subsequent degradation by the RING-type E3 ligase Neurodap1 (Pja2, Praja ring finger Ub ligase 2), which modulates TCF/LEF1 protein levels.^297^ Furthermore, EDD, an E3 ligase for β-catenin, orchestrates the ubiquitin-mediated degradation of the TCF/LEF family.^298^ Another critical player is the Nemo-like kinase-associated ring finger (NARF) protein, which collaborates with the E2 Ub ligase E2-25K to incorporate Ub into the regulatory machinery, a process essential for inhibiting axial axis formation in Xenopus embryos.^299^

NEDD4L promotes the polyUb of Dvl2, targeting it for proteasomal degradation. NEDD4L mediates ubiquitination through Lys6, Lys27, and Lys29 linkages rather than through conventional Lys48-linked ubiquitination.^300^ Similarly, ITCH facilitates the ubiquitin–proteasome degradation of Dvl proteins.^301^ Huwe1, another E3 ligase, interacts with and ubiquitinates the cytoplasmic Wnt component Dvl in a manner dependent on Wnt3a and CK1ε.^302^ Mass spectrometry analysis revealed that HUWE1 specifically promotes K63-linked, but not K48-linked, polyUb of Dvl.^302^ Rather than inducing degradation, Huwe1-mediated ubiquitination of the DIX domain inhibits Dvl multimerization, a crucial process for Dvl function.^302^ KLHL12, a member of the Kelch-like protein family, facilitates the ubiquitination of Dvl, leading to its proteasomal degradation.^303^ In contrast, PLEKHA4 functions by recruiting KLHL12, the adapter responsible for substrate recognition, into clusters at the plasma membrane. This sequestration inhibits the ability of KLHL12 to ubiquitinate Dvl, resulting in elevated Dvl levels and increased Wnt/β-catenin signaling in mammalian cells.^304^ Additionally, membrane-associated RING-CH2 (March2), a RING-type E3 Ub ligase, suppresses Wnt signaling by regulating Dvl turnover through ubiquitin-mediated lysosomal degradation.^305^ Malin promotes the attachment of both K48- and K63-linked Ub chains to Dvl2, triggering its degradation via two pathways: the proteasome and autophagy. This process underscores Malin’s role in regulating Dvl2 stability through distinct ubiquitin-dependent mechanisms.^306^ CYLD, a deubiquitinating enzyme, interacts with Dvl and modulates its K63-linked ubiquitination. In CYLD-deficient cells, increased ubiquitination of the DIX domain enhances Dvl signaling activity.^307^ By expanding the regulatory landscape further, USP14 plays a role in deubiquitinating Dvl. Inhibition of USP14 leads to increased polyUb levels in Dvl, significantly impairing downstream Wnt signaling.^308^ These findings highlight the complex, multifaceted regulation of Dvl through ubiquitination and deubiquitination, underscoring their critical roles in modulating Wnt signaling dynamics.

Furthermore, components of the β-catenin destruction complex, such as GSK3, are subject to ubiquitination. TRAF6 has been identified as a direct E3 Ub ligase for GSK3β, and its ubiquitination activity plays a pivotal role in poly I:C-induced cytokine production. This process is mediated by the TRIF-dependent assembly of the TLR3-associated signaling complex.^309^ In parallel, circFADS1 interacts with GSK3β and enhances its ubiquitination and degradation by recruiting the E3 Ub ligase RNF114, whereas EIF4A3 facilitates the biogenesis of circFADS1.^310^ circFADS1 has been strongly associated with resistance to lenvatinib.^310^ In a related pathway, CK1α is ubiquitinated and degraded by the CRL4 complex in a lenalidomide-dependent manner.^311^ FERMT1 promotes the proteasomal degradation of CK1α via the E3 ligase MIB1, thereby activating Wnt signaling.^312^ More recently, MKRN1 (RNF61) was identified as an E3 ligase for APC, promoting its ubiquitination and degradation through binding to the armadillo repeat domain of APC.^313^ The COP9 signalosome (CSN) promotes β-catenin degradation and stabilizes APC, with the latter effect mediated by CSN-associated USP15.^314^ Reduced USP15 expression leads to increased APC degradation, highlighting its role in APC stabilization.^314^ Axin, another core component of the destruction complex, is targeted by multiple E3 ligases, including Smurf2, Smurf1, RNF146, and Siah-1. Siah-1 mediates ubiquitination by binding to the VxP motif of Axin and functions as a positive regulator of Wnt signaling.^315^ RNF146 facilitates K48-linked ubiquitination of PARylated Axin, promoting its degradation.^315^ Smurf1 targets Axin for degradation via ubiquitination at K505, whereas Smurf2, while also ubiquitinating Axin, does not induce its degradation but instead disrupts its interaction with LRP6, acting as a negative regulator of the pathway.^315^ As previously noted, USP7 suppresses Wnt/β-catenin signaling by stabilizing Axin, thereby reducing the levels of β-catenin and its downstream transcriptional targets.^289^ USP34 similarly decreases Axin1 ubiquitination, although this effect is lost in its catalytically inactive mutant. Knockdown of USP34 results in reduced Axin1 protein expression and increased β-catenin accumulation.^316^ USP44 also regulates Axin1 protein stability without affecting its mRNA level; its interaction with Axin1 leads to decreased ubiquitination in a USP44 expression-dependent manner.^317^

Diverse E3 ligases and DUBs are intricately linked with Wnt signaling factors, modulating the expression of target genes such as Cyclin D1 and c-Myc, which are implicated in tumor growth. While significant progress has been made in identifying the specific E3 ligases and DUBs that target Wnt signaling components and the precise ubiquitination sites on these proteins, many aspects of the ubiquitination and deubiquitination mechanisms within the Wnt pathway remain unclear. Future research must focus on elucidating these mechanisms to fully understand how ubiquitination and deubiquitination regulate Wnt signaling. Achieving this balance will be crucial for developing effective therapeutic strategies targeting the Wnt signaling pathway in various contexts.

### The Hippo signaling pathway and its regulation by ubiquitination and deubiquitination

**Fig. 7:**
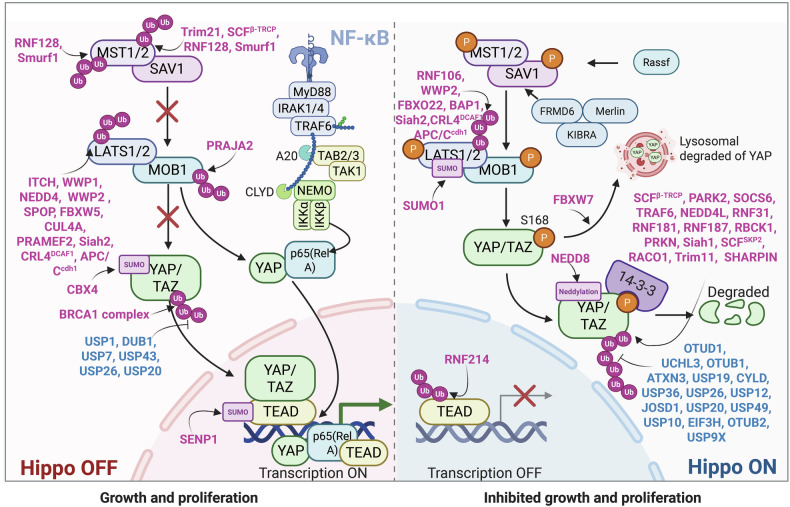
Ubiquitination and deubiquitination in the Hippo signaling pathway. This figure illustrates the regulatory roles of ubiquitination and deubiquitination in the Hippo signaling pathway, a critical pathway that controls organ size, tissue homeostasis, cell proliferation, and apoptosis. Dysregulation of this pathway is frequently associated with cancer, tissue overgrowth, and developmental defects. The elements were obtained from BioRender.com

The Hippo signaling pathway was first identified in *Drosophila melanogaster* through genetic mosaic screens, where it was found to regulate tissue growth by inhibiting excessive cell proliferation.^318^ The pathway is named after one of its core components, the protein kinase Hippo.^318^ In mammals, the Hippo kinases include mammalian STE20-like protein kinases 1 (MST1) and MST2, whereas the Warts kinases include the large tumor suppressors LATS1 and LATS2.^319^ Additionally, Salvador homolog 1 (SAV1) and MOB kinase activator 1 A/B (MOB1A/B) serve as homologs to Salvador and Mats, respectively.^320^ YAP and TAZ act as the primary downstream effectors of this pathway and are negatively regulated by Hippo signaling.^321^ In the canonical model, activation of the Hippo pathway occurs when MST1/2 phosphorylate SAV1, which in turn activates MOB1A/B and LATS1/2.^322^ This cascade leads to the phosphorylation of YAP and TAZ, promoting their binding to 14-3-3 proteins.^322^ This interaction results in their sequestration in the cytoplasm and subsequent proteasomal degradation via βTRCP.^322^ YAP and TAZ, which do not bind DNA directly, function by associating with TEA domain-containing TFs (TEADs), their primary transcriptional partners.^321,323^ When the Hippo pathway is inactive, LATS1/2 and MST1/2 are deactivated, leading to the hypophosphorylation of TAZ and YAP. This promotes their accumulation in the nucleus, where they interact with TEADs to drive the expression of target genes (Fig. 7).^324^

The expression of YAP, a central effector of the Hippo signaling pathway, is significantly upregulated in adult T-cell leukemia (ATL) cells through the action of the HTLV-1 Tax protein. YAP transcriptional activity is markedly elevated in ATL patients and HTLV-1-infected cells. Furthermore, Tax activates YAP via a mechanism involving the NF-κB/p65 pathway.^325^ This crosstalk between the NF-κB and Hippo pathways is mediated by p65, which disrupts the interaction between LATS1 and YAP (Fig. 7).^325^ This disruption suppresses YAP phosphorylation, inhibits the ubiquitination-dependent degradation of YAP, and promotes YAP nuclear accumulation, thereby increasing its transcriptional activity.

The Hippo pathway is regulated through various mechanisms, with ubiquitination playing a crucial role. The balance between ubiquitination and deubiquitination is essential for maintaining the normal functional output of the Hippo pathway. Multiple components of the Hippo pathway, such as Rassf, YAP/TAZ, MOB1, LATS1/2, MST1/2, and TEAD, are regulated by ubiquitination and deubiquitination. Furthermore, YAP, TEAD, and LATS1 can undergo sumoylation, and YAP is additionally modified by neddylation (Fig. 7).

In addition to the canonical E3 Ub ligase βTRCP, which regulates the Hippo pathway by ubiquitinating and degrading YAP, several other E3 ligases that modulate YAP stability and activity have been identified. For example, PARK2 interacts with cytosolic YAP, promoting its K48-linked ubiquitination at the K90 site, leading to proteasomal degradation.^326^ Similarly, SOCS6 facilitates the ubiquitination of YAP1.^327^ TRAF6, a downstream effector of IL-1β, induces YAP ubiquitination at K252, disrupting its interaction with angiomotin and enhancing YAP nuclear translocation.^328^ Interestingly, RNF31 associates with YAP, promoting its polyUb and degradation at the K76 site.^329^ RNF187 also promotes YAP degradation, likely through K48-dependent polyUb.^330^ RBCK1 enhances YAP stability by facilitating K48-linked polyUb at multiple lysine sites (K76, K204, and K321).^331^ Oncolytic Newcastle disease virus exacerbates ferroptosis in tumor cells by inducing ubiquitin-mediated degradation of YAP at Lys90 via the E3 Ub ligase parkin (PRKN).^332^ Furthermore, FRK phosphorylates YAP at Tyr391/407/444, recruiting the E3 ligase Siah-1 to catalyze YAP ubiquitination and degradation. Siah-1 is crucial for the destabilization of YAP initiated by FRK.^333^ SKP2 mediates the K63-linked polyUb of YAP, enhancing its nuclear trafficking and transcriptional activity in cancer cells.^334^ TRIM11 stabilizes YAP by inducing its monoubiquitination.^335^ In contrast, SHARPIN promotes YAP degradation via K48-dependent polyUb.^336^

With respect to the DUBs of YAP, OTUB2, when poly-SUMOylated at lysine 233, binds to and deubiquitinates YAP/TAZ, thereby activating them.^337^ UCH-L3 stabilizes YAP through its deubiquitination activity.^338^ OTUB1 interacts with YAP via its OTU domain, deubiquitinating YAP at multiple lysine sites (K90, K280, K343, K494, and K497), thereby inhibiting its degradation.^339^ USP19 stabilizes YAP in HCC by removing K48- and K11-linked Ub chains at K76 and K90.^340^ CYLD suppresses YAP ubiquitination, promoting the transcription of ACSL4 and TFRC mRNAs.^341^ USP36 enhances YAP stability by blocking K48-linked polyUb.^342^ Analogously, USP20 directly interacts with YAP1, inhibiting its K48-linked polyUb and promoting stability.^343^ JOSD1, a critical DUB, facilitates colon cancer progression by impeding the K48-linked polyUb of YAP.^344^ USP12 stabilizes YAP by inhibiting K48-linked polyUb, particularly at the K315 site.^345^ ATXN3, a deubiquitylase, stabilizes YAP in pancreatic cancer through its deubiquitination activity.^346^ Additionally, Machado–Joseph disease (MJD), also known as spinocerebellar ataxia type 3 (SCA3), is a progressive NDD caused by a mutation in the *ATXN3* gene.^347^ This condition is described in detail in the “Developmental abnormalness” section. Furthermore, USP49, a novel DUB of YAP1, promotes gastric cancer cell proliferation, metastasis, and chemoresistance.^348^ USP10 inhibits YAP1 ubiquitination, promoting Cyr61 expression and immune escape in pancreatic adenocarcinoma.^349^ EIF3H dissociates PUCs from YAP through a catalytic triad (Asp90, Asp91, and Gln121), stabilizing YAP and promoting tumor invasion and metastasis.^350^ USP9X deubiquitinates and stabilizes YAP1, supporting cancer cell survival.^351^

MAP3K3 phosphorylates YAP at Ser 405, a modification that impedes the binding of FBXW7 to YAP. This interference prevents p62-mediated lysosomal degradation of YAP, thereby stabilizing it.^352^ Additionally, CBX4 plays a crucial role in maintaining stability and facilitating the cytoplasm-to-nuclear transport of YAP1, a central component of the Hippo pathway. CBX4 achieves this by inducing SUMO1 modification at lysine residues K97 and K280, which competitively inhibits phosphorylation at YAP1-S127.^353^ Furthermore, NEDD8 significantly contributes to the regulation of the Hippo–YAP signaling pathway by mediating the neddylation of the transcriptional coactivator YAP1, highlighting its importance in pathway activation and function.^354^

The regulation of TAZ through ubiquitination and, in particular, deubiquitination is a critical mechanism influencing its stability and activity. DUB1 interacts with the TAZ protein and deubiquitinates it at multiple lysine residues, thereby stabilizing TAZ and enhancing its functional activity.^355^ USP26, a member of the USP family, has been identified as a genuine deubiquitinase of TAZ in ATC.^356^ USP7 interacts with TAZ, deubiquitinates it, and stabilizes it by selectively removing K48-linked ubiquitination chains, independent of the canonical Hippo kinase cascade. This action of USP7 effectively counteracts βTRCP-mediated ubiquitin-proteasomal degradation of TAZ, enhancing its nuclear retention and transcriptional activity.^357^ Similarly, USP26 interacts with, deubiquitylates, and stabilizes TAZ in a manner dependent on its deubiquitination activity.^356^ USP43 also interacts with and stabilizes TAZ by inhibiting its ubiquitination. DUB1 associates with TAZ and deubiquitinates it at several lysine residues, thereby stabilizing TAZ and facilitating its function.^358^ USP1 interacts with the WW domain of TAZ, increasing its stability by suppressing K11-linked polyUb.^359^ Collectively, these DUBs, including USP26, play crucial roles in stabilizing TAZ through deubiquitination, thereby modulating its functional outcomes.

The regulation of YAP/TAZ downstream effector TEADs also involves ubiquitination and SUMOylation. RNF214 catalyzes nonproteolytic ubiquitination at a conserved lysine residue of TEADs, enhancing the interaction between YAP and TEADs and promoting the transactivation of downstream genes in the Hippo signaling pathway.^360^ Additionally, TEAD1 undergoes SUMO1 modification during cardiac hypertrophy, a process regulated by the SUMO-specific protease SENP1. Lysine 173 is a critical site for TEAD1 SUMOylation, influencing its protein stability, nuclear localization, and DNA-binding capacity. This modification also strengthens the interaction between TEAD1 and its transcriptional coactivator YAP1, further amplifying Hippo pathway signaling.^361^

The regulation of LATS1/2, upstream components of the YAP/TAZ pathway, involves ubiquitination, deubiquitination, and even SUMOylation. For example, ITCH catalyzes the ubiquitination of LATS1, stimulating its proteasomal degradation. This ITCH-mediated degradation of LATS1 is associated with enhanced cell growth, induction of epithelial‒mesenchymal transition (EMT), and increased tumorigenicity.^362^ GPRC5A interacts with WWP1, facilitating polyUb and degradation of LATS1, thereby activating the YAP1 signaling pathways critical for metastasis.^363^ SPOP specifically interacts with LATS1, promoting its polyUb and subsequent degradation in a degron-dependent manner.^364^ NEDD4 directly interacts with LATS1, leading to its ubiquitination and decreased levels, which enhances YAP nuclear localization and transcriptional activity. NEDD4 thus acts as an additional regulator of the Hippo pathway via interactions between WW domain-containing and PPxY motif-containing proteins.^365^ WWP2 interacts with LATS1, resulting in its ubiquitination and degradation, leading to increased YAP1 transcriptional activity.^137^ FBXW5 promotes the ubiquitination and degradation of LATS1.^366^ S100A16 destabilizes LATS1 by inducing CUL4A-mediated ubiquitination and degradation.^367^ PRAMEF2, a BC-box-containing substrate recognition subunit of the Cullin 2-based E3 ubiquitin ligase complex, mediates the polyUb of LATS1, leading to its proteasomal degradation.^368^ RNF106 associates with LATS2, facilitating its K48-linked ubiquitination and degradation, which inhibits YAP phosphorylation and promotes the oncogenic function of YAP in esophageal squamous cell carcinoma (ESCC).^369^ BAP1 limits tumor progression by stabilizing LATS, thereby promoting the activity of the Hippo tumor suppressor pathway, with LATS2 being a substrate of BAP1.^370^ FBXO22 directly interacts with and destabilizes LATS2, a critical regulator of the Hippo pathway.^371^ CMTM5 regulates Hippo/YAP signaling to inhibit cell growth and invasion and promote ferroptosis in glioma by modulating WWP2-mediated LATS2 ubiquitination, thereby attenuating glioma progression.^372^ Derepressed CRL4^DCAF1^ promotes YAP- and TEAD-dependent transcription by ubiquitinating and inhibiting LATS1 and LATS2 in the nucleus.^373^ The APC/C^Cdh1^ E3 Ub ligase complex, which is crucial for eukaryotic cell cycle progression, intrinsically regulates Hippo signaling by mediating LATS degradation, representing an evolutionarily conserved layer of Hippo signaling regulation.^374^ Interestingly, SUMO1 interacts with and directly SUMOylates LATS1. Disruption of the SUMOylation pathway impairs LATS1 SUMOylation,^375^ highlighting the functional significance of this PTM in regulating LATS1 activity and its role in the Hippo signaling pathway. The RING ligase PRAJA2 ubiquitinates and targets MOB, a core component of the NDR/LATS kinase complex and a positive regulator of the tumor-suppressive Hippo signaling cascade, for degradation. This ubiquitin‒proteasome-mediated degradation of MOB attenuates Hippo pathway activity, thereby promoting GB growth.^376^

The upstream regulator of LATS1/2-MOB1, MST1/2, is also subject to ubiquitination and nonubiquitination modifications. Chemical inhibition of proteasomal proteolysis increases MST2 levels in human breast epithelial cells. MST2 interacts with the βTRCP E3 Ub ligase, and silencing βTRCP leads to MST2 accumulation. Site-directed mutagenesis and computational molecular dynamics studies revealed that βTRCP binds MST2 through a noncanonical degradation motif.^377^ TRIM21 directly interacts with and ubiquitinates MST2 at K473 via K63-linked ubiquitination. This modification promotes MST2 homodimer formation, enhances MST2 kinase activity, and functionally inactivates YAP, thereby inhibiting EMT.^378^ RNF128 regulates MST protein stability and the expression of Hippo pathway target genes.^379^ Additionally, Smurf1-mediated polyUb at K285/K282 of MST1/2 destabilizes these kinases, attenuating their tumor-suppressor functions.^380^ Ubiquitination and deubiquitination critically regulate the Hippo pathway, which governs organ size, tissue homeostasis, and tumor suppression. These modifications fine-tune the stability, localization, and activity of core components such as MST1/2, LATS1/2, YAP/TAZ, and TEADs. E3 ligases (e.g., βTRCP, ITCH, and NEDD4) target Hippo proteins for degradation, while DUBs (e.g., USP9X, OTUB1, and CYLD) stabilize them, promoting their nuclear translocation and transcriptional activity. This dynamic balance ensures precise Hippo signaling in response to cellular cues.

### The Notch signaling pathway and its regulation by ubiquitination and deubiquitination

The Notch signaling pathway consists mainly of Notch receptors (Notch1–4) and ligands (Delta-like ligands 1/3/4, Jagged 2, Jagged 1).^381^ Ligand binding to the receptor initiates a three-step cleavage process mediated by gamma-secretase, which releases the Notch intracellular domain (NICD) into the nucleus.^381^ This event subsequently activates the transcription of Notch target genes.^381^ Over the past decade, genetic screens in *Drosophila* and other model organisms, coupled with detailed biochemical analyses, have begun to highlight the critical role of ubiquitination in regulating Notch activity. Several E3 Ub ligases have been identified as regulators of Notch receptor ubiquitination.^382^ The suppressor of deltex (Su(dx)) was first characterized in *Drosophila* as a negative regulator of Notch signaling, acting antagonistically to the E3 ligase Deltex, which was identified in later studies.^383^ Along with two other E3 ligases, c-Cbl and NEDD4, Su(dx) is involved in the sorting and lysosomal degradation of the unactivated Notch receptor.^384,385^ FBXW7 has been shown to ubiquitinate the NICD, leading to its proteasomal degradation.^386^ Additionally, research from multiple laboratories has demonstrated that two distinct E3 ligases, Neuralized and Mind bomb, promote the monoubiquitination of the ligand proteins Delta and Serrate, facilitating their endocytosis.^387^ Recent in vivo screening confirmed that at least four DUBs—USP10, eIF3H, eIF3F, and BAP1—regulate Notch signaling in breast cells via the ubiquitin‒proteasome pathway (UPP).^388^ In the context of TNBC, under the cellular stress conditions typical of the tumor microenvironment (TME), USP9x forms a multiprotein complex with the pseudokinase tribbles homolog 3 (TRB3), which collectively activates the Notch pathway.^389^ Knockdown of USP9x impairs Notch activation, leading to reduced production of IL-1β and C-C motif chemokine ligand 2 (CCL2).^389^ Pharmacological inhibition of USP9x via G9, a partially selective small-molecule inhibitor, diminishes Notch activity, reshapes the tumor immune landscape, and inhibits tumor growth without observable toxicity.^389^ Silencing USP12 specifically impairs Notch trafficking to lysosomes, leading to the accumulation of receptors on the cell surface and increased Notch activity.^390^ Biochemically, USP12, together with its activator USP1-associated factor 1, deubiquitinates the inactive form of Notch in both cell culture and in vitro.^390^

### The hedgehog signaling pathway and its regulation by ubiquitination and deubiquitination

The hedgehog (Hh) signaling pathway was initially identified in *Drosophila* as a pivotal regulator of segmental patterning during embryonic development.^391^ Activation begins when one of the three secreted and lipid-modified Hh ligands—Sonic (SHh), Desert (DHh), or Indian (IHh)—binds to Patched receptor 1 (Ptch1), a 12-pass transmembrane protein.^392^ In the absence of a ligand, Ptch1 inhibits the activity of Smoothened (Smo), a 7-pass transmembrane protein homologous to G protein-coupled receptors.^392^ Upon ligand binding to Ptch1, this repression is relieved, leading to the modulation of the expression and/or posttranslational processing of the three Gli zinc-finger TFs.^392^ Glioma-associated oncogene homolog 1 (Gli1) acts as a transcriptional activator, whereas Gli3 serves as a repressor. Gli2 can function either as an activator or repressor, depending on its posttranscriptional activity and PTMs.^393^ The balance between the active and repressive forms of Gli proteins regulates the expression of target genes, including Ptch1 and Gli1.^394,395^ The Hh signaling pathway has been shown to be regulated through the UPS. For example, USP37 was found to be overexpressed in breast cancer stem cells (BCSCs) and to be involved in regulating cell invasion, EMT, stemness, and cisplatin sensitivity in breast cancer cell lines.^396^ Furthermore, USP37 knockdown reduces tumorigenicity and enhances the anticancer effects of cisplatin in vivo.^396^ Silencing USP37 significantly decreased the levels of Hh pathway components, including Smo and Gli-1.^396^ Mechanistically, USP37 stabilizes Gli-1, and the two proteins interact with each other.^396^ The mechanism by which USP48 regulates SHh closely resembles that of USP37, specifically through its interaction with Gli1 and direct deubiquitination of the protein.^397^ In GB cells, knockdown of USP48 inhibits cell proliferation and the expression of Gli1 downstream targets, thereby suppressing GB tumorigenesis.^397^ Additionally, the SHh pathway induces USP48 expression through Gli1-mediated transcriptional activation, thereby establishing a positive feedback loop that regulates Hh signaling.^397^ Recent research has demonstrated that Smurf1 and Smurf2 bind to GLI1, utilizing the proline-rich regions of the GLI1 protein.^398^ This interaction induces polyUb and proteasomal degradation of GLI1, thereby suppressing Hh pathway activity and inhibiting Hh-dependent tumor cell proliferation.^398^ Interestingly, linear ubiquitination helps stabilize Gli proteins, which results in the noncanonical activation of Hh signaling in colorectal cancer (CRC) cells.^399^ Moreover, LUBAC promotes tumor growth in these cells. An increase in the expression of LUBAC components was also observed in CRC tissues, with higher expression levels being associated with a poorer prognosis in CRC patients.^399^ In a recent study, Chen et al. demonstrated that TGF-β1 elevated the protein levels of USP22.^400^ This increase in USP22 activity facilitates the deubiquitination and subsequent activation of BRD4, which in turn induces the expression of GLI1 and osteopontin (OPN), thereby promoting the proliferation of airway smooth muscle cells (ASMCs).^400^

### The TGF-β signaling pathway and its regulation by ubiquitination and deubiquitination

TGF-β is a member of the TGF-β superfamily, which is a group of structurally related cytokines.^401^ TGF-β family members interact with two distinct types of transmembrane Ser/threonine kinase receptors: type II and type I.^402^ The intracellular signaling cascade initiated by these ligands is primarily mediated by Smad proteins.^403^ Upon activation, type I receptor kinases phosphorylate receptor-regulated Smads (R-Smads), which include Smad3 and Smad2 (which are specific to TGF-β/activin signaling) and Smad8, Smad5, and Smad1 (which are specific to bone morphogenetic protein (BMP) signaling), typically at a conserved C-terminal SXS motif.^404^ Phosphorylated R-Smads associate with Smad4, the common mediator Smad (Co-Smad), and translocate into the nucleus to cooperate with transcription factors to regulate target gene expression.^405^ Inhibitory Smads (I-Smads), namely, Smad7 and Smad6, are induced by TGF-β family ligands and function as negative regulators of TGF-β/BMP signaling, thus establishing a negative feedback loop.^406^ While Smad7 broadly inhibits both BMP and TGF-β signaling,^407^ Smad6 primarily suppresses BMP signaling and exerts only modest inhibitory effects on TGF-β/activin signaling.^408,409^

**Fig. 8:**
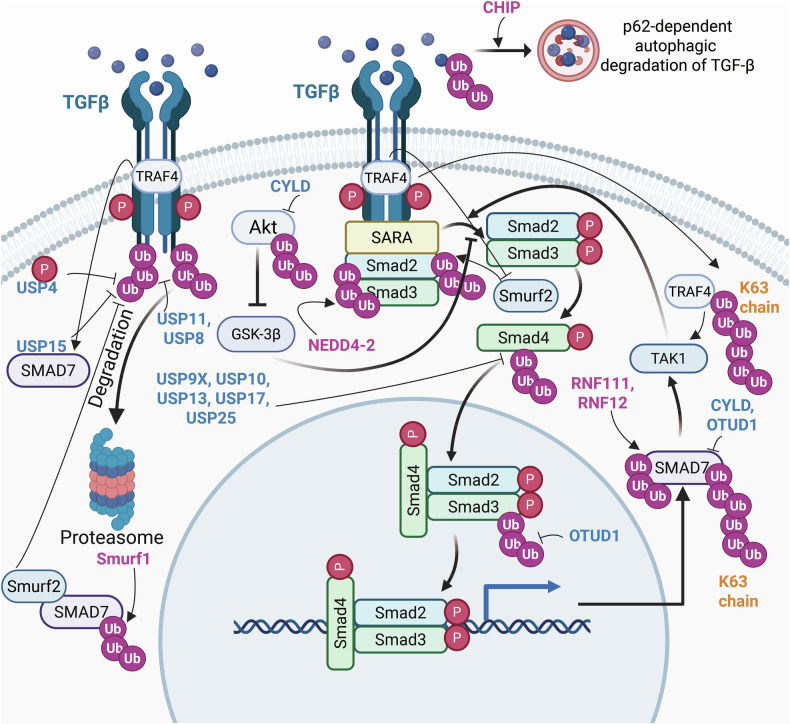
Ubiquitination and deubiquitination in the transforming growth factor-beta (TGF-β) pathway. This figure illustrates the critical roles of ubiquitination and deubiquitination in the regulation of the TGF-β signaling pathway, which is essential for controlling processes such as cell growth, differentiation, and immune regulation. These posttranslational modifications influence the stability, function, and subcellular distribution of components such as TGF-β receptors (TβRI/TβRII), Smad proteins, and inhibitory Smads, such as Smad7. Ubiquitin ligases, including Smurf1, Smurf2, and NEDD4-2, promote the degradation of key signaling molecules, thereby dampening the TGF-β pathway. Conversely, deubiquitinating enzymes (DUBs), such as USP4, USP11, USP15, and CYLD, counteract this by removing ubiquitin chains, thus stabilizing pathway components and increasing signal transduction. This intricate interplay between ubiquitination and deubiquitination ensures tight regulation of TGF-β signaling in response to various cellular conditions. The elements were obtained from BioRender.com

In contrast to other signaling pathways, the regulation of TGF-β signaling appears to be more heavily influenced by DUBs than by Ub ligases (Fig. 8). For example, AKT-mediated phosphorylation enables USP4 to directly bind to the activated TGF-β type I receptor, reversing its ubiquitination and promoting TGF-β signaling.^410^ Similarly, USP15 counteracts the polyUb mediated by the E3 Ub ligase Smurf2 and the inhibitory Smad7, stabilizing the TGF-β type I receptor and enhancing TGF-β signaling.^411,412^ USP11 deubiquitinates and stabilizes the TGF-β type II receptor, further promoting TGF-β signaling.^413^ USP8 also directly deubiquitinates and stabilizes TβRII, increasing its expression on the plasma membrane and in tumor-derived extracellular vesicles.^414^ CYLD suppresses TGF-β signaling by reducing SMAD3 protein stability through an AKT-GSK3β-Hsc70-interacting protein-dependent mechanism.^261^ Additionally, CYLD deubiquitinates Lys63-linked PUCs on SMAD7, modulating the activation of the SMAD7-TAK1-TAB2/3 complex and TF activator protein 1 (AP-1). CYLD forms a complex with SMAD7, facilitating the deubiquitination of SMAD7 at lysine residues 360 and 374, which further fine-tunes its regulatory role in TGF-β signaling.^415^ Members of the USP family, including USP9X,^416^ USP10^417^, USP13,^418^ USP17,^419^ and USP25,^420^ have been reported to regulate Smad4 deubiquitination. On the ubiquitination side, CHIP ubiquitinates TGF-β at the K315 site, triggering p62-dependent autophagic sorting and degradation.^421^ NEDD4-2, another E3 ligase, promotes the proteasomal degradation of phosphorylated Smad2/3 induced by TGF-β.^422^ Smurf1 and Smurf2, both E3 Ub ligases, target Smad7 for degradation,^423^ with Smurf2 also capable of initiating the polyUb and degradation of Smad2.^424,425^ Arkadia (also known as RNF111) modulates TGF-β signaling by targeting Smad7 for degradation,^426^ whereas RNF12 plays a regulatory role in embryonic stem cell development and zebrafish morphogenesis through Smad7 degradation.^427^

Importantly, TRAF4 acts as a key effector in pro-oncogenic TGF-β signaling, modulating both SMAD-dependent and SMAD-independent pathways.^428^ Upon TGF-β activation, TRAF4 is recruited to the receptor complex, where it inhibits Smurf2 and facilitates USP15 recruitment to the type I receptor (TβRI).^428^ This interaction stabilizes TβRI on the plasma membrane and enhances TGF-β signaling.^428^ Furthermore, the TGF-β-induced interaction between TβR and TRAF4 results in the Lys63-linked polyUb of TRAF4, activating TAK1.^428^ Ubiquitination and deubiquitination are central to the regulation of TGF-β signaling, which governs cell proliferation, differentiation, and immune responses. These modifications control the stability, activity, and localization of TβR (TβRI/TβRII), SMADs, and inhibitory SMADs (e.g., SMAD7). E3 ligases such as NEDD4-2, Smurf2, and Smurf1 degrade TGF-β components, attenuating signaling, whereas DUBs such as USP4, USP11, USP15, and CYLD stabilize them, enhancing signaling. This dynamic balance ensures precise TGF-β regulation in response to cellular cues.

### The DNA damage repair signaling pathway and its regulation by ubiquitination and deubiquitination

**Fig. 9:**
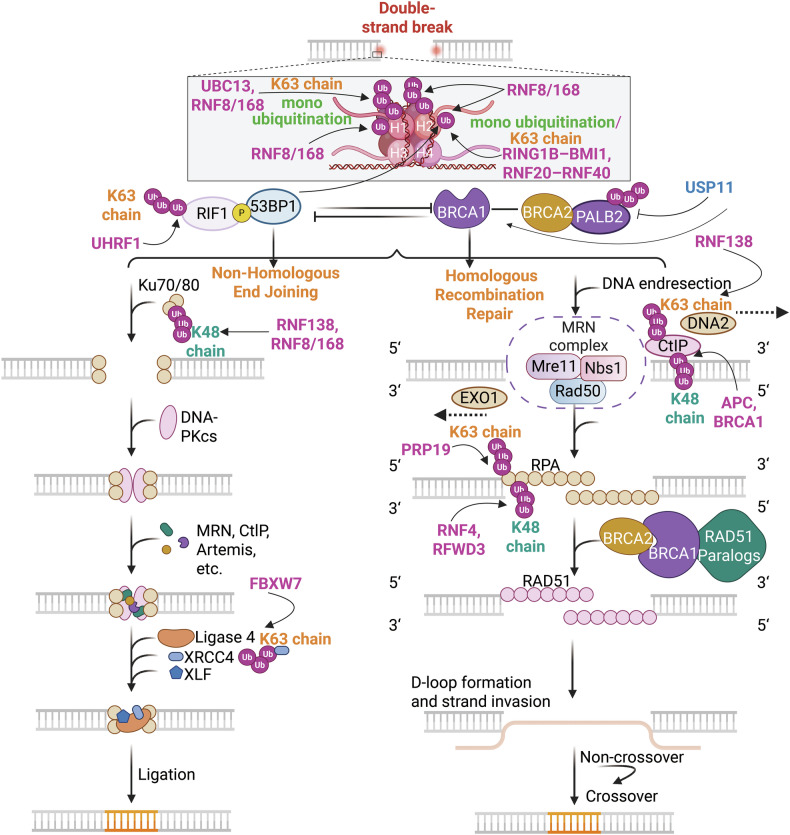
Ubiquitination and deubiquitination in the DNA damage repair (DDR) signaling pathway. This figure highlights the pivotal role of ubiquitination and deubiquitination in regulating the DDR pathway, which is essential for maintaining genomic stability. E3 ligases such as RNF8, RNF168, and BRCA1–BARD1 target proteins and histones at damage sites, facilitating the recruitment of repair factors, chromatin remodeling, and the decision between NHEJ and HR. Deubiquitinating enzymes (DUBs), including USP11 and OTUB1, counterbalance these modifications, finely tuning the repair process and preserving genomic stability. Ubiquitination also regulates the stability, localization, and activity of DDR proteins, such as RPA, CtIP, and 53BP1, in coordination with other posttranslational modifications. This intricate ubiquitin signaling network is crucial for precise DNA repair, safeguarding genomic integrity, and preventing diseases such as cancer. The elements were obtained from BioRender.com

The preservation and integrity of DNA are fundamental to the survival and fitness of all living organisms.^429^ Genomic stability is constantly challenged by a myriad of nucleolytic assaults, both endogenous and exogenous, that threaten DNA integrity.^430^ Among the diverse array of DNA lesions, DSBs are exceptionally cytotoxic.^430^ Unrepaired DSBs can lead to significant genetic information loss, often culminating in cell death.^431^ To counteract these threats, cells have evolved a sophisticated DDR, an intricate network of interconnected pathways activated in response to widespread genotoxic stress.^431^ Eukaryotic cells primarily employ two major mechanisms to repair DSBs, nonhomologous end joining (NHEJ) and homologous recombination (HR), along with their respective subpathways, ensuring the maintenance of genomic fidelity^432,433^ (Fig. 9).

Signaling mediated by Ub, SUMO, and other UBLs intricately coordinates and regulates cellular responses to DNA DSBs across multiple levels, frequently involving extensive crosstalk between these modifications.^37^ However, owing to space constraints, this discussion focuses primarily on the regulatory role of ubiquitination in the DDR. For example, recent studies have revealed an additional regulatory layer within the RNF8/RNF168 cascade. Specifically, the E3 ligase RNF8, in conjunction with the E2-conjugating enzyme UBC13, mediates the K63-linked ubiquitination of H1 linker histones. This modification creates a binding platform for RNF168, further amplifying the ubiquitin-dependent signaling required for DSB repair.^434^ The RING1B–BMI1 complex monoubiquitylates histone H2A at lysine residues 119 and 120 (H2AK119ub and H2AK120ub), a modification that facilitates the transcriptional silencing of damaged chromatin.^435–437^ In contrast, the RNF20–RNF40 complex monoubiquitylates histone H2B at lysine 120 (H2BK120ub), promoting chromatin remodeling and HR.^438^ Additionally, UHRF1 is recruited to DNA DSBs by BRCA1 during S phase, a process dependent on the BRCT domain of BRCA1 and the phosphorylation of UHRF1 at Ser674. Following recruitment, UHRF1 catalyzes the K63-linked polyUb of RIF1, leading to its dissociation from 53BP1 and DSB sites. This mechanism facilitates the initiation of HR, positioning UHRF1 as a critical regulator of DSB repair pathway choice.^439^ Furthermore, USP11 removes Ub from PALB2, facilitating the assembly of the BRCA1–PALB2–BRCA2 complex in S/G2-phase cells.^440^ This activity enhances HR repair by stabilizing critical interactions among key DNA repair proteins, ensuring efficient DNA damage resolution. RNF138 plays a crucial role in enhancing HR by ubiquitinating two key substrates. It first ubiquitinates CtIP, facilitating its retention at DNA DSBs.^441^ Additionally, RNF138 targets the Ku80 subunit of the Ku70/80 heterodimer for ubiquitination, promoting its displacement from DSBs. Ubiquitination of Ku80 occurs exclusively during the S phase, indicating the presence of a cell cycle-dependent regulatory mechanism that fine-tunes this process.^442^ BRCA1–BARD1, a key HR factor, ubiquitinates several proteins at DNA DSB sites, including CtIP and H2A. However, the precise role of its E3 ligase activity in DNA repair remains unclear.^443,444^ In addition to the S and G2 phases, CtIP expression is maintained at low levels through ubiquitination by the APC/C.^445^ RNF4, a SUMO-targeted Ub ligase (STUbL), ubiquitinates sumoylated MDC1 and RPA, supporting the RNF8–RNF168 pathway and HR.^446,447^ RFWD3 ubiquitinates RPA to promote HR at stalled replication forks, whereas PRP19 ubiquitinates RPA to facilitate ATR activation.^448^ Additionally, upon phosphorylation at Ser325/326 by DNA-PKcs, XRCC4 is recognized and ubiquitinated by FBXW7 via K63-linked chains at lysine 296, further integrating Ub signaling into DNA repair processes.^449^ Ubiquitination plays a pivotal role in the DDR, serving as a versatile and dynamic regulatory mechanism that orchestrates the repair of DNA lesions, particularly DSBs. Through the action of E3 Ub ligases such as RNF8, RNF168, and BRCA1–BARD1, ubiquitination marks key proteins and histones at damage sites, facilitating the recruitment of repair factors, chromatin remodeling, and pathway selection between NHEJ and HR. DUBs, including USP11 and OTUB1, counterbalance these modifications to fine-tune repair processes and maintain genomic stability. Ubiquitination also regulates the stability, localization, and activity of critical DDR players, such as RPA, CtIP, and 53BP1, often in coordination with other PTMs, such as phosphorylation and SUMOylation. This complex network of Ub signaling ensures precise control over DNA repair, highlighting its essential role in preserving genomic integrity and preventing diseases such as cancer. Future research into the spatiotemporal regulation of ubiquitination and its crosstalk with other modifications will deepen our understanding of DDR mechanisms and open new avenues for therapeutic intervention.

## Ubiquitination and deubiquitination in diseases

Ubiquitination and deubiquitination are pivotal in regulating numerous biological processes across the human body. Proteins, which are fundamental to human physiology, carry out specific functions that are tightly coordinated to control various biological pathways. Dysregulation of the ubiquitination or deubiquitination of key proteins can disrupt these processes, potentially leading to disease. Increasing evidence has linked abnormal regulation of these pathways to the onset of several conditions, including cancers, NDDs, CVDs, inflammatory diseases, autoinflammatory disorders and developmental abnormalities. The following sections explore the impact of ubiquitination and deubiquitination dysregulation in the context of these diseases.

### Ubiquitination and deubiquitination in cancer

Ubiquitination and deubiquitination are pivotal regulators of cancer development and progression, influencing various aspects of tumor biology across four key dimensions. First, they modulate core oncogenic traits that orchestrate multiple oncogenic programs through distinct mechanisms: (i) sustaining proliferative signaling via activation of the EGFR and PI3K/AKT pathways; (ii) enabling evasion of growth suppressors through degradation of tumor suppressors (e.g., p53); (iii) conferring resistance to apoptosis by modulating Bcl-2 family protein dynamics; (iv) maintaining replicative immortality through telomerase stabilization; (v) inducing angiogenesis; and (vi) promoting metastatic competence through ECM remodeling and the regulation of motility (Table S1).^450^ Second, in terms of genomic and epigenetic regulation, ubiquitination and deubiquitination maintain genome stability through DNA damage repair mechanisms but can also contribute to genome instability and mutation when dysregulated. They further drive nonmutational epigenetic reprogramming by modifying histones and chromatin remodelers, thereby enhancing the phenotypic plasticity that aids in cancer cell adaptation and survival (Table S2).^450^ Third, these processes shape the TME and immune regulation by promoting tumor-associated inflammation via NF-κB and cytokine signaling, enabling immune evasion through the degradation of immune checkpoint proteins and antigen-presenting molecules and influencing host‒microbe interactions that impact tumor progression (Table S3).^450^ Finally, ubiquitination and deubiquitination regulate metabolic and cellular states by reprogramming energy metabolism and adjusting the stability of metabolic enzymes and transporters to meet the heightened demands of rapidly proliferating cancer cells. They also influence cellular senescence by controlling the turnover of senescence-associated proteins, thereby affecting tumor dormancy or progression (Table S4).^450^ Through these multifaceted mechanisms, ubiquitination and deubiquitination intricately orchestrate the complex biological behaviors of cancer cells, underscoring their potential as therapeutic targets for cancer intervention.

### Ubiquitination and deubiquitination in NDDs

The UPS serves as the primary proteolytic machinery in human cells, critically regulating fundamental biological processes, including cellular differentiation, migratory behavior, and fate determination mechanisms essential for neural development and homeostasis. NDDs such as Alzheimer’s disease (AD), Parkinson’s disease (PD), Huntington’s disease (HD), and amyotrophic lateral sclerosis (ALS) share a common pathological hallmark featuring the deposition of misfolded protein aggregates and the presence of cytoplasmic inclusions.^451^ These proteotoxic manifestations are intrinsically linked to UPS dysfunction, which compromises the efficient clearance of damaged or aberrantly folded proteins within neural tissue. For example, in AD, the microtubule-associated protein tau undergoes dysregulated ubiquitination coupled with hyperphosphorylation, culminating in its pathological aggregation. The spatiotemporal dysregulation of tau-associated Ub ligases and DUBs reveals compartment-specific proteostatic collapse in AD. E3 ligases exhibit Braak stage-dependent regional disparities: CHIP shows elevated temporal cortical protein with hippocampal depletion (Braak III–VI) despite stable mRNA,^452^ whereas TRAF6 coaggregates with tau-p62 complexes in hippocampal inclusions (Braak VI).^453^ Axotrophin/MARCH7 is associated with neurofibrillary tangles (NFTs) but decreases in cytoplasmic/nuclear fractions (Braak II–VI),^454^ in contrast with HRD1’s inverse correlation with phosphorylated tau (p-tau) burden in hippocampal neurons despite its upregulation of mRNA expression.^455^ TRIAD3A depletion in Braak V–VI cortices parallels tau–amyloid coaggregation.^456^ DUBs display sex- and compartment-biased dysregulation: USP9 decreases in male late-onset AD patients,^457^ whereas USP11 shows female-predominant cortical elevation linked to p-tau pathology.^458^ USP14 transcriptional downregulation is correlated with aging,^459^ whereas UCH-L1 is associated with oxidative modification and paradoxical solubility shifts—hippocampal accumulation contrasts with frontal cortical depletion, where soluble UCH-L1 inversely predicts NFT burden.^460^ Protein aggregation and accumulation in inclusions, often associated with Ub, are defining characteristics of many NDDs. In AD, tauopathies, and polyglutaminopathies, the mutant Ub form (UBB + 1), derived from an aberrant transcript, colocalizes with key pathological features, including NFTs and neuritic plaques.^461^ Research has demonstrated that the C-terminal extension of UBB + 1 is too short to undergo effective degradation.^462^ Dennissen et al. reported that UCH-L3 is capable of cleaving the C-terminus of UBB + 1.^463^ Moreover, UCH-L3 oxidation impairs its hydrolytic activity, suggesting that dysfunctional UCH-L3 may contribute to full-length UBB + 1 accumulation in various diseases.^463^ A recent study using a 3D neural culture system derived from human neural progenitors demonstrated that UBB + 1 competes with Ub for binding to the deubiquitinating enzyme UCH-L1, resulting in increased levels of amyloid precursor protein (APP), secreted Aβ peptides, and amyloid-β accumulation.^464^ Importantly, suppressing UBB + 1 expression prevents the emergence of AD-associated features. A recent study has shown that this bidirectional UPS dysfunction (ligase suppression/DUB hyperactivity) establishes a phase transition-driven proteolytic bottleneck, where solubility loss, liquid‒liquid phase separation, and sex-specific regulatory failures collectively enable tau aggregation beyond proteostatic capacity.

PD has dual etiological origins, with ~85% of cases classified as sporadic and 15% linked to inherited genetic defects, both of which converge on UPP dysfunction.^465^ Studies have identified PSMC4 (OR 0.73; 95% CI 0.60–0.89) as a strong predictor of PD, with its levels linked to both the onset and progression of the disease.^466,467^ Familial PD has been particularly instrumental in delineating UPP’s pathogenic role through well-characterized mutations: *PARK2* (encoding the RBR-type Ub ligase parkin, featuring an N-terminal ubiquitin-like domain) and *PARK7* (encoding DJ-1, whose neuroprotective activity requires SUMO1 conjugation) represent critical nodes in ubiquitin-like modifier biology.^465,468^ Dominant *LRRK2* mutations, the most prevalent cause of late-onset inherited PD, drive pathogenic cascades via its ubiquitination by the CHIP E3 ligase.^469^ UCH-L1, a deubiquitinating enzyme implicated in both familial and sporadic PD, undergoes *S*-nitrosylation within Lewy bodies, destabilizing its structure and promoting α-synuclein coaggregation.^470^ This multilayered UPP dysregulation spans Ub ligase activity (parkin, CHIP), deubiquitination (UCH-L1), and ubiquitin-like signaling (SUMO-DJ-1), collectively disrupting the precision of the Ub code in maintaining proteostasis. The mechanistic overlap between genetic and sporadic forms underscores UPP as a unifying axis in PD pathogenesis, where inherited mutations illuminate pathways subsequently validated in idiopathic disease through protein misfolding and aggregation dynamics.

HD, driven by mutations in the *huntingtin* (*HTT*) gene, is characterized by UPP dysfunction mechanistically linked to aberrant Lys48-linked polyUb.^471^ Pioneering mass spectrometry studies revealed pathological accumulations of Lys48-specific Ub chains—the canonical proteasomal degradation signal—in human HD postmortem brains, R6/2 transgenic mice, and Q150/Q150 knock-in models, implicating impaired substrate processing as a conserved hallmark.^472^ Functional dissection identified UBE3A, a HECT-domain E3 ligase, as a direct mediator of mutant HTT (mHTT) Lys48 ubiquitination in HEK293 models,^473^ whereas genome-wide association studies identified UBR5, another HECT ligase, as a modifier of HD onset age.^474^ This dual ligase involvement underscores a Ub code rewiring phenomenon in HD, where dysregulated HECT family enzymes disrupt the stoichiometry of degradation signals, creating a proteostatic “traffic jam” that exacerbates mHTT aggregation. Critically, the evolutionary conservation of Lys48 chain accumulation across species and model systems positions UPP failure not only as a secondary consequence but also as a central driver of HD pathogenesis, bridging the genetic etiology of the proteotoxic crisis.

ALS exemplifies a dual-edged Ub code dysregulation in motor neuron proteostasis, where UPP dysfunction exacerbates both mutant SOD1 and TDP-43 toxicity.^475,476^ Mutant *SOD1*, which is implicated in familial and sporadic ALS, undergoes accelerated UPP-mediated degradation orchestrated by specialized E3 ligases: dorfin and NEDL1 selectively ubiquitinate mutant SOD1 to mitigate its cytotoxicity,^477,478^ whereas ER-associated Gp78 tags misfolded SOD1 for proteasomal clearance, suppressing aggregation.^479^ Paradoxically, Smurf1 catalyzes the K63-linked ubiquitination of SOD1, redirecting toxic species to aggresome-autophagy pathways in neuronal models.^480^ This proteostatic balancing act collapses in SOD1-G93A mice, where proteasome subunit downregulation cripples UPP capacity.^480^ ALS-linked TDP-43 aggregates recruit a distinct Ub ligase network, parkin, Rnf220, Znf179, and Praja1, that attempts to triage misfolded TDP-43 via UBE2E-mediated ubiquitination.^465^ The coexistence of compensatory ubiquitination (SOD1 clearance) and maladaptive Ub signaling (TDP-43 aggregation) reflects broader proteostatic failure: ALS-linked ligases engage in futile cycles of substrate targeting, overwhelmed by the kinetic dominance of misfolding. Critically, the evolutionary conservation of these mechanisms, from HEK293 cells to transgenic mice, positions UPP dysfunction not as a bystander but as a catalyst of motor neuron vulnerability, where Ub code miscalculations transform protective degradation into an aggregation cascade.

### Ubiquitination and deubiquitination in CVDs

The UPS governs multifaceted regulatory networks involved in cardiomyocyte homeostasis, coordinating fibrotic matrix reorganization, lipidomic stabilization, glucoregulatory fidelity, hypertrophic signaling cascades, ischemia‒reperfusion adaptive responses, and interlinked apoptotic‒ferroptotic fate determination. Crucially, the spatiotemporal equilibrium between the ubiquitination and deubiquitination of master regulatory hubs (e.g., TβR complexes) constitutes a linchpin mechanism in cardiac fibrogenesis. UPS-mediated modulation of TGF-β signaling—which spans receptor activation, Smad2/3/4 transcriptional machinery assembly, and Smad7-mediated negative feedback—serves as the cornerstone of its fibrotic regulation. In addition to this canonical axis, the UPS concurrently regulates TGF-β-independent profibrotic effectors, including p53-mediated senescence pathways, AKT1‒p38 MAPK crosstalk, and JNK1/2-driven inflammatory mediators.^481^ These context-dependent regulatory modalities, particularly E3 ligase-DUB counteractions (as previously delineated), create a dynamic “Ub rheostat” that fine-tunes fibrotic pathogenesis, offering stratified therapeutic entry points for distinct fibrotic cardiomyopathy subphenotypes.

The UPS family exerts multifaceted regulatory dominance across distinct cardiomyopathy subphenotypes through disease-specific substrate targeting. In hypertrophic cardiomyopathy pathogenesis, USP2 coordinates with mitochondrial fusion machinery 2 (Mfn2) to counteract angiotensin II-mediated hypertrophy via deubiquitination^482^ while stabilizing junction plakoglobin (JUP) to suppress AKT/β-catenin hypertrophic signaling in the isoproterenol-challenged myocardium.^483^ Parallel mechanisms involve USP10-mediated stabilization of SIRT6 through direct deubiquitination, increased mitochondrial bioenergetic capacity,^484^ and USP12-driven activation of the p300-METTL3 epigenetic axis via substrate stabilization.^485^ Within dilated cardiomyopathy pathophysiology, USP5 orchestrates proteostasis through dual K48/K63 Ub chain editing at the PSMD14-containing proteasome complex,^486^ whereas USP36 maintains genomic integrity via PARP1 deubiquitination—a function abrogated by its catalytically inert *C131A* mutant.^487^ Ischemic cardiomyopathy mechanisms reveal the cardioprotective role of CYLD through m7G methyltransferase 1 (METTL1) stabilization via p53 deubiquitination in myocardial ischemia‒reperfusion injury models.^488^ Diabetic cardiomyopathy involves USP-mediated metabolic reprogramming: USP7 enhances PPARα-driven lipid oxidation through PGC-1β stabilization,^489^ USP10 activates Notch1-mediated cytoprotection in type 2 diabetic infarcted myocardium,^490^ and USP28 maintains mitochondrial homeostasis via PPARα–Mfn2 transcriptional coupling.^491^ These findings underscore the therapeutic potential of targeting USP-specific regulatory nodes within context-dependent ubiquitin‒proteasome axes across cardiomyopathy spectra.

### Ubiquitination and deubiquitination in inflammatory diseases

The UPS operates as a molecular fulcrum in host‒pathogen interplay, balancing immune surveillance and pathogen counterrevolution. While orchestrating antiviral defenses through IFN-stimulated gene networks and proteolytic elimination of invasive agents, the UPS paradoxically serves as a substrate for microbial exploitation—viruses encode autonomous ubiquitin-modifying enzymes (e.g., viral E3 ligases/DUBs) to rewire host Ub codes, stabilizing virion components while evading immune detection. This molecular arms race extends beyond canonical pathogens: STING-mediated nucleic acid-sensing pathways, which are critical for RNA/DNA viral defense, are intricately regulated by ubiquitination‒deubiquitination cycles that modulate the amplitude and duration of inflammation. UPS dysregulation transcends infectious contexts, governing sterile inflammatory pathologies such as osteoarthritis (OA) (via NF-κB–ECM degradation axis modulation) and periodontitis (through TLR4–MyD88 signal fine-tuning). Bacterial pathogens amplify this complexity by secreting E3-mimetic effectors that reprogram host ubiquitinomes, exemplified by Salmonella’s SopA-mediated NF-κB suppression.

UPS dysregulation in OA manifests as a proteostatic crisis in articular cartilage, where E3 ligase network failure drives pathological cascades. Genetic perturbation of *WWP2*—either through full ablation or catalytic inactivation (*C838A* mutation)—exacerbates murine OA progression by destabilizing K48-linked polyUb and proteasomal degradation of Runx2, thereby unleashing its transcriptional activation of the matrix-degrading enzyme Adamts5.^492^ Conversely, the E3 ligase networks FBXO21 and HECTD1 orchestrate distinct autophagic flux pathways to counteract OA pathogenesis: FBXO21 mediates the K63-linked polyUb of ERK1/2 to increase stress-induced autophagy, whereas HECTD1 targets the autophagic checkpoint protein Rubicon for proteasomal degradation via K48-linked polyUb, thereby alleviating the suppression of lysosomal biogenesis.^493,494^ In parallel, ITCH and UFL1 suppress inflammatory cascades in OA pathophysiology—ITCH ubiquitinates and destabilizes NEMO to attenuate canonical NF-κB signaling, whereas UFL1 inhibits Notch-mediated proinflammatory transcription through nondegradative ubiquitination of the NICD.^494^ Paradoxically, FBXO6 loss accelerates posttraumatic OA, whereas its overexpression suppresses MMP13 activation through K48-linked ubiquitination and degradation of MMP14, demonstrating the bidirectional regulatory capacity of UPS components.^495^ USP7-mediated deubiquitination is a nodal regulator of inflammasome activation, amplifying NLRP3-GSDMD/caspase-1 pyroptotic cascades and MMP1/13 overexpression—effects that are abrogated by pharmacological USP7 inhibition in vivo.^496^

Periodontal pathogenesis is governed by a ubiquitin-code regulatory network in which E3 ligases and DUBs bidirectionally control osteoimmune homeostasis. Nrf2 effectively reduced intracellular ROS accumulation and c-FOS phosphorylation during osteoclastic differentiation by modulating antioxidant enzymes, thereby inhibiting RANKL-induced osteoclast differentiation.^497^ FBXL19 and PDLIM2 attenuate inflammatory cascades in osteocytes by degrading CREB and STAT3, respectively, through P.g-LPS-induced proteasomal targeting.^498^ Synoviolin was found to interact with GSDMD. In bone marrow-derived macrophages lacking synoviolin, ATP stimulation led to increased secretion of IL-1β and IL-18. Mice with myeloid cell-specific synoviolin deficiency exhibited more severe periodontitis, accompanied by elevated levels of IL-1β and IL-18.^499^ For osteogenic regeneration, USP12 inhibition rescues PERK/eIF2α/ATF4, IRE1α/XBP1s, and ATF6 axis-mediated mineralization defects in periodontal ligament cells (PDLCs),^500^ whereas USP17 activation enhances BMP4-Osterix osteoblast differentiation.^501^ OTUD1-mediated stabilization of SEC23B in neutrophils establishes a negative feedback loop against periodontal inflammation.^502^ The UPS and DUBs regulate inflammatory signaling, which is essential for immune balance and inflammation. Ubiquitination targets inflammatory regulators such as NF-κB and NLRP3 for degradation or modulation, while DUBs stabilize proinflammatory mediators. In rheumatoid arthritis, A20 inhibits NF-κB by removing K63-linked Ub from RIP1,^503^ and OTULIN deficiency exacerbates inflammation.^504^ Notably, HA20 was found in familial Behçet-like autoinflammatory syndrome,^171^ as described in detail below.

Context-dependent effects, such as the ability of CYLD to suppress osteoclastogenesis in periodontitis but enhance TRAF6/TAK1 signaling in psoriasis, have been reported. Targeted therapies aim at E3 ligases and DUBs, with challenges in cell-type specificity. Future strategies include mapping Ub codes and developing PROTACs to degrade inflammatory effectors. This system is pivotal for immune regulation and inflammation resolution.

### Ubiquitination and deubiquitination in autoinflammatory disorders

Autoinflammatory disorders caused by UPS dysfunction include VEXAS, CANDLE/PRAAS, SWIRM, Myb-like, and MPN domain 1 (MYSM1) deficiency; familial Behçet-like autoinflammatory syndrome; and LUBAC deficiency.

VEXAS syndrome is a rare, late-onset condition characterized by the presence of vacuoles.^98^ It was first described by ref. ^98^ The syndrome is caused by somatic mutations in the X-linked *UBA1* gene,^98^ which encodes Uba1, a key enzyme in the UPS that regulates protein degradation and immune responses.^505^ Affected individuals typically harbor one of three somatic mutations at p.Met41, which results in loss of cytoplasmic Uba1 function through the formation of a catalytically impaired Uba1c isoform.^505^ Targeted sequencing of specific cell types revealed that *UBA1* variants were enriched in hematopoietic progenitors and myeloid cells but absent from T and B lymphocytes and fibroblasts.^505^ In 2024, Kosmider O et al. reported that monocytes in VEXAS syndrome are impaired both quantitatively and qualitatively, exhibiting signs of exhaustion and abnormal chemokine receptor expression.^506^ Circulating blood samples from patients present elevated levels of IL-1β and IL-18, which are indicative of inflammasome activation and myeloid cell dysregulation.^506^ Whole-blood transcriptome profiling further supports these observations by revealing enrichment of TNF-α and NF-κB signaling, which are pathways associated with inflammation and cell death.^506^ Currently, no standardized treatment exists for VEXAS syndrome. Therapeutic efforts are focused primarily on controlling inflammatory and hematologic abnormalities. Frequently employed strategies involve the blockade of cytokines, such as IL-6 inhibitors (e.g., tocilizumab) and JAK-STAT inhibitors (e.g., baricitinib and ruxolitinib), aimed at suppressing systemic inflammation. Future therapies targeting IL-1β and IL-18, along with those directed at the TNF-α and NF-κB signaling pathways, may offer additional effective approaches for managing this disease and merit further investigation. Additionally, further investigations of VEXAS syndrome may offer critical insights into the role of Uba1 and ubiquitylation in human diseases while also revealing potential treatments for affected patients. Such research could pave the way for novel therapeutic approaches, including gene-editing therapies and bone marrow transplantation.

Autosomal recessive mutations in *PSMB8*, which encodes the inducible β5i proteasome subunit, give rise to the immune-dysregulatory disorder known as CANDLE.^507^ This condition is classified as a PRAAS.^507^ Subsequent research indicated that mutations in the following disease-associated genes should also be considered in genetic analyses: PSMB8, PSMA3, PSMB4, PSMB9, PSMB10, POMP, and PSMG2.^18^ Interestingly, unlike many other autoinflammatory disorders, patients with CANDLE/PRAAS do not respond to IL-1 inhibition.^508^ A robust IFN-response gene signature suggests a potential link between IFN dysregulation and proteasome dysfunction, although the specific type of IFN driving this signature remains undefined.^508^ Patients exhibit clinical improvement when treated with Janus kinase inhibitors (JAKis), which attenuate IFN signaling.^509^

MYSM1 was originally identified during investigations of transcriptional regulators and has been shown to mediate histone deubiquitination at lysine 119 of histone H2A, a chromatin modification commonly associated with transcriptional repression.^510^ Deletion of *Mysm1* in mice leads to profound hematopoietic abnormalities, including early developmental arrest in B lymphocytes, impaired stem cell function, defective self-renewal and differentiation, and natural killer cell deficiencies.^511^ In humans, exome sequencing has revealed potentially pathogenic *MYSM1* mutations in two families that presented with bone marrow failure (BMF) and immune system defects.^511^

A20, encoded by *TNFAIP3*, is a DUB essential for suppressing inflammatory and immune responses.^512^ It acts as a negative regulator of the NF-κB signaling cascade.^170^ HA20 was first described in a French family with early-onset Behçet-like syndrome.^171^ In HA20 patients, reduced A20 levels impair deubiquitination, increase IKK complex phosphorylation, and accelerate IκB degradation, activating the NF-κB pathway and increasing proinflammatory cytokine production, contributing to systemic inflammation.^170^ Treatment primarily involves anti-inflammatory strategies, including the IL-1 blocker anakinra, ustekinumab (anti-IL-12/anti-IL-23), and apremilast, an oral PDE-4 inhibitor that modulates multiple inflammatory pathways.^170^ Although these approaches show promising outcomes, their efficacy in pediatric patients with Behçet’s disease remains uncertain, warranting further studies to optimize therapeutic strategies.

Individuals with mutations in LUBAC components display autoinflammation, immunodeficiency, and muscular amylopectinosis, often resulting in early childhood mortality.^513^ HOIP and HOIL-1 deficiencies are inherited as autosomal recessive conditions caused by mutations that either produce truncated proteins or impair the highly conserved PUB domain of HOIP.^514^ Given the critical role of LUBAC in immune signaling, fibroblasts and B cells from affected individuals exhibit impaired NF-κB activation upon stimulation, which contributes to recurrent bacterial infections.^513^ While these fibroblasts exhibit immunodeficiency, peripheral blood mononuclear cells (PBMCs) from HOIP- and HOIL-1-deficient patients remain highly responsive to IL-1 stimulation, producing elevated levels of MIP-1α and IL-6.^513^ Similarly, SHARPIN-deficient patient cells exhibit a reduced canonical NF-κB response and increased susceptibility to TNF-induced cell death.^515^ Both SHARPIN- and HOIP-deficient patients display significant impairments in the development of germinal center B cells in secondary lymphoid tissues.^515^ Remarkably, treatment with anti-TNF therapy in a SHARPIN-deficient patient resulted in complete clinical and transcriptomic resolution of autoinflammation.^515^ Additionally, lymphatic abnormalities observed in both HOIP-deficient patients and mouse models suggest that HOIP may play a LUBAC-independent role in regulating angiogenesis.^513^

### Ubiquitination and deubiquitination in developmental abnormalities

Abberations in the UPS have been implicated in several neurodevelopmental disorders, including AS, Cabezas syndrome, MJD, HAFOUS, and Stankiewicz–Isidor syndrome.

AS is a rare neurodevelopmental condition characterized by profound intellectual disability, motor dysfunction, seizures, and impaired speech. It arises from the loss of the maternally inherited Ub-protein ligase E3A (*UBE3A*) gene, which encodes an E3 Ub ligase.^516^ While UBE3A is normally expressed from both alleles in most tissues, in neurons, its expression is restricted to the maternal allele.^516^ Nuclear UBE3A is highly expressed and is believed to contribute significantly to AS pathogenesis.^517^ UBE3A may play a direct role in regulating APP, in addition to modulating its processing.^518^ Compared with those in healthy controls, increased plasma APP and Aβ peptide levels have been reported in AS patients lacking neuronal UBE3A.^518^ In AS mice, HAP1 accumulation increases autophagic flux, with HAP1 being one of the substrates of UBE3A.^518^ Additionally, genes downstream of IRF are enriched in a UBE3A-deficient mouse model of AS.^518^ A 2025 study revealed that Kv4.2, a voltage-gated potassium channel, is an activity-regulated substrate of UBE3A.^519^ UBE3A binds to Kv4.2 at its N-terminus, ubiquitinating the K103 residue, which leads to activity-induced degradation of Kv4.2.^519^ In an AS mouse model, Kv4.2 protein levels were found to be elevated, and kainic acid-induced Kv4.2 degradation was abolished.^519^

Cabezas syndrome (MIM 300354), a recently characterized X-linked form of syndromic intellectual disability, arises from mutations in the *CUL4B* gene.^520^ Located on chromosome Xq24, the *CUL4B* gene comprises 22 exons and encodes cullin4B, a component of the cullin-RING Ub ligase family, which plays a vital role in the regulation of cellular protein degradation.^521^ Seizures or epilepsy were observed in 43% of male individuals with hemizygous variants in the *CUL4B* gene linked to Cabezas syndrome.^522^ The mechanisms underlying CUL4B-related epilepsy are likely multifactorial. In some cases, it manifests as symptomatic epilepsy associated with cortical developmental malformations, with the interaction between CUL4B and WDR62—linked to various brain malformations—potentially playing a role.^522^ However, in other cases, epilepsy does not appear to be related to cortical malformations but may instead arise directly from the gene’s impact on neuronal excitability.^522^ Functional studies examining the effects of pathogenic variants identified in patients with seizures are essential to validate this hypothesis.

MJD is a progressive NDD characterized by extensive neuronal degeneration in multiple brain regions, including the cerebellum.^347^ MJD arises from a mutation in the *ATXN3* gene, leading to an expanded polyglutamine (polyQ) tract in the ataxin-3 protein.^347^ This polyQ expansion induces protein misfolding and aggregation, resulting in the accumulation of mutant ataxin-3 protein in ubiquitinated intranuclear inclusions, which selectively accumulate in neurons of specific brain regions.^347,523^ MJD is the most common autosomal-dominant genetic ataxia worldwide.^524^ Research has demonstrated that modulating serotonergic signaling pathways leads to improvements in critical aspects of this NDD, notably in reducing the aggregation of mutant proteins.^524^

Deletion or mutation of *USP7* leads to HAFOUS, a disorder characterized by aggressive behavior, intellectual disability, and developmental delay.^253^ USP7 primarily targets the Ub ligase MDM2, which facilitates p53 ubiquitination and degradation.^525^ By inhibiting MDM2 self-ubiquitination, USP7 stabilizes MDM2, thus enhancing MDM2-mediated p53 degradation.^525^ Therefore, targeting USP7 for inhibition has emerged as a potential strategy for tumor suppression. Neural stem cell-specific deletion of USP7 leads to a smaller brain size, accumulation of p53, and perinatal lethality.^526^ Although deletion of *Trp53* (the murine gene encoding p53) restores brain size in Usp7-deficient mice, it does not improve neonatal survival, indicating that USP7 is crucial for nervous system development through mechanisms beyond p53 regulation.^526^ In 2025, Chen H et al. reported the neuropathogenesis of HAFOUS, highlighting USP7 as a key regulator of the RNA splicing factor Ppil4.^253^ Knockdown of Ppil4 was found to mimic the dendritic spine defects observed in *USP7* deficiency.^253^ Together, USP7 and Ppil4 converge to regulate the RNA splicing program of synaptic genes, thereby playing crucial roles in synapse development.

Stankiewicz–Isidor syndrome (OMIM no. 604450) is a neurodevelopmental disorder with a syndromic phenotype associated with loss-of-function variants in *PSMD12*.^527^ PSMD12 encodes Rpn5, a 52.9-kd protein in the 19S regulatory subunit of the proteasome, which is involved in the deubiquitination of captured substrates.^527^ Its clinical presentation includes brain abnormalities, distinct facial features, ocular anomalies, genital malformations, and skeletal defects, without the hallmark features typically associated with autoinflammatory disorders.^527^ The truncated *PSMD12* variant is distinguished by a distinct array of inflammatory markers resembling those found in patients with PRAAS, also known as CANDLE, microcytic anemia, muscle atrophy, joint contractures, Nakajo–Nishimura syndrome (NNS), panniculitis-induced lipodystrophy syndrome (JMP), and Japanese autoinflammatory syndrome with lipodystrophy (JASL).^527^ As described by Isidor et al. (2022), a subset of patient cells demonstrates significant remodeling of the mTORC1 and mitophagy pathways, coupled with the induction of type I IFN-stimulated genes.^528^

## Therapeutic targets in ubiquitination and deubiquitination

**Fig. 10:**
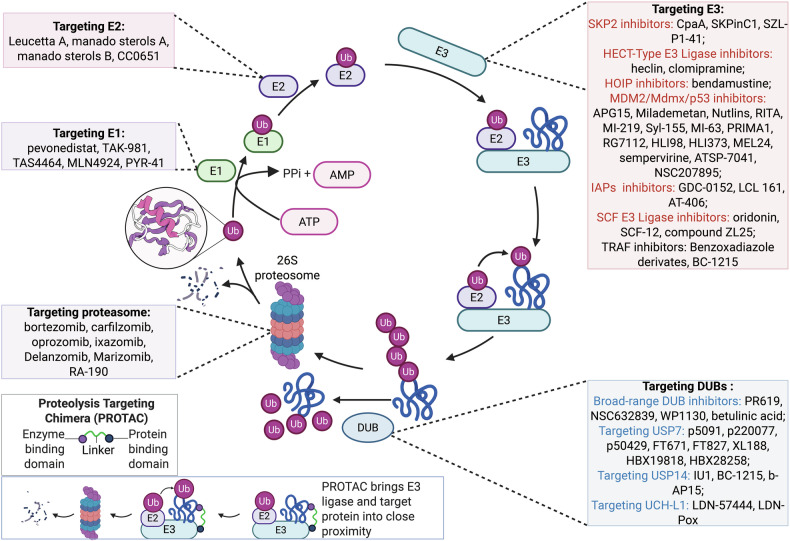
A schematic illustrating the regulation of proteins by the ubiquitin–proteasome system and associated therapeutic approaches, including a depiction of proteolysis-targeting chimeras (PROTACs) as degraders. The elements were obtained from BioRender.com

As previously highlighted, the UPS is essential for protein degradation and the regulation of key cellular functions. Disruptions in the function, expression, or genetic makeup of UPS components are often implicated in the development of diseases, including cancers, NDDs, CVDs, inflammatory diseases, autoinflammatory disorders and developmental defects. As a result, UPS components have emerged as promising therapeutic targets. A growing number of small-molecule inhibitors aimed at various UPS components—such as the proteasome, E1 and E2 enzymes, E3 ligases, and DUBs—are being developed, and their therapeutic efficacy is being progressively evaluated (Fig. 10). Currently, inhibitors of ubiquitination, including carfilzomib and bortezomib, are approved for clinical use in oncology. One promising approach for NDDs is the use of the reversible properties of ubiquitination through DUBs to restore cellular homeostasis. Moreover, the development of PROTACs and molecular glues, which facilitate selective protein degradation via E3 ligase recruitment, represents a rapidly advancing research area.

### E1 inhibitors

As illustrated in Figs. 1, 10, the E1 enzyme activates Ub and transfers it to the E2 enzyme. AMP is essential for the catalytic cascade of Ub activation because it tightly binds to the E1 enzyme. Certain AMP-related compounds can act as E1 inhibitors. Notable sodium adenosulfonate-based E1 inhibitors include MLN7243 (formerly known as TAK243), perazone, ABP1, ABPA3, acetyl-DL-carnitine, TAK-981, ML-792, and MLN4924.^450^ Additionally, adenosine sulfamate analogs such as MLN4924 and MLN7243 have been identified as inhibitors of NAE and Uba1, respectively.^529,530^ MLN7243 targets Uba1, depleting cellular Ub conjugates and demonstrating antitumor activity in primary human xenografts. This drug is currently in phase I trials for advanced solid tumors.^529^ MLN4924 inhibits NAE by forming an irreversible covalent bond with NEDD8 and is in phase III trials for chronic myelomonocytic leukemia (CMML), acute myeloid leukemia (AML), and high-risk myelodysplastic syndrome (MDS).^531^ Among the E1 inhibitors under clinical investigation, pevonedistat (PEV) is the only compound with published clinical trial outcomes. PEV-based combination therapies, such as azacitidine (AZA) and risperidone, have shown efficacy in AML, whereas combinations with paclitaxel and carboplatin have produced clinical benefits in advanced solid tumors (ClinicalTrials.gov: NCT01862328).^532^ A phase 1b trial combining PEV and AZA for refractory/relapsed AML, on the basis of evidence of synergistic preclinical activity, yielded a 50% overall response rate (ORR), including 20 complete remissions (CRs), five CRs with incomplete blood count recovery, and seven partial remissions (PRs), with a median remission duration of 8.3 months. In patients with TP53 mutations, the composite CR/PR rate was 80% (four of five cases) (ClinicalTrials.gov: NCT01814826).^533^ Experimental E1 inhibitors, such as PYR-41 [4-(4-3,5-dioxo-pyrazolidin-1-yl)-benzoic acid ethyl ester], have been developed as irreversible inhibitors that block ubiquitination initiation, prevent p53 degradation and promote p53-dependent apoptotic cell death.^534^ PYR-41 also modulates inflammation by suppressing NF-κB activity and reducing cytokine and chemokine expression. Inhibiting NF-κB via PYR-41 may provide a therapeutic approach for sepsis.^535^

### E2 inhibitors

E2 enzymes facilitate mainly Ub conjugation to substrate proteins, and current research aims to identify inhibitors that interfere with E1–E2 or E2–E3 interactions. For example, Leucetta A selectively disrupts the molecular interplay between UBC13 and UEV1A, effectively antagonizing the formation of their functional heterodimeric complex.^536^ Madosterols A and B, which are isolated from Lissodendoryx fibrosa, are dimeric sulfonated sterol derivatives that competitively inhibit UBC13-UEV1A complex formation through bispecific molecular engagement. These structurally unique metabolites feature twin sterol cores sulfonated at the C-3 and C-24 positions, which are interconnected via a flexible alkyl-bridged side chain architecture.^537^ Moreover, the Cdc34-specific E2 inhibitor CC0651 prevents p27 ubiquitination and degradation, thereby suppressing tumor cell proliferation.^538^ Small-molecule microarray screening has also identified several inhibitors targeting the SUMO E2 enzyme Ubc9.^539^

### E3 inhibitors

The E3 Ub ligase orchestrates coordinated enzymatic cascades by mediating substrate recognition and Ub transfer from E2-conjugating enzymes, which are precharged by the E1-activating enzyme, to complete target protein modification. In oncotherapeutic contexts, pharmacological modulation of E3 ligases serves as a precision strategy to direct Ub conjugation to specific lysine residues on oncoproteins, thereby enabling proteasomal degradation or functional alteration. The multicenter, double-blind phase III study (NCT01097018) assessed perifosine combined with capecitabine versus placebo plus capecitabine in 468 patients with treatment-refractory advanced CRC. At a median follow-up of 6.6 months, the primary endpoint of overall survival (OS) was not significantly different: the median OS was 6.4 months (arm A) vs 6.8 months (arm B) (HR 1.111, 95% CI 0.905–1.365; *p* = 0.315). Subgroup analyses by KRAS status (wild-type: 6.6 vs 6.8 months, HR 1.020, *p* = 0.894; mutant: 5.4 vs 6.9 months, HR 1.192, *p* = 0.238) also revealed no improvement. Despite promising phase II data on the synergistic effects of AKT pathway inhibition, this trial did not demonstrate survival benefits from perifosine in refractory CRC.^540^

A systematic analysis of phase I/II oncology clinical trial data (Table S5), sourced from publicly available repositories (ClinicalTrials.gov, PubChem), highlights the accelerating development of therapeutics targeting E3 Ub ligase–substrate interfaces. These efforts primarily involve two strategies: the use of small-molecule inhibitors that disrupt catalytic domains and the use of molecular glues that stabilize ternary complexes to promote TPD.

### PROTACs

TPD is attracting considerable interest for its potential to modulate “undrugged” and other challenging protein targets.^72^ A key approach facilitating this process involves PROTACs, a class of molecules capable of directing the degradation of these difficult-to-target proteins.^72^ Conventional PROTACs are bifunctional molecules consisting of a ligand for the protein of interest (POI), a linker, and a ligand for an E3 Ub ligase. By simultaneously binding both the POI and the ligase, PROTACs facilitate the ubiquitylation of the POI, resulting in its degradation through the UPS.^541^ After this process, the PROTAC is recycled to target and degrade additional copies of the POI (Fig. 10).^72,541^ PROTACs present distinct advantages over traditional inhibitors, notably their event-driven mechanism, which enables binding to multiple sites on a target protein for degradation, thus broadening the range of accessible targets.^542^ Additionally, their catalytic nature allows prolonged and effective drug action with reduced dosing and frequency, offering the potential to overcome resistance commonly encountered with conventional therapies.^78^ Most PROTACs interact reversibly and noncovalently with the POI, which may result in weaker binding affinities and off-target effects.^543^ In contrast, covalent PROTACs facilitate the formation of the complex required for degradation, transitioning from ternary to binary complex formation, which enhances degradation efficiency.^544,545^ Reversible covalent PROTACs combine dual functionalities by serving as both high-affinity binders of POIs and effective catalysts for POI degradation, acting as both inhibitors and degraders simultaneously.^545^ Recent studies have shown that covalent PROTACs target AR,^546^ STING,^547^ and various other proteins. These covalent PROTACs have demonstrated promising therapeutic potential in treating cancer and other conditions, although none have yet advanced to clinical use.

As previously noted, two leading PROTACs, ARV-471 (currently the only PROTAC in phase III clinical trials) and ARV-110, represent major advancements in PROTAC technology and are undergoing phase III (NCT04072952) and phase II (NCT03888612) clinical trials, respectively.^78,548,549^ Additionally, other drugs currently in phase II trials include ARV-766, which targets AR in prostate cancer (NCT05067140),^550^ and KT-474, which targets IRAK4 in hidradenitis suppurativa and atopic dermatitis (NCTO6058156).^551^ Drugs in phase I/II trials include DT2216, which targets BCL-XL in relapsed or refractory malignancies (NCT04886622)^552^; RNK05047, which targets BTK in diffuse large B-cell lymphoma (NCT05487170)^553^; CFT8634, which targets BRD9 in synovial sarcoma (NCT05355753)^554^; and CFT1946, which targets BRAFV600X in advanced-stage B-RAF V600-positive solid tumors (NCT05668585).^555^ Owing to space constraints, other PROTACs in early-phase trials will not be elaborated here. Notably, among molecular glue drugs, CC-220, which targets IKZF1 in MM, has entered phase III clinical trials (NCT04975997),^556^ and CC-92480, which targets IKZF1/3 in relapsed or refractory MM, is also in phase III (NCT05552976).^557^ The drugs currently in phase III trials are one step away from potential clinical application, making them worthy of continued attention and anticipation.

Notably, PROTACs and molecular glues are increasingly being investigated beyond oncology, with expanding applications in CVDs and NDDs. In cardiac fibrosis, recent advances in PROTAC technology have enabled novel therapeutic strategies. PROTAC degraders that target fibroblast activation protein-α (FAP-α), which is highly expressed in myofibroblasts, have demonstrated selective degradation of FAP-α, suggesting a promising approach for treating resistant fibrotic disorders.^558^ Additionally, PROTACs directed against the platelet-derived growth factor (PDGF)/PDGF receptor (PDGFR) axis are emerging as potential tools to disrupt key fibrotic signaling pathways.^559^ Histone deacetylase 8 (HDAC8), a class I HDAC, is gaining attention as a potential target for cancer, fibrotic diseases, X-linked intellectual disability, and several neuropathological disorders.^560^ Furthermore, in AD, tau undergoes abnormal PTMs and aggregation, with disrupted intracellular degradation pathways contributing to the accumulation of pathological tau. A novel therapeutic approach, TPD, has recently emerged in drug discovery, leveraging bifunctional molecules that bring target proteins to the degradation machinery for clearance. Currently, most tau-TPD proteins are PROTACs, such as C8, T3, C004019, QC-01-175, I3, Peptide 2, TH006, and Keap1-dependent Peptide 1.^460^ In parallel, molecular glue degraders function through the stabilization of neointeractions between target proteins and E3 ligases, promoting ubiquitin-mediated degradation.^561^ There is a growing need for new therapeutic targets based on various pathologies to facilitate the development of PROTACs or molecular glues that can specifically regulate protein homeostasis.

### Proteinase inhibitors

The proteasome is a promising target in disease therapy, particularly in hematologic malignancies. Proteasome inhibitors (PIs), including bortezomib,^562^ carfilzomib,^563^ oprozomib,^564^ and ixazomib,^563^ have been successfully developed for clinical use and have demonstrated significant efficacy (Fig. 10).

PIs are generally well tolerated for clinical use and have minimal side effects. Bortezomib, a first-generation proteasome inhibitor, received approval from the US FDA in May 2003. In 2017, the SWOG S0777 phase III trial confirmed its status as a first-line treatment when it was combined with dexamethasone and lenalidomide (VRD). To date, the VRD regimen, which includes bortezomib, remains the standard first-line therapy for newly diagnosed MM.^565^ Bortezomib, a first-generation proteasome inhibitor, is undergoing clinical evaluation beyond its established myeloma indications, with active trials investigating its therapeutic potential in treating autoimmune hemolysis and colorectal adenocarcinoma (COAD).^566,567^

The FDA approved bortezomib in 2003 for MM, followed by the approval of carfilzomib, a semisynthetic epoxomicin derivative, for refractory MM in 2012. Both drugs target the trypsin-like, caspase-like, and chymotrypsin-like subunits of the 20S proteasome (β1, β2, and β5) and are used in combination therapy with dexamethasone and an immunomodulatory drug.^568^ Carfilzomib is currently undergoing clinical trials for various diseases, including advanced neuroendocrine cancers (NCT02318784), Waldenström’s macroglobulinemia (WM) (NCT01470196), chronic graft-versus-host disease (NCT02491359), antibody-mediated rejection and desensitization (NCT03805178), relapsed WM IST-CAR-531 (NCT01813227), advanced solid tumors (NCT02257476), and autologous hematopoietic stem cell transplantation (NCT01690143).

Oprozomib (ONX0912; PR-047), a next-generation proteasome inhibitor, is a tripeptide analog of carfilzomib. The lead compound PR-047 effectively inhibits the CT-L activity of cCP (β5) and iCP (LMP7).^569^ Notably, it exhibited an absolute bioavailability of up to 39% in preclinical studies conducted in rodents and dogs.^569^ Unlike carfilzomib and bortezomib, which are intravenously administered, oprozomib has improved oral bioavailability, enabling oral delivery. It has demonstrated antitumor efficacy, potency, and selectivity comparable to that of carfilzomib in MM, including activity against MM resistant to bortezomib, dexamethasone, or lenalidomide.^570^ In solid tumors, oprozomib, like carfilzomib, induces apoptosis by increasing the expression of the proapoptotic proteins Bik and Mcl-1.^571^ However, its oral use is associated with unstable pharmacokinetics and frequent gastrointestinal adverse effects, with 84.6% of patients experiencing varying degrees of nausea.^572^

Ixazomib citrate (MLN9708) and its biologically active form, ixazomib (MLN2238), represent the first orally available PIs with enhanced pharmacodynamic and pharmacokinetic characteristics.^573^ Like bortezomib, ixazomib citrate is a boron-containing dipeptide that reversibly binds to the β5 subunit of the proteasome, thereby inhibiting chymotrypsin-like activity.^574^ Compared with bortezomib, ixazomib has demonstrated superior antitumor effects.^575^ When used in combination with lenalidomide and dexamethasone, it significantly improves survival outcomes in MM patients.^576^ Recent findings indicate that ixazomib promotes apoptosis in neuroblastoma cells in vitro and in vivo by suppressing doxorubicin-induced NF-κB signaling, thereby increasing the sensitivity of neuroblastoma cells to doxorubicin-mediated cytotoxicity.^577^ Furthermore, studies have confirmed that cotreatment with ixazomib and the CDK inhibitor dinaciclib is efficacious against HCC. Both in vitro and in vivo assessments demonstrated that this combination induces synergistic apoptosis and inhibits proliferation more effectively than does sorafenib in reducing patient-derived xenografts in murine models.^578^ Patients who are resistant to bortezomib have shown favorable responses to ixazomib.^579^

Other PIs, such as delanzomib and marizomib, are also being evaluated in clinical trials. Marizomib, in particular, has been studied for its potential effects on GB, leveraging its ability to cross the blood‒brain barrier.^580^ A phase 3 trial (NCT02181413) demonstrated that ixazomib maintenance therapy can prolong progression-free survival (PFS) and serve as a viable posttransplant maintenance option in newly diagnosed MM patients. With a median follow-up of 31 months, ixazomib reduced the risk of disease progression or death by 28% relative to placebo (median PFS: 26.5 vs. 21.3 months, *p* = 0.0023).^581^ Delanzomib (CEP-18770), a reversible, orally bioavailable bortezomib analog, has also been studied. Although it can overcome bortezomib-induced peripheral neuropathy, its clinical application is limited due to severe skin toxicity.^582^

### DUB inhibitors

Tightly regulate protein fate through the catalytic editing of Ub modifications, either by trimming PUCs or reversing substrate ubiquitination.^583^ These enzymes serve as critical regulators of genomic fidelity by orchestrating cell cycle checkpoints and suppressing replication stress—mechanisms frequently hijacked during oncogenic transformation.^584^ Consequently, pharmacologic targeting of DUBs has evolved into a precision oncology strategy, with drug candidates spanning pan-DUB modulators to targeted agents with isoform selectivity, several now in clinical evaluation for malignancy-specific vulnerabilities.^585^

Preclinical validation of agents targeting UCH-L1, such as LDN-57444, has demonstrated context-dependent efficacy through the modulation of β-catenin degradation kinetics.^586^ In vitro, VLX1570 inhibits USP14, with reduced activity against UCHL5. Both USP14 and UCHL5, DUBs localized within the 19S proteasome cap, are targeted by VLX1570, resulting in rapid, tumor-specific apoptosis in WM cells resistant to bortezomib or ibrutinib.^587^ Additionally, the *N*-benzyl-2-phenylpyrimidin-4-amine scaffold, represented by the lead compound ML323 (CID 50930790), exhibits submicromolar potency against the USP1/UAF1 DUB complex. In non-small cell lung cancer (NSCLC) models, pharmacodynamic consistency was observed between USP1 inhibition and biomarker modulation, specifically the accumulation of monoubiquitinated PCNA, which was correlated with clonogenic suppression.^588^ The covalent USP2 inhibitor ML364 shows biochemical potency (IC50 of 1.1 μM), with on-target effects, including a reduced Cyclin D1 half-life, G1-phase arrest, and selective antiproliferation. Mechanistically, USP2 inhibition impairs HR repair, aligning with the role of Cyclin D1 in DDR pathway activation.^589^ Vialinin A, a compound derived from traditional Chinese medicine, inhibits the catalytic activity of USP4 and suppresses Th17 cell differentiation. USP4 associates with RORγt and removes K48-linked PUCs, thereby increasing RORγt activity and promoting IL-17A expression. Importantly, the combination of TGF-β and IL-6 facilitates USP4-mediated deubiquitination of RORγt. Increased mRNA levels of USP4 and IL-17, but not of RORγt, were detected in CD4⁺ T cells from patients with rheumatic heart disease, indicating that USP4 could serve as a therapeutic target in Th17-driven autoimmune disorders.^590^

HBX 41,108, a small-molecule inhibitor of USP7 with submicromolar IC_50_, blocks the deubiquitinating activity of USP7. Both in vitro and cellular assays revealed that HBX 41,108 interferes with USP7-dependent p53 deubiquitination, leading to the activation of p53-responsive genes without inducing genotoxic stress or inhibiting the proliferation of cancer cells. The compound also promotes p53-dependent apoptosis in isogenic cancer cell lines with either wild-type or null p53.^591^ Compound 1, a dual USP7 and USP47 inhibitor with moderate potency, elevates p53 protein levels and triggers apoptosis in cancer cells. In vivo evaluation in human xenograft models of MM and B-cell leukemia revealed limited efficacy, likely due to dual inhibition of USP47 and USP7. Subsequent structural analogs of compound 1 have been synthesized to improve its potency, solubility, and metabolic stability. Further optimization may yield preclinical and clinical candidates for treating MM, prostate cancer, and other malignancies.^592^ WP1130, a low-molecular-weight compound derived from a parent structure with Janus-activated kinase 2 (JAK2) inhibitory effects, induces rapid aggregation of K48/K63-linked polyubiquitinated proteins near the nucleus while maintaining 20S proteasome function. As a semiselective DUB modulator, WP1130 inhibits UCH37, USP14, USP5, and USP9x, which are key regulators of survival protein stability and 26S proteasome dynamics. Its oncotherapeutic potential lies in its ability to destabilize cytoprotective proteins and enhance proapoptotic factors, particularly through MCL-1 suppression and activation of the p53 pathway, thereby promoting programmed cell death.^593^ Spautin-1 induces the degradation of Vps34 PI3 kinase complexes by inhibiting the DUBs USP13 and USP10, which specifically target the Beclin1 subunit. This mechanism holds promise for the development of new anticancer therapies.^594^ High-throughput screening revealed a selective small-molecule inhibitor that targets the deubiquitinating activity of USP14. This compound promoted the degradation of proteasome substrates linked to NDDs.^595^ Finally, the diterpenoid derivative 15-oxospiramilactone (S3) promotes mitochondrial fusion and restores mitochondrial function in cells lacking Mfn1 or Mfn2. By targeting the mitochondrion-localized DUB USP30, S3 inhibits its activity, enhancing the nondegradative ubiquitination of Mfn1/2. These findings suggest that S3 may serve as a potential treatment for neurodegeneration.^596^ Therefore, strategies spanning enzymatic inhibition to proteostasis modulation demonstrate translational potential across oncology and neurodegeneration.

## Conclusions and future perspectives

Over the past few decades, our understanding of ubiquitination has expanded from a niche mechanism for protein degradation to a complex and multifaceted regulatory system influencing nearly all aspects of cell physiology and disease pathology.^597^ During ubiquitination, E3 ligases play a critical role in selecting specific E2 enzymes and substrates, as well as in directly attaching Ub to the substrate. However, the precise mechanisms by which E3 ligases recognize particular E2s, select substrates, and identify specific lysines on the substrate remain unclear. To address these questions, further structural and functional studies are essential to elucidate the regulatory mechanisms governing E3 ligases. Additionally, in ubiquitination, E3 ligases regulate the diverse functions of substrates by controlling different types of ubiquitination. For example, MDM2 can promote both the ubiquitination and neddylation of p53.^598,599^ To fully understand the dynamics and complexity of these processes, it is crucial to focus on unraveling the various mechanisms by which E3 ligases assemble ubiquitin chains.

The UPS, as well as its associated signaling axes and ubiquitin-like modifiers, is now recognized as a central node in cell fate determination, stress responses, immune signaling, metabolic balance, and oncogenic transformation.^600^ Despite significant progress, several key issues remain unresolved in the UPS. For example, while K48- and K11-linked ubiquitination are recognized as degradation signals that direct substrates to the 26S proteasome,^12^ the precise mechanisms by which the proteasome recognizes K11-linked ubiquitination and differentiates between various Ub linkages are not yet fully understood. Additionally, the exact length of the Ub chain remains uncertain. Determining the length of Ub chains in cells and understanding how this length influences substrate function are critical. Moreover, it has been demonstrated that a K48-linked Ub chain consisting of four Ub molecules can be degraded by the proteasome^601^; however, why is this specific length? This question extends to other types of Ub linkages as well. These unresolved issues are largely due to technical limitations in detecting PUC lengths. Therefore, the development of more advanced technologies to address these challenges is essential for gaining deeper insights into the UPS.

From the pioneering discovery of the role of Ub in proteolysis to recent insights into nonproteolytic signaling, the field has undergone remarkable progress. Key advances have revealed the diversity of Ub chain linkages (e.g., K48 vs. K63),^602^ the specificity conferred by hundreds of E3 ligases and DUBs, and the intricate crosstalk with core signaling pathways such as the mTOR, PTEN-AKT, Wnt/β-catenin, and Hippo–YAP pathways. This complexity underscores the unique role of ubiquitination as both a master regulator and a context-dependent effector in health and disease.^27^ Furthermore, as targeting the TME has gained prominence in cancer therapy, numerous studies have investigated the role of ubiquitination in tumor immunology. However, the exact functions of ubiquitination in T cells, macrophages, and dendritic cells (DCs) remain poorly understood, especially with respect to immunotherapies targeting ubiquitination, highlighting the need for further exploration and development.

Therapeutically, the UPS has already delivered success stories, most notably in cancer treatment with FDA-approved PIs such as bortezomib and carfilzomib, which have revolutionized the management of MM.^562^ In addition to the proteasome, recent clinical strategies have aimed to selectively target E3 ligases and DUBs or to hijack the Ub machinery via PROTACs and molecular glues for the degradation of previously “undruggable” targets.^603^ These technologies offer unprecedented specificity and tunability, enabling precise degradation of pathogenic proteins while minimizing off-target effects. However, major challenges remain. First, the inherent redundancy and plasticity of the Ub system can obscure therapeutic windows and lead to compensatory resistance mechanisms. Second, selectively targeting individual E3 ligases or DUBs without affecting global proteostasis remains technically demanding. Moreover, context-dependent roles of ubiquitination in different tissues and disease stages demand highly personalized therapeutic approaches. Future research should focus on several promising directions: (1) mapping the Ub landscape with greater temporal and spatial resolution to understand its dynamic regulation in disease; (2) developing tissue-specific delivery systems for UPS-targeted therapies, minimizing systemic toxicity; (3) harnessing synthetic biology tools, including engineered E3 ligases and programmable DUBs, to manipulate protein fate with precision; (4) expanding PROTACs and molecular glue libraries to cover a broader range of disease-associated proteins; (5) Integrating Ub pathway signatures into biomarker panels, facilitating real-time monitoring of disease progression and therapeutic responses.

In conclusion, the field of ubiquitination research stands at a transformative juncture. As we continue to dissect the nuances of this regulatory network, novel therapeutic strategies are emerging that could radically redefine how we treat cancer, neurodegeneration, CVDs, and beyond. Continued interdisciplinary efforts—linking structural biology, pharmacology, genomics, and clinical research—will be crucial for translating the promise of ubiquitin-targeted therapies into real-world clinical success.

## Supplementary information

Supplementary Table S1-5
