# Supplementary material for: Targeting ubiquitination in disease and therapy
Source: Signal Transduct Target Ther. 2025 Dec 23;10:424. doi: 10.1038/s41392-025-02392-8 (PMC12728233; doi:10.1038/s41392-025-02392-8)
Supplement: Supplementary file 1 — Supplementary Table S1-5 [file 41392_2025_2392_MOESM1_ESM.docx]

Supplementary Materials for

**Targeting ubiquitination in disease and therapy**

Xiaojuan Yang, Tian Lan, Buzhe Zhang, Xue Tao, Weili Qi, Kunlin Xie, Yunshi Cai, Chang Liu, Junhong Han, Hong Wu

Correspondence to: [drliuchang@wchscu.cn](mailto:drliuchang@wchscu.cn), [hjunhong@scu.edu.cn](mailto:hjunhong@scu.edu.cn), wuhong@scu.edu.cn

**This PDF file includes:**

Tables S1 to S5

| **Oncogenic traits** | **Ubiquitin enzymes** | **Name** | **Target protein** | **Associated cancers** | **Experimental models** | **Molecular** | **Clinical effects** | **References** |
| --- | --- | --- | --- | --- | --- | --- | --- | --- |
| i. Sustaining proliferative signaling | E3s | Nrdp1 | ErbB3 | Breast cancer | Cell | Degrades HER3 and promotes  tumor proliferation | / | ^1^ |
|  |  | MARCH6 | DHX9 | Primary papillary thyroid  cancers | Cell, subcutaneous tumorigenesis model, human tumor samples | Activates AKT/mTOR signaling  pathway and promotes tumor  proliferation and metastasis | Overexpressed in the tumor tissues | ^2^ |
|  |  | ANKRD9 | IMPDH | Gastric cancer | Cell | Promotes ubiquitination  and proteasomal degradation  of IMPDH to suppress tumor  growth | / | ^3^ |
|  |  | MKRN1 | PTEN | Cervical cancer | Cell, subcutaneous tumorigenesis model, human tumor samples | Ubiquitinates PTEN protein  and promotes cancer proliferation | Overexpression with tumor progression | ^4^ |
|  |  | BCR-ABL | SHIP | Hematopoietic tumors | Cell, SHIP^+/+^ (wild-type [WT]) F2 mice | Promotes SHIP proteasomal  degradation | / | ^5^ |
|  |  | FBX8 | mTOR | Colorectal carcinoma | Cell, subcutaneous xenograft tumor model, pulmonary metastasis model, patient tissues | Targets mTOR for degradation | Low expression with poor overall survival of patients |  |
|  |  | SAG | PHLPP1/DEPTOR | Prostate cancer | Cell, the *Sag* *^fl/fl^* conditional KO mouse model/ Pb4-Cre and *Pten* *^fl/fl^* (strain B6.129S4-Pten^tm1Hwu^/J) mice, human prostate cancer tissues | Activates the PI3K/AKT/mTOR  axis | Increased expression from normal to benign, then to malignant lesions, peaking  In the metastatic tumors | ^6^ |
|  |  | RFP2 | AKT/MDM2 | Multiple myeloma | Cell | Degrades AKT and MDM2 | / | ^7^ |
|  |  | BRCA1 | AKT | Breast cancer | Cell | Ubiquitinates and directly  degrades phosphorylated AKT | / | ^8^ |
|  |  | CHIP | AKT | Cervical cancer | Cell | Ubiquitination-dependent degradation of p-AKT (Thr308/Ser473) | / | ^9^ |
|  |  | SKP2 | AKT | Breast cancer | Cell, *MMTV-Neu* transgenic mice, breast cancer patients | Promotes tumor occurrence  and metastasis | Upregulation; promoting breast cancer progression | ^10^ |
|  |  | TRAF4 | AKT | Lung cancer | Cell, xenograft mouse model, human lung cancer tissue | Promotes tumorigenesis | A critical molecule in lung cancer development | ^11^ |
|  |  | FBXL18 | AKT | Glioma | Cell, clinical specimens | Promotes tumor proliferation  and development | Upregulation; high-grade | ^12^ |
|  | DUBs | USP17/USP4 | PDFGRβ | Osteosarcoma | Cell | Promotes aberrant STAT3  transcription | / | ^13^ |
|  |  | USP15 | ERα | Breast cancer | Cell, ER-positive xenograft in mice | Blocks the ubiquitination  and degradation of ERα | / | ^14^ |
|  |  | USP4 | TAK1 | Esophageal squamous cell  carcinoma | Cell, subcutaneous xenograft tumor model, human tissues | Stabilizes the TAK1 protein level | Poor overall survival | ^15^ |
|  |  | USP7 | ERα | Breast cancer | Cell, subcutaneous xenograft tumor model, patient tissues | Deubiquitinates ERα and pro-  motes tumor proliferation | An independent poor prognostic factor | ^16^ |
|  |  | Ataxin-3 | PTEN | Lung cancer | Cell | Restricts PTEN transcription | / | ^17^ |
|  |  | USP13 | PTEN | Breast cancer | Cell, subcutaneous xenograft tumor model, patient tissues | Deubiquitinates PTEN | Downregulation in human breast cancer | ^18^ |
|  |  | USP10 | PTEN | Lung cancer/ hepatocellular  carcinoma | Cell, subcutaneous xenograft tumor model, patient tissues | Upregulates PTEN and  suppresses tumor progression | Downregulation in tumor | ^19,20^ |
|  |  | OTUD3 | PTEN | Breast cancer | Cell, subcutaneous xenograft tumor model, *OTUD3* transgenic mice, patient tissues | Upregulates PTEN and sup-  presses tumorigenesis | Downregulation | ^21^ |
|  |  | USP46 | PHLPP1 | Colon cancer | Cell, subcutaneous xenograft tumor model, | Functions as a tumor suppressor by controlling PHLPP-  dependent attenuation of AKT  signaling | Decreased expression | ^22^ |
|  |  | USP1 | PHLPP1 | Lung cancer | Cell | Regulates AKT phosphorylation by modulating the stability  of PHLPP1 | / | ^23^ |
|  |  | USP21 | MEK2 | HCC | Cell, subcutaneous xenograft tumor model | Maintains MEK2 stability  and activates ERK signaling | / | ^24^ |
|  |  | USP12/WRD48 | PHLPP1 | Colon cancer | Cell | Suppresses AKT-dependent cell  survival signaling by stabilizing  PHLPP1 | / | ^25^ |
| ii. Evading growth suppressors | E3s | TRIM28 | p53 | Lung cancer | Cell, subcutaneous xenograft tumor model, patients and lung tissue samples | Regulates ubiquitin ligases RLIM and MDM2 to target the p53 | Highly expressed in patients | ^26^ |
|  |  | SKP2 | NBS1 | Multiple cancers | Cell | Promotes tumor development | / | ^27^ |
|  |  | CUL1 | CHK2 | Osteosarcoma | Cell | Induce apoptosis | / | ^28^ |
|  | DUBs | USP10 | p53 | Colon cancer/ renal cell carcinoma | Cell | Inhibits tumor development | / | ^29^ |
|  |  | USP7 | p53 | Gastric cancer | Cell, subcutaneous xenograft tumor model, patient tissue samples | Inhibits tumor development | Tumor suppressor | ^30^ |
| iii: Resisting cell death | E3s | IBRDC2 | Bax | Colorectal cancer | Cell | Promotes apoptosis | / | ^31^ |
|  |  | TRIM31 | NLRP3 | Colitis-associated cancer | Cell, with C57BL/6J  with TRIM31^−/−^ mice | Promotes NLRP3 inflammasome | / | ^32,33^ |
|  |  | CUL3 | BECN1 | Breast cancer/Ovarian cancer | Cell, subcutaneous xenograft tumor model, patient tissue samples | Inhibit autophagy and  promotes tumor development | Poor prognosis | ^34^ |
|  | DUBs | USP22 | RIPK3 | Colorectal cancer | Cell | Promotes necroptosis | / | ^35^ |
|  |  | USP24 | GSDMB | Bladder cancer | Cell, subcutaneous xenograft tumor model, patient tissue samples | Inhibits pyroptosis | Upregulation in patient tissues | ^36^ |
|  |  | USP48 | GSDME | Pancreatic cancer | Cell, subcutaneous xenograft tumor model, patient tissue samples | Promotes pyroptosis | / | ^37^ |
|  |  | OTUB1 | GPX4 | Gastric cancer | Cell, tail veins of nude mice, tissue specimens | Inhibits ferroptosis and promotes gastric cancer metastasis | / | ^38^ |
| iv: Enabling replicative immortality | E3s | Rlim | TRF1 | Renal cell carcinoma | Cell | Binds a region between the homodimerization domain and the Myb-like motif of TRF1  to promote  tumor growth | / | ^39^ |
|  |  | FBX4 | PIN2/TRF1 | Fibrosarcoma | Cell | Promote  tumor growth | / | ^40^ |
|  |  | β-TrCP1 | TRF1 | Osteosarcoma | Cell | Promotes tumor growth | / | ^41^ |
|  |  | FBW7 | TPP1 | Lung cancer | Cell | Resistance to senescence and fibrosis | / | ^42^ |
|  |  | Siah1 | TRF2 | Colorectal cancer | Cell | Pro-  motes tumor proliferation | / | ^43^ |
| v: Inducing angiogenesis | E3s | Siah2 | NRF-1 | Breast cancer | Cell, xenograft tumorigenesis, patient tissue samples | Decreased activity of Siah2  and promotes cancer development | / | ^44^ |
|  |  | RNF8 | Tpp1 | / | Cell | Rnf8-mediated Tpp1 ubiquitylation and localization to telomeres | / | ^45^ |
| vi:  Activating invasion and metastasis | E3s | FBXW2 | β-catenin /SKP2/EGFR | Lung cancer/ Prostate cancer | Cell, tail veins of nude mice/ subcutaneous xenograft tumor model, patients and clinical cancer specimens | Promotes ubiquitination  and degradation of oncogenic  proteins and inhibits tumor  migration, invasion, and  metastasis | Decreased expression linked to poor overall survival and promotes metastasis | ^46–48^ |

Abbreviations: HCC, hepatocellular carcinoma.

**Table S1.**

**E3 ligases, and DUBs in the regulation of core oncogenic traits.**

| **Genomic and epigenetic regulation** | **Ubiquitin enzymes** | **Name** | **Target protein** | **Associated cancers** | **Experimental models** | **Molecular** | **Clinical effects** | **References** |
| --- | --- | --- | --- | --- | --- | --- | --- | --- |
| i:  Genome instability and mutation | E3s | RNF126 | MRE11 | Triple-negative breast cancer | Cell, C57BL/6 *Rnf126* ^fl/fl^ conditional knockout mice/ Intracranial mouse xenografts | Confers resistance  to radio-  therapy | **/** | ^49^ |
|  |  | Trim25 | Ku80 | Nasopharyngeal carcinoma | Cell, subcutaneous xenograft tumor model, patients and clinical cancer specimens | Inhibiting DNA repair via non-homologous end joining | **/** | ^50^ |
|  |  | MDM2 | DICER | Breast cancer | Cell, xenograft tumor model, patients and clinical cancer specimens | Impairs DDR and promotes cancer progression | Associated with cancer progression | ^51^ |
|  | DUBs | USP44 | TRIM25 | Nasopharyngeal carcinoma | Cell, subcutaneous xenograft tumor model, patients and clinical cancer specimens | Inhibiting DNA repair via non-homologous end joining | **/** | ^50^ |
|  |  | USP7 | SAMHD1 | Colonic and lung adenocarcinomas/ thyroid carcinoma | Cell, subcutaneous xenograft tumor model, patients and clinical cancer specimens | Repairs DNA damage induced by ROS or genotoxic insults | Highly expressed in multiple cancer tissues | ^52^ |
| ii:  Unlock surface plasticity | E3s | MDM2 | p53 | Dedifferentiated liposarcoma | Cell, xenograft-bearing mice, patients | Targets tumor suppressor p53 | Multiple early-phase clinical trials with MDM2 pathway inhibition | ^53^ |
| iii：Nonmutation epigenetic reprogramming | E2 | RAD6A | H2B | Esophageal squamous cell carcinoma | Cell, subcutaneous xenograft tumor model, patients and clinical cancer specimens | Promotes the proliferation of cancer cells | Poor prognosis | ^54^ |
|  | E3s | RNF180 | DNMT1/ DNMT3A | Gastric cancer | Cell, subcutaneous xenograft tumor model, patients and clinical cancer specimens | Suppress the proliferation and metastasis of cancer cells | / | ^55,56^ |
|  |  | DDB1-Cul4A | H2A | Osteosarcoma | Cell, subcutaneous xenograft tumor model | Inhibits osteosarcoma progression | / | ^57^ |
|  |  | SCF^FBW7^ | Brg1 | Gastric cancer | Cell, tail veins of nude mice, patients and clinical cancer specimens | Inhibits cancer metastasis | Negatively associated with tumor progression and poor outcome | ^58^ |
|  |  | SCF | ARID1A | HCC | Cell, AKT/NRAS-driven liver tumor development in mice | Enhances the growth of cancer cells in vitro and tumor growth in vivo | / | ^59^ |
|  |  | CHIP | Ino80 | Colorectal cancer | Cell | Achieves efective DNA replication | / | ^60^ |
|  | DUBs | USP22 | H2A | Osteosarcoma | Cell, subcutaneous xenograft tumor model | Promotes the progress of osteosarcoma | / | ^57^ |
|  |  | USP7 | FBP1 | Pancreatic cancer | Cell, subcutaneous xenograft tumor model | Increases the sensitivity of pancreatic cancer to PARP inhibitors | / | ^61^ |
|  |  | OTUD6A | Brg1/AR | Prostate cancer | Cell, subcutaneous xenograft tumor and PDX mice model/ *Pten*-floxed mice and Probasin-Cre mice, patients and clinical cancer specimens | Promotes tumorigenesis | Highly expressed and correlated with the recurrence risk and poor survival | ^62^ |

Abbreviations: HCC, hepatocellular carcinoma; PDX, patient-derived xenograft.

**Table S2.**

**E2, E3 ligases, and DUBs in the processes of genomic and epigenetic regulation.**

| **Tumor microenvironment and immune regulation** | **Ubiquitin enzymes** | **Name** | **Target protein** | **Associated cancers** | **Experimental models** | **Molecular** | **Clinical effects** | **References** |
| --- | --- | --- | --- | --- | --- | --- | --- | --- |
| i:  Tumor-promoting inflammation | E3s | BICP0 | TRAF6 | Cervical cancer | Cell | Promotes K48-ubiquitination and promotes the immune evasion | **/** | ^63^ |
|  |  | TRIM22 | NOD2/NF-κB pathway | Endometrial cancer | Cell, subcutaneous xenograft tumor model, patients and clinical cancer specimens | Inhibits tumor progression | Downregulated expression and malignant transformation | ^64^ |
|  |  | TRAF3 | p65-p50 | Gastric cancer | Cell, NOD1-intact and NOD1-deficient mice | Inhibits tumor proliferation | / | ^65^ |
|  |  | HOIP | NEMO | Lymphoma/Breast cancer | Cell, tissue-specific HOIP transgenic mice/ specific double kknockout / inducible double knockout mouse model | Enhances NF-κB activation/Promotes breast cancer development | / | ^66,67^ |
|  |  | OTULIN | M1-linked polyubiquitin signaing | HCC | Cell, The *Otulin*^del/flox^ and *Otulin*-*Rosa26*-Cre-ERT2 mice, human subjects | Promotes tumor growth | OTULIN-related autoinflammatory syndrome may develop liver disease | ^68^ |
|  | DUBs | USP 7 | MDM2 | Breast cancer/prostate cancer | Cell, subcutaneous xenograft tumor model | Impairs DDR and promotes cancer progression | / | ^51^ |
| ii:  Avoiding immune destruction | E3s | SPOP | PD-L1 | Prostate cancer/ colorectal cancer/ lung cancer/ovarian cancer | Cell, subcutaneous xenograft tumor model | Promotes degradation of PD-L1 | / | ^69,70^ |
|  |  | FBW7 | NFAT1 | Metastatic renal cell carcinoma | Cell, subcutaneous xenograft tumor model, patients and clinical cancer specimens | Reduces the expression of PD-L1 by down-regulating NFAT1 | / | ^71^ |
|  |  | FBXO38 | PD-1 | Melanoma/colorectal carcinoma/HCC | Cell, *Fbxo38^flox/flox^* mice, human cancer samples | Regulates PD-1 on the cell surface through the polyubiquitination linked with K48 | Low expression in tumor | ^72^ |
|  |  | FBXO22 | PD-L1 | Lung cancer/colon cancer/breast cancer | Cell | Increases their sensitivity to DNA-damaging therapies | / | ^73^ |
|  |  | NEDD4 | PD-L1 | Urothelial carcinoma | Cell, patient-derived xenograft model, orthotopic tumor model | Catalyzed K48-linked polyubiquitination of PD-L1 | / | ^74^ |
|  |  | Cbl-b | TCR | Lymphoma | Cell, C57BL/6 mice, OT-I and OT-II mice | Downregulates TCR expression | / | ^75^ |
|  |  | HERC2 | JAK2/STAT3 pathway | HCC | Cell, hepatocyte-specific HERC2 knockout mice, human clinical samples | Promotes cancer stemnessand immune escape | Poor prognosis | ^76^ |
|  |  | RNF31 | YAP | Triple-negative breast cancer | Cell, xenograft mouse model/ *vivo* metastasis assay | Represses cell progression and immune evasion | / | ^77^ |
|  |  | RNF5 | PTEN | Pancreatic ductal adenocarcinoma | Cell, xenograft and orthotopic mouse model, patient tissue microarray | Accelerates pancreatic tumor growth | Reduced overall survival | ^78^ |
|  |  | A20 | Snail1 | Breast cancer | Cell, tail veins of nude mice/ orthotopic tumor model, human cancer tissue microarray | Multi-monoubiquitylation of Snail1 and promotes tumor metastasis | Overexpressed in aggressive basal-like breast cancers | ^79^ |
|  |  | TRAF6 | CTLA-4 | Melanoma | Cell, subcutaneous xenograft tumor model | Improvement of T-cell-based immunotherapies | / | ^80^ |
|  | DUBs | USP7 | PD-L1 | Gastric cancer | Cell, subcutaneous xenograft tumor model | Stabilize PD-L1 | / | ^81^ |
|  |  | USP22 | CD274 | Liver cancer/lung cancer | Cell, NOD-Prkd^cem26Cd52^Il2rg^em26Cd22 C57BL/6 mice^, C57BL/6 mice/subcutaneous xenograft tumor model, lung cancer samples | Stabilize PD-L1/ enhanced immunosuppression | Improve the efficiency of cancer treatments based on ICBT | ^82,83^ |
|  |  | CSN5 | PD-L1 | Breast cancer | Cell, orthotopic tumor model | Deubiquitinates PD-L1 protein | / | ^84^ |
|  |  | USP8 | PD-L1 | Pancreatic cancer | Cell, subcutaneous/orthotopic tumor model, patient samples | Deubiquitinates PD-L1 | High expression in tumor tissues | ^85^ |
|  |  | USP14 | IDO1 | Colonic adenocarcinoma | Cell, ubcutaneous xenograft tumor model, patient tissue samples | Stabilizes IDO1 and reduces anti-PD-1 responsiveness | Independent prognostic factor | ^86^ |
|  |  | USP12 | PPM1B | Lung cancer | Cell, subcutaneous xenograft tumor model, lung cancer samples | Deubiquitinates PPM1B | Downregulation in tumor tissues | ^87^ |
|  |  | USP9X | PD-L1 | Oral squamous cell carcinoma | Cell | Stabilize its protein expression | / | ^88^ |
|  |  | USP3 | SUZ12 | Gastric carcinoma | Cell, tail veins of nude mice, tissue specimens | Stabilizes SUZ12 to promote EMT | Tumor progression and poor prognosis | ^89^ |
|  |  | USP15 | TGF-β I | Glioblastoma | Cell, patient-derived samples, tissue microarray | Enhances the tumorigenic efect of TGF-β | USP15 is targeted for amplification in cancer | ^90^ |
|  |  | USP4 | TGF-β I | Breast cancer | Cell | Maintains the stability of TGF- βRI | / | ^91^ |
|  |  | USP27X | Snail1 | Breast cancer/Pancreatic cancer | Cell, PDX, breast tumors subcutaneous xenograft tumor model /tail veins of nude mice | Maintains the stability of Snail1 | / | ^92^ |
|  |  | DUB3 | Snail1 | Breast cancer | Cell, tail veins of nude mice/  spontaneous metastatic model/ orthotopic tumor model, patient samples | Snail1 stabilization | Developing distant metastasis and poor survival | ^93^ |
| iii:  Polymorphic microbiomes | E3s | HECTD2 | EHMT2 | Colorectal cancer | Cell, intraperitoneally injected into mice | Promotes proteasomal degradation of EHMT2 | / | ^94^ |

Abbreviations: DDR, DNA damage repair; HCC, hepatocellular carcinoma; ICBT, immune checkpoint blockade therapy; PDX, patient-derived xenografted.

**Table S3.**

**E3 ligases and DUBs in the processes of the tumor microenvironment and immune regulation.**

| **Reprogramming energy metabolism** | **Ubiquitin enzymes** | **Name** | **Target protein** | **Associated cancers** | **Experimental models** | **Molecular** | **Clinical effects** | **References** |
| --- | --- | --- | --- | --- | --- | --- | --- | --- |
| Reprogramming energy  metabolism | E3s | TRIM36 | HK2 | Prostate cancer | Cell, subcutaneous xenograft tumor model | Inhibits the neuroendocrine differentiation of prostate cancer | / | ^95^ |
|  |  | MARCH8 | HK2 | Colorectal cancer | Cell, subcutaneous xenograft tumor model, patient-derived samples | Inhibits glycolysis | Beneficial prognostic marker | ^96^ |
|  |  | FBW7 | c-Myc | OSCC | Cell, OSCC xenograft model, clinical tissue sample collections | Inhibits oral squamous cell  carcinoma | / | ^97^ |
|  |  | STUB1 | PKM2 | Colorectal cancer | Cell, subcutaneous xenograft tumor model /tail veins of nude mice, human tumor tissues | Inhibits the progress of colorectal cancer | / | ^98^ |
|  |  | TRIM29 | PKM2 | Colorectal cancer | Cell, subcutaneous xenograft tumor model /tail veins of nude mice, human colorectal cancer tissue sample | Promotes colorectal cancer  carcinogenesis | Upregulation in tumor | ^99^ |
|  |  | TRIM35 | PKM2 | Breast cancer | Cell, subcutaneously injected into the mammary fat pads of the mice, patient samples | Inhibits the malignant behavior of breast cancer | Downregulation in tumor tissues and with unfavorable survival in patients | ^100^ |
|  |  | Trib1-COP1 | ACC1 | Myeloid leukemia | Cell, MLL-AF9–induced AML, BCR-ABL–induced CML | Promotes the occurrence  of myeloid leukemia | ACC1 is downregulated in human AML | ^101^ |
|  |  | FBXW7β | FASN | Colorectal cancer | Cell, subcutaneous DLD-1 xenograft model/ PDX model, patients and tissue samples | Promotes the growth of colorectal cancer | High levels of FASN correlate with poor survival | ^102^ |
|  |  | TRIM21 | GAC | Non-small cell lung cancer | Cell, subcutaneous xenograft tumor model | Promotes the occurrence  Of cancer | / | ^103^ |
|  | DUBs | USP7 | HK2 | Gastric cancer | Cell, subcutaneous xenograft tumor model | Promote the progression of cancer | / | ^104^ |
|  |  | OTUB1 | C-Myc | Breast cancer | Cell, subcutaneous xenograft tumor model, patient samples | Promotes breast tumorigenesis | High expression and linked to poor prognosis | ^105^ |
|  |  | USP7 | C-Abl | Non-small-cell lung cancer | Cell, subcutaneous xenograft tumor model | Promotes glycolysis and survival  of cancer  cells | / | ^106^ |
|  |  | TRAP1 | PFK1 | Colorectal cancer | Cell, tumor specimens | Enhances Warburg metabolism | TRAP1 is involved in the glycolytic phenotype | ^107^ |
|  |  | DDX39B | PKM2 | Colorectal cancer | Cell, subcutaneous xenograft tumor model /tail veins of nude mice, human tumor tissues | Promotes the progress of  cancer | Upregulation of DDX39B correlates with aggressive phenotypes and poor prognosis | ^98^ |
|  |  | OTUB2 | PKM2 | Colorectal cancer | Cell, subcutaneous xenograft tumor model, human tumor tissues | Exacerbates the progression  of colorectal cancer by promoting PKM2 activity and glycolysis | Upregulation in cancer tissues and correlated with tumorigenesis and tumor metabolism | ^108^ |
|  |  | PSMD14 | PKM2 | Ovarian cancer | Cell, subcutaneous xenograft tumor model, patients and tissue samples | Promotes ovarian cancer  progression by decreasing  enzymatic activity of PKM2 | Overexpression and associated with ovarian cancer progression | ^109^ |
|  |  | USP35 | PKM2 | HCC | Cell, subcutaneous xenograft tumor model, patients and tissue samples | Promotes HCC progression | Upregulation in HCC and predicts poor prognosis | ^110^ |
|  |  | USP15 | glutamine synthetase | Multiple myeloma | Cell | Promotes amino acid metabolism | / | ^111^ |
|  |  | USP22 | PPARγ | HCC | Cell, subcutaneous xenograft tumor model, patients and tissue samples | Promotes tumorigenesis | High expression in tumor tissues | ^112^ |

Abbreviations: HCC, hepatocellular carcinoma; OSCC, oral squamous cell carcinoma; PDX: patient-derived xenograft.

**Table S4.**

**E3 ligases and DUBs in the processes of the reprogramming energy metabolism.**

| **Therapeutic modalities** | **Target** | **Name** | **Phase** | **Trial details** | **Molecular mechanisms** | **Type diseases** | **Reported data** |
| --- | --- | --- | --- | --- | --- | --- | --- |
| Small-molecule inhibitors | Uba1 | MLN7243 | 1 | NCT02045095 | Inhibitor of ubiquitin-activating enzyme | Advanced solid tumors | No |
|  | SAE | TAK-981 | 1/2 | NCT04074330 ^113^ | Activates IFN1-dependent macrophages and NK cells, enhancing phagocytosis and cytotoxicity | Adults with relapsed or refractory CD20-positive non-hodgkin lymphoma | Antitumor activity in combination with tumor-targeting monoclonal antibodies such as rituximab, mezagitamab or pembrolizumab |
|  |  |  | 2 | NCT03648372 ^113^ |  | Advanced solid tumors |  |
|  |  |  | 1b | NCT04776018 ^113^ |  | Adults with relapsed or refractory multiple myeloma |  |
|  |  |  | 2 | NCT04381650 ^113^ |  | Advanced or metastatic solid tumors |  |
|  | NEDD8 | Pevonedistat | 1/2 | **Multiple myeloma:**  NCT03770260,  NCT00722488 ^114^;  **Myeloid leukemia:**  NCT04712942, NCT03814005, NCT02610777, NCT02782468 ^115^, NCT00911066 ^114,116^, NCT03459859;  **Lymphoblastic leukemia:**  NCT03349281;  **Myelogenous leukemia:**  NCT01814826 ^114,117^;  **Acute myeloid leukemia and myelodysplastic syndromes:**  NCT00677170 ^118^;  **Advanced solid tumors:** NCT03057366 ^116^, NCT02122770 ^119^;  **Advanced solid neoplasm:**  NCT03486314 ^120^;  **Mesothelioma:** NCT03319537;  **Solid neoplasm:**  NCT03330106 ^121^;  **Solid tumors:** NCT01862328 ^114^;  **Nonhematologic malignancies:**  NCT00677170 ^114,122^;  **Metastatic melanoma:**  NCT01011530 ^114,123^ | Covalently binds with NEDD8 to form a pevonedistat-NEDD8 adduct that prevents NEDD8 conjugation to CRLs and thus results in CRL substrate accumulation and apoptotic cell death | Hematological malignancies or  solid tumors | Consistent pharmacokinetics (solid/hematologic cancers); Body surface area-adjusted dosing-dosing ↓ exposure variability, no drug-drug interaction with carboplatin/paclitaxel/azacitidine/gemcitabine/docetaxel ^114,116^;  The pharmacokinetic and safety profiles of pevonedistat plus azacitidine in East Asian patients ^115^;  Pevonedistat (≤50 mg/m²) demonstrated no QT prolongation ^121^; Coadministration of pevonedistat with rifampin, a strong enzyme inducer, did not significantly affect pevonedistat's systemic exposure ^120^; Minor contributions of CYP3A/P-gp to pevonedistat clearance ^119^; Pevonedistat-NEDD8 adducts were found in bone marrow aspirates, confirming target engagement. Three lymphoma patients had partial responses, and 30 patients achieved stable disease ^124^; |
|  |  |  |  |  |  |  |  |
|  |  |  |  |  |  |  |  |
|  |  |  |  |  |  |  |  |
|  |  |  |  |  |  |  |  |
|  |  |  |  |  |  |  |  |
|  |  |  |  |  |  |  |  |
|  |  |  |  |  |  |  |  |
|  |  |  |  |  |  |  |  |
|  |  |  |  |  |  |  |  |
|  |  |  |  |  |  |  |  |
|  |  |  |  |  |  |  |  |
|  |  |  |  |  |  |  |  |
|  |  |  |  |  |  |  |  |
|  |  |  |  |  |  |  |  |
|  |  |  |  |  |  |  |  |
|  |  |  |  |  |  |  |  |
|  | MDM2 | APG115 | 1 | NCT02935907 | APG-115 increases p53 and p21 overexpression, activates p53 - mediated apoptosis in tumor cells retaining wild-type p53 | Solid tumor or lymphoma | No |
|  |  | Milademetan | 1 | NCT01877382 ^125^  NCT03671564  NCT03614455 | Oral MDM2 inhibitor | Advanced Liposarcoma, Solid Tumors; Lymphomas;  Myeloid leukemia; Healthy participants | **Advanced Liposarcoma, Solid Tumors:** N = 107, DCR: 45.8%, mPFS: 4.0 months ^125^. |
|  |  | HDM201 | 1 | NCT02143635  NCT02343172 | p53-MDM2 inhibitor | Advanced tumors that are TP53wt; | **Well-differentiated or dedifferentiated liposarcoma:**  Of 74 enrolled patients, 3 achieved partial response and 38 stable disease ^126^; |
|  |  | AMG 232 | 1;  1b/2a; 1b | NCT01723020  NCT02110355  NCT02016729 ^127^ | MDM2 inhibition | Advanced solid tumors or multiple myeloma; Metastatic melanoma;  Acute myeloid leukemia ^127^ | Response rates differed by TP53 status: 31% (4/13) in wild-type vs. 0% (0/3) in mutant patients ^127^ |
|  |  | RG7112 | 1 | NCT01677780  NCT00559533  NCT00623870  NCT01143740  NCT01164033 ^128^  NCT01605526 | MDM2 antagonist; Non-genotoxic oral p53 activator | Advanced solid tumors; Hematologic neoplasms; Liposarcomas prior to debulking surgery; Advanced solid tumors ^128^; Soft tissue sarcoma | Short-term high-dose daily use (3-5 days) provided better drug exposure and pharmacodynamics responses than weekly low-dose regimens (20 days) ^128^ |
|  |  | SAR405838 | 1 | NCT01636479 ^129^  NCT01985191 ^130^ | HDM2 antagonist | Solid tumours; | The de-differentiated liposarcoma MTD cohort demonstrated 56% stable disease (best response) with 32% 3-month progression-free rate ^129^; 24 efficacy-evaluable patients, DCR: 67% (1 PR + 15 SD) ^130^ |
|  |  | RG7388 | 1 | NCT02670044  NCT03362723  NCT02828930  NCT01773408 ^131^  NCT01462175 ^132^  NCT01901172 | p53-MDM2 inhibitors | Relapsed or refractory acute myeloid leukemia; Solid tumors;  Acute myelogenous leukemia ;  Advanced malignancies except leukemia ^132^；Solid tumors | TP53 wild-type patients exhibited higher composite CR rates with cytarabine combination (35.6%) versus monotherapy (18.9%) ^131^; SD was the best response in 30.6% of patients, with 2 sarcoma cases demonstrating exceptional durability (>600 days) ^132^ |
|  |  | CGM097 | 1 | NCT01760525 ^133^ | Inhibits the p53-HDM2 interaction leading to downstream p53 activation | Advanced solid tumors | DCR: 39% (1 PR +19 SD) correlated with extended therapy: 20 patients (＞16 weeks) including 8 sustaining ＞32 weeks ^133^ |
|  | IAPs | LCl161 | 1/2 | NCT02890069  NCT03111992  NCT01240655  NCT01968915  NCT01617668  NCT01098838 ^134^ | cIAP1/2 antagonist (oral inhibitor of apoptosis proteins inhibitor) | Relapsed and/or refractory multiple myeloma; Advanced solid tumors; Triple negative breast cancer; Advanced solid tumors ^134^ | No patient had an objective response ^134^ |
|  |  | AT-406 | 1/2 | NCT04122625  NCT03871959 ^135^  NCT02022098 ^136^  NCT03270176  NCT01078649 ^137^ | IAP antagonist | Solid tumor; Colorectal cancer; squamous cell carcinoma of the head and neck; Advanced or metastatic NSCLC; Advanced solid tumors and lymphomas | Reduced cIAP1 in colorectal models ^135^; Evidence for the tolinapant combined with FOLFOX in poor prognosis MSS colorectal cancer with elevated cIAP1/2 expression ^135^; Locoregional control at 18 months was achieved in 54% of Debio 1143 patients vs 33% in the placebo group (p=0.026) ^136^;  Five patients (17%) had stable disease as the best response ^137^ |
|  |  | Birinapant | 1b | NCT01940172 | IAP antagonist | Relapsed ovarian cancer | No |
|  |  | APG-1387 | 1 | NCT03386526 | IAP antagonist | Advanced solid tumors or hematologic malignancies | No |
| Small molecules targeting protein degradation | UCHL5 and USP14 | VLX1570 | 1/2 | NCT02372240 | VLX1570 induced polyubiquitinated protein accumulation and increased Grp78/Bip expression in ALL cells | Myeloma | No |
|  | USP1 | KSQ-4279 | 1 | NCT05240898 | USP1 inhibitor | Advanced solid tumors | No |
|  | CUL4-DDB1-  CRBM-RBX1 E3  complex | CC-90009 | 1/2 | NCT04336982 ^138^ | GSPT1-selective cereblon E3 ligase modulator | Acute myeloid leukemia | CC-90009 depletion of GSPT1 induces rapid AML apoptosis, reducing engraftment and LSCs in xenografts of 35 AML samples |
|  | MDM2 | JNJ-26854165 | 1 | NCT00676910 | MDM2 inhibitor | Advanced stage or refractory solid tumors | No |
|  |  | AMG-232 | 1 | NCT03041688 | MDM2 inhibitor | AML | No |
|  |  | ALRN-6924 | 1 | NCT03654716 | Dual MDM2/​MDMX inhibitor | Pediatric cancer | No |
|  |  | DS-3032b | 1 | NCT02579824 | Oral MDM2 inhibitor | Relapsed and/​or refractory multiple myeloma | No |
|  |  | RO6839921 | 1 | NCT02098967 ^139,140^ | An MDM2 antagonist | Advanced solid tumors; AML | **Advanced solid tumors:** Reduced PK variability with a safety profile similar to oral idasanutlin ^139^;  **AML:** The composite response rate was 7.7%, with antileukemic activity in 11 patients (42% DCR), 10 of whom were TP53 wild-type ^139,140^ |
|  |  | BI907828 | 1 | NCT03449381  NCT05376800  NCT03964233 | MDM2 inhibitor | Advanced solid tumors; Glioblastoma; | No |
|  | Cbl-b | NX-1607 | 1 | NCT05107674 ^141^ | CBLB deletion induces IFNγ and suppresses TGFβ/SMAD signaling | Advanced malignancies | IFNγ helps CBLB-deficient CD8+ T cells resist Tregs |
|  | CRL4 | KPG-818 | 1 | NCT04283097 | modulator of the E3 ubiquitin ligase complex CRL4-CRBN, showing high affinity for the target CRBN | Hematological malignancies | No |

Abbreviations: ALL: acute lymphoblastic leukemia; AML: acute myeloid leukemia; CR: complete remission; DCR: disease control rate; HDM2: human double minute 2;  LSCs: leukemia stem cells; mPFS: median progression-free survival; MSS: microsatellite stable; MTD: maximum tolerated dose; NSCLC: non-small cell lung cancer; PK: pharmacokinetic; PR: partial response; SD: stable disease.

**Table S5.**

**Representative small molecule inhibitors and small molecules targeting protein degradation under clinical evaluation.**

**References**

1. Qiu, X.-B. & Goldberg, A. L. Nrdp1/FLRF is a ubiquitin ligase promoting ubiquitination and degradation of the epidermal growth factor receptor family member, ErbB3. *Proc Natl Acad Sci U S A* **99**, 14843–14848 (2002).

2. Liu, Y. *et al.* MARCH6 promotes Papillary Thyroid Cancer development by destabilizing DHX9. *Int J Biol Sci* **17**, 3401–3412 (2021).

3. Lee, Y. *et al.* ANKRD9 is associated with tumor suppression as a substrate receptor subunit of ubiquitin ligase. *Biochim Biophys Acta Mol Basis Dis* **1864**, 3145–3153 (2018).

4. Lee, M.-S. *et al.* PI3K/AKT activation induces PTEN ubiquitination and destabilization accelerating tumourigenesis. *Nat Commun* **11**, 6236 (2015).

5. Ruschmann, J. *et al.* Tyrosine phosphorylation of SHIP promotes its proteasomal degradation. *Exp Hematol* **38**, 392-402.e1 (2010).

6. Tan, M., Xu, J., Siddiqui, J., Feng, F. & Sun, Y. Depletion of SAG/RBX2 E3 ubiquitin ligase suppresses prostate tumorigenesis via inactivation of the PI3K/AKT/mTOR axis. *Mol Cancer* **15**, 81 (2016).

7. Joo, H. M. *et al.* Ret finger protein 2 enhances ionizing radiation-induced apoptosis via degradation of AKT and MDM2. *Eur J Cell Biol* **90**, 420–431 (2011).

8. Xiang, T. *et al.* Negative Regulation of AKT Activation by BRCA1. *Cancer Res* **68**, 10040–10044 (2008).

9. Su, C.-H. *et al.* Akt phosphorylation at Thr308 and Ser473 is required for CHIP-mediated ubiquitination of the kinase. *Cell Signal* **23**, 1824–1830 (2011).

10. Chan, C.-H. *et al.* The Skp2-SCF E3 ligase regulates Akt ubiquitination, glycolysis, herceptin sensitivity, and tumorigenesis. *Cell* **149**, 1098–1111 (2012).

11. Li, W. *et al.* TRAF4 is a critical molecule for Akt activation in lung cancer. *Cancer Res* **73**, 6938–6950 (2013).

12. Zhang, J. *et al.* The F-box protein FBXL18 promotes glioma progression by promoting K63-linked ubiquitination of Akt. *FEBS Lett* **591**, 145–154 (2017).

13. Sarri, N. *et al.* Deubiquitinating enzymes USP4 and USP17 finetune the trafficking of PDGFRβ and affect PDGF-BB-induced STAT3 signalling. *Cell Mol Life Sci* **79**, 85 (2022).

14. Xia, X. *et al.* The deubiquitinating enzyme USP15 stabilizes ERα and promotes breast cancer progression. *Cell Death Dis* **12**, 329 (2021).

15. Zhang, H. *et al.* USP4 promotes the proliferation, migration, and invasion of esophageal squamous cell carcinoma by targeting TAK1. *Cell Death Dis* **14**, 730 (2023).

16. Xia, X. *et al.* Deubiquitination and stabilization of estrogen receptor α by ubiquitin-specific protease 7 promotes breast tumorigenesis. *Cancer Lett* **465**, 118–128 (2019).

17. Sacco, J. J. *et al.* The deubiquitylase Ataxin-3 restricts PTEN transcription in lung cancer cells. *Oncogene* **33**, 4265–4272 (2014).

18. Zhang, J. *et al.* Deubiquitylation and stabilization of PTEN by USP13. *Nat Cell Biol* **15**, 1486–1494 (2013).

19. Lu, C. *et al.* USP10 suppresses tumor progression by inhibiting mTOR activation in hepatocellular carcinoma. *Cancer Lett* **436**, 139–148 (2018).

20. Sun, J. *et al.* USP10 inhibits lung cancer cell growth and invasion by upregulating PTEN. *Mol Cell Biochem* **441**, 1–7 (2018).

21. Yuan, L. *et al.* Deubiquitylase OTUD3 regulates PTEN stability and suppresses tumorigenesis. *Nat Cell Biol* **17**, 1169–1181 (2015).

22. Li, X. *et al.* The deubiquitination enzyme USP46 functions as a tumor suppressor by controlling PHLPP-dependent attenuation of Akt signaling in colon cancer. *Oncogene* **32**, 471–478 (2013).

23. Zhiqiang, Z. *et al.* USP1 regulates AKT phosphorylation by modulating the stability of PHLPP1 in lung cancer cells. *J Cancer Res Clin Oncol* **138**, 1231–1238 (2012).

24. Li, W., Cui, K., Prochownik, E. V. & Li, Y. The deubiquitinase USP21 stabilizes MEK2 to promote tumor growth. *Cell Death Dis* **9**, 482 (2018).

25. Gangula, N. R. & Maddika, S. WD repeat protein WDR48 in complex with deubiquitinase USP12 suppresses Akt-dependent cell survival signaling by stabilizing PH domain leucine-rich repeat protein phosphatase 1 (PHLPP1). *J Biol Chem* **288**, 34545–34554 (2013).

26. Jin, J.-O. *et al.* Sequential ubiquitination of p53 by TRIM28, RLIM, and MDM2 in lung tumorigenesis. *Cell Death Differ* **28**, 1790–1803 (2021).

27. Wu, J. *et al.* Skp2 E3 ligase integrates ATM activation and homologous recombination repair by ubiquitinating NBS1. *Mol Cell* **46**, 351–361 (2012).

28. Lovly, C. M., Yan, L., Ryan, C. E., Takada, S. & Piwnica-Worms, H. Regulation of Chk2 ubiquitination and signaling through autophosphorylation of serine 379. *Mol Cell Biol* **28**, 5874–5885 (2008).

29. Yuan, J., Luo, K., Zhang, L., Cheville, J. C. & Lou, Z. USP10 regulates p53 localization and stability by deubiquitinating p53. *Cell* **140**, 384–396 (2010).

30. Zhou, Y.-A. *et al.* Helicobacter pylori activates DOPEY1 to promote p53 degradation through the USP7/TRIP12 axis in gastric tumorigenesis. *Oncogene* (2025) doi:10.1038/s41388-025-03303-5.

31. Benard, G. *et al.* IBRDC2, an IBR-type E3 ubiquitin ligase, is a regulatory factor for Bax and apoptosis activation. *EMBO J* **29**, 1458–1471 (2010).

32. Allen, I. C. *et al.* The NLRP3 inflammasome functions as a negative regulator of tumorigenesis during colitis-associated cancer. *J Exp Med* **207**, 1045–1056 (2010).

33. Song, H. *et al.* The E3 ubiquitin ligase TRIM31 attenuates NLRP3 inflammasome activation by promoting proteasomal degradation of NLRP3. *Nat Commun* **7**, 13727 (2016).

34. Li, X. *et al.* CUL3 (cullin 3)-mediated ubiquitination and degradation of BECN1 (beclin 1) inhibit autophagy and promote tumor progression. *Autophagy* **17**, 4323–4340 (2021).

35. Roedig, J. *et al.* USP22 controls necroptosis by regulating receptor-interacting protein kinase 3 ubiquitination. *EMBO Rep* **22**, e50163 (2021).

36. He, H. *et al.* USP24-GSDMB complex promotes bladder cancer proliferation via activation of the STAT3 pathway. *Int J Biol Sci* **17**, 2417–2429 (2021).

37. Ren, Y. *et al.* USP48 Stabilizes Gasdermin E to Promote Pyroptosis in Cancer. *Cancer Res* **83**, 1074–1093 (2023).

38. Li, D. *et al.* CST1 inhibits ferroptosis and promotes gastric cancer metastasis by regulating GPX4 protein stability via OTUB1. *Oncogene* **42**, 83–98 (2023).

39. Her, Y. R. & Chung, I. K. Ubiquitin Ligase RLIM Modulates Telomere Length Homeostasis through a Proteolysis of TRF1. *J Biol Chem* **284**, 8557–8566 (2009).

40. Lee, T. H., Perrem, K., Harper, J. W., Lu, K. P. & Zhou, X. Z. The F-box protein FBX4 targets PIN2/TRF1 for ubiquitin-mediated degradation and regulates telomere maintenance. *J Biol Chem* **281**, 759–768 (2006).

41. Wang, C. *et al.* The F-box protein β-TrCP promotes ubiquitination of TRF1 and regulates the ALT-associated PML bodies formation in U2OS cells. *Biochem Biophys Res Commun* **434**, 728–734 (2013).

42. Wang, L. *et al.* FBW7 Mediates Senescence and Pulmonary Fibrosis through Telomere Uncapping. *Cell Metab* **32**, 860-877.e9 (2020).

43. Fujita, K. *et al.* Positive feedback between p53 and TRF2 during telomere-damage signalling and cellular senescence. *Nat Cell Biol* **12**, 1205–1212 (2010).

44. Ma, B. *et al.* The SIAH2-NRF1 axis spatially regulates tumor microenvironment remodeling for tumor progression. *Nat Commun* **10**, 1034 (2019).

45. Rai, R. *et al.* The E3 ubiquitin ligase Rnf8 stabilizes Tpp1 to promote telomere end protection. *Nat Struct Mol Biol* **18**, 1400–1407 (2011).

46. Yang, F. *et al.* FBXW2 suppresses migration and invasion of lung cancer cells via promoting β-catenin ubiquitylation and degradation. *Nat Commun* **10**, 1382 (2019).

47. Xu, J. *et al.* The β-TrCP-FBXW2-SKP2 axis regulates lung cancer cell growth with FBXW2 acting as a tumour suppressor. *Nat Commun* **8**, 14002 (2017).

48. Zhou, T. *et al.* FBXW2 inhibits prostate cancer proliferation and metastasis via promoting EGFR ubiquitylation and degradation. *Cell Mol Life Sci* **79**, 268 (2022).

49. Liu, W. *et al.* RNF126-Mediated MRE11 Ubiquitination Activates the DNA Damage Response and Confers Resistance of Triple-Negative Breast Cancer to Radiotherapy. *Adv Sci (Weinh)* **10**, e2203884 (2023).

50. Chen, Y. *et al.* USP44 regulates irradiation-induced DNA double-strand break repair and suppresses tumorigenesis in nasopharyngeal carcinoma. *Nat Commun* **13**, 501 (2022).

51. Liu, X. *et al.* USP7 reduces the level of nuclear DICER, impairing DNA damage response and promoting cancer progression. *Mol Oncol* **18**, 170–189 (2024).

52. Liu, J. *et al.* De-ubiquitination of SAMHD1 by USP7 promotes DNA damage repair to overcome oncogenic stress and affect chemotherapy sensitivity. *Oncogene* **42**, 1843–1856 (2023).

53. Traweek, R. S. *et al.* Targeting the MDM2-p53 pathway in dedifferentiated liposarcoma. *Front Oncol* **12**, 1006959 (2022).

54. Deng, Y. *et al.* RAD6 Positively Affects Tumorigenesis of Esophageal Squamous Cell Carcinoma by Regulating Histone Ubiquitination of CCNB1. *Biol Proced Online* **24**, 4 (2022).

55. Zhang, N. *et al.* E3 ubiquitin ligase RNF180 prevents excessive PCDH10 methylation to suppress the proliferation and metastasis of gastric cancer cells by promoting ubiquitination of DNMT1. *Clin Epigenetics* **15**, 77 (2023).

56. Sun, W. *et al.* DNMT3A-mediated silence in ADAMTS9 expression is restored by RNF180 to inhibit viability and motility in gastric cancer cells. *Cell Death Dis* **12**, 428 (2021).

57. Yadav, P. *et al.* M6A RNA Methylation Regulates Histone Ubiquitination to Support Cancer Growth and Progression. *Cancer Res* **82**, 1872–1889 (2022).

58. Huang, L.-Y. *et al.* SCFFBW7-mediated degradation of Brg1 suppresses gastric cancer metastasis. *Nat Commun* **9**, 3569 (2018).

59. Zhang, S. *et al.* mTORC1 Promotes ARID1A Degradation and Oncogenic Chromatin Remodeling in Hepatocellular Carcinoma. *Cancer Res* **81**, 5652–5665 (2021).

60. Seo, H.-R. *et al.* CHIP and BAP1 Act in Concert to Regulate INO80 Ubiquitination and Stability for DNA Replication. *Mol Cells* **44**, 101–115 (2021).

61. Cheng, X., Zhang, B., Guo, F., Wu, H. & Jin, X. Deubiquitination of FBP1 by USP7 blocks FBP1-DNMT1 interaction and decreases the sensitivity of pancreatic cancer cells to PARP inhibitors. *Mol Oncol* **16**, 1591–1607 (2022).

62. Fu, X. *et al.* OTUD6A promotes prostate tumorigenesis via deubiquitinating Brg1 and AR. *Commun Biol* **5**, 182 (2022).

63. Cao, C. *et al.* BICP0 Negatively Regulates TRAF6-Mediated NF-κB and Interferon Activation by Promoting K48-Linked Polyubiquitination of TRAF6. *Front Microbiol* **10**, 3040 (2019).

64. Zhang, L. *et al.* TRIM22 inhibits endometrial cancer progression through the NOD2/NF‑κB signaling pathway and confers a favorable prognosis. *Int J Oncol* **56**, 1225–1239 (2020).

65. Asano, N. *et al.* Cdx2 Expression and Intestinal Metaplasia Induced by H. pylori Infection of Gastric Cells Is Regulated by NOD1-Mediated Innate Immune Responses. *Cancer Res* **76**, 1135–1145 (2016).

66. Jo, T. *et al.* LUBAC accelerates B-cell lymphomagenesis by conferring resistance to genotoxic stress on B cells. *Blood* **136**, 684–697 (2020).

67. Song, K. *et al.* Epsins 1 and 2 promote NEMO linear ubiquitination via LUBAC to drive breast cancer development. *J Clin Invest* **131**, e129374 (2021).

68. Damgaard, R. B. *et al.* OTULIN protects the liver against cell death, inflammation, fibrosis, and cancer. *Cell Death Differ* **27**, 1457–1474 (2020).

69. Tian, S. *et al.* Network Medicine-Based Strategy Identifies Maprotiline as a Repurposable Drug by Inhibiting PD-L1 Expression via Targeting SPOP in Cancer. *Adv Sci (Weinh)* **12**, e2410285 (2025).

70. Zhang, J. *et al.* Cyclin D-CDK4 kinase destabilizes PD-L1 via cullin 3-SPOP to control cancer immune surveillance. *Nature* **553**, 91–95 (2018).

71. Liu, W., Ren, D., Xiong, W., Jin, X. & Zhu, L. A novel FBW7/NFAT1 axis regulates cancer immunity in sunitinib-resistant renal cancer by inducing PD-L1 expression. *J Exp Clin Cancer Res* **41**, 38 (2022).

72. Meng, X. *et al.* FBXO38 mediates PD-1 ubiquitination and regulates anti-tumour immunity of T cells. *Nature* **564**, 130–135 (2018).

73. De, S., Holvey-Bates, E. G., Mahen, K., Willard, B. & Stark, G. R. The ubiquitin E3 ligase FBXO22 degrades PD-L1 and sensitizes cancer cells to DNA damage. *Proc Natl Acad Sci U S A* **118**, e2112674118 (2021).

74. Jing, W. *et al.* FGFR3 Destabilizes PD-L1 via NEDD4 to Control T-cell-Mediated Bladder Cancer Immune Surveillance. *Cancer Res* **82**, 114–129 (2022).

75. Karwacz, K. *et al.* PD-L1 co-stimulation contributes to ligand-induced T cell receptor down-modulation on CD8+ T cells. *EMBO Mol Med* **3**, 581–592 (2011).

76. Liu, Y. *et al.* HERC2 promotes inflammation-driven cancer stemness and immune evasion in hepatocellular carcinoma by activating STAT3 pathway. *J Exp Clin Cancer Res* **42**, 38 (2023).

77. Yang, H. *et al.* RNF31 represses cell progression and immune evasion via YAP/PD-L1 suppression in triple negative breast Cancer. *J Exp Clin Cancer Res* **41**, 364 (2022).

78. Pitarresi, J. R. *et al.* Disruption of stromal hedgehog signaling initiates RNF5-mediated proteasomal degradation of PTEN and accelerates pancreatic tumor growth. *Life Sci Alliance* **1**, e201800190 (2018).

79. Lee, J.-H. *et al.* A20 promotes metastasis of aggressive basal-like breast cancers through multi-monoubiquitylation of Snail1. *Nat Cell Biol* **19**, 1260–1273 (2017).

80. Yu, J. *et al.* The OX40-TRAF6 axis promotes CTLA-4 degradation to augment antitumor CD8+ T-cell immunity. *Cell Mol Immunol* **20**, 1445–1456 (2023).

81. Wang, Z. *et al.* Abrogation of USP7 is an alternative strategy to downregulate PD-L1 and sensitize gastric cancer cells to T cells killing. *Acta Pharm Sin B* **11**, 694–707 (2021).

82. Huang, X. *et al.* USP22 Deubiquitinates CD274 to Suppress Anticancer Immunity. *Cancer Immunol Res* **7**, 1580–1590 (2019).

83. Wang, Y. *et al.* The deubiquitinase USP22 regulates PD-L1 degradation in human cancer cells. *Cell Commun Signal* **18**, 112 (2020).

84. Lim, S.-O. *et al.* Deubiquitination and Stabilization of PD-L1 by CSN5. *Cancer Cell* **30**, 925–939 (2016).

85. Yang, H. *et al.* Targeting ubiquitin-specific protease 8 sensitizes anti-programmed death-ligand 1 immunotherapy of pancreatic cancer. *Cell Death Differ* **30**, 560–575 (2023).

86. Shi, D. *et al.* USP14 promotes tryptophan metabolism and immune suppression by stabilizing IDO1 in colorectal cancer. *Nat Commun* **13**, 5644 (2022).

87. Yang, Z. *et al.* USP12 downregulation orchestrates a protumourigenic microenvironment and enhances lung tumour resistance to PD-1 blockade. *Nat Commun* **12**, 4852 (2021).

88. Jingjing, W. *et al.* Deubiquitination and stabilization of programmed cell death ligand 1 by ubiquitin-specific peptidase 9, X-linked in oral squamous cell carcinoma. *Cancer Med* **7**, 4004–4011 (2018).

89. Wu, X. *et al.* Ubiquitin-specific protease 3 promotes cell migration and invasion by interacting with and deubiquitinating SUZ12 in gastric cancer. *J Exp Clin Cancer Res* **38**, 277 (2019).

90. Eichhorn, P. J. A. *et al.* USP15 stabilizes TGF-β receptor I and promotes oncogenesis through the activation of TGF-β signaling in glioblastoma. *Nat Med* **18**, 429–435 (2012).

91. Zhang, L. *et al.* USP4 is regulated by AKT phosphorylation and directly deubiquitylates TGF-β type I receptor. *Nat Cell Biol* **14**, 717–726 (2012).

92. Lambies, G. *et al.* TGFβ-Activated USP27X Deubiquitinase Regulates Cell Migration and Chemoresistance via Stabilization of Snail1. *Cancer Res* **79**, 33–46 (2019).

93. Wu, Y. *et al.* Dub3 inhibition suppresses breast cancer invasion and metastasis by promoting Snail1 degradation. *Nat Commun* **8**, 14228 (2017).

94. Ryu, T. Y. *et al.* Human gut-microbiome-derived propionate coordinates proteasomal degradation via HECTD2 upregulation to target EHMT2 in colorectal cancer. *ISME J* **16**, 1205–1221 (2022).

95. Zhao, X. *et al.* Trigred motif 36 regulates neuroendocrine differentiation of prostate cancer via HK2 ubiquitination and GPx4 deficiency. *Cancer Sci* **114**, 2445–2459 (2023).

96. Wang, Z., Wang, M.-M., Geng, Y., Ye, C.-Y. & Zang, Y.-S. Membrane-associated RING-CH protein (MARCH8) is a novel glycolysis repressor targeted by miR-32 in colorectal cancer. *J Transl Med* **20**, 402 (2022).

97. Li, M. *et al.* Tanshinone IIA inhibits oral squamous cell carcinoma via reducing Akt-c-Myc signaling-mediated aerobic glycolysis. *Cell Death Dis* **11**, 381 (2020).

98. Zhao, G. *et al.* DDX39B drives colorectal cancer progression by promoting the stability and nuclear translocation of PKM2. *Signal Transduct Target Ther* **7**, 275 (2022).

99. Han, J. *et al.* Transcriptional dysregulation of TRIM29 promotes colorectal cancer carcinogenesis via pyruvate kinase-mediated glucose metabolism. *Aging* **13**, 5034–5054 (2021).

100. Wu, H., Guo, X., Jiao, Y., Wu, Z. & Lv, Q. TRIM35 ubiquitination regulates the expression of PKM2 tetramer and dimer and affects the malignant behaviour of breast cancer by regulating the Warburg effect. *Int J Oncol* **61**, 144 (2022).

101. Ito, H., Nakamae, I., Kato, J.-Y. & Yoneda-Kato, N. Stabilization of fatty acid synthesis enzyme acetyl-CoA carboxylase 1 suppresses acute myeloid leukemia development. *J Clin Invest* **131**, e141529 (2021).

102. Wei, W. *et al.* FBXW7β loss-of-function enhances FASN-mediated lipogenesis and promotes colorectal cancer growth. *Signal Transduct Target Ther* **8**, 187 (2023).

103. Wang, T. *et al.* Deacetylation of Glutaminase by HDAC4 contributes to Lung Cancer Tumorigenesis. *Int J Biol Sci* **18**, 4452–4465 (2022).

104. Zheng, X., Shao, J., Qian, J. & Liu, S. circRPS19 affects HK2‑mediated aerobic glycolysis and cell viability via the miR‑125a‑5p/USP7 pathway in gastric cancer. *Int J Oncol* **63**, 98 (2023).

105. Han, X. *et al.* Deubiquitination of MYC by OTUB1 contributes to HK2 mediated glycolysis and breast tumorigenesis. *Cell Death Differ* **29**, 1864–1873 (2022).

106. He, Y. *et al.* USP7 promotes non-small-cell lung cancer cell glycolysis and survival by stabilizing and activating c-Abl. *Clin Transl Med* **13**, e1509 (2023).

107. Maddalena, F. *et al.* TRAP1 enhances Warburg metabolism through modulation of PFK1 expression/activity and favors resistance to EGFR inhibitors in human colorectal carcinomas. *Mol Oncol* **14**, 3030–3047 (2020).

108. Yu, S., Zang, W., Qiu, Y., Liao, L. & Zheng, X. Deubiquitinase OTUB2 exacerbates the progression of colorectal cancer by promoting PKM2 activity and glycolysis. *Oncogene* **41**, 46–56 (2022).

109. Sun, T., Liu, Z., Bi, F. & Yang, Q. Deubiquitinase PSMD14 promotes ovarian cancer progression by decreasing enzymatic activity of PKM2. *Mol Oncol* **15**, 3639–3658 (2021).

110. Lv, T. *et al.* USP35 promotes hepatocellular carcinoma progression by protecting PKM2 from ubiquitination‑mediated degradation. *Int J Oncol* **63**, 113 (2023).

111. Nguyen, T. V. USP15 antagonizes CRL4CRBN-mediated ubiquitylation of glutamine synthetase and neosubstrates. *Proc Natl Acad Sci U S A* **118**, e2111391118 (2021).

112. Ning, Z. *et al.* USP22 regulates lipidome accumulation by stabilizing PPARγ in hepatocellular carcinoma. *Nat Commun* **13**, 2187 (2022).

113. Nakamura, A. *et al.* The SUMOylation inhibitor subasumstat potentiates rituximab activity by IFN1-dependent macrophage and NK cell stimulation. *Blood* **139**, 2770–2781 (2022).

114. Faessel, H. M. *et al.* Population pharmacokinetics of pevonedistat alone or in combination with standard of care in patients with solid tumours or haematological malignancies. *Br J Clin Pharmacol* **85**, 2568–2579 (2019).

115. Handa, H. *et al.* Pevonedistat in East Asian patients with acute myeloid leukemia or myelodysplastic syndromes: a phase 1/1b study to evaluate safety, pharmacokinetics and activity as a single agent and in combination with azacitidine. *J Hematol Oncol* **15**, 56 (2022).

116. Zhou, X. *et al.* Phase I study assessing the mass balance, pharmacokinetics, and excretion of [14C]-pevonedistat, a NEDD8-activating enzyme inhibitor in patients with advanced solid tumors. *Invest New Drugs* **39**, 488–498 (2021).

117. Swords, R. T. *et al.* Pevonedistat, a first-in-class NEDD8-activating enzyme inhibitor, combined with azacitidine in patients with AML. *Blood* **131**, 1415–1424 (2018).

118. Swords, R. T. *et al.* Expanded safety analysis of pevonedistat, a first-in-class NEDD8-activating enzyme inhibitor, in patients with acute myeloid leukemia and myelodysplastic syndromes. *Blood Cancer J* **7**, e520 (2017).

119. Faessel, H. *et al.* Effect of CYP3A inhibitors on the pharmacokinetics of pevonedistat in patients with advanced solid tumours. *Br J Clin Pharmacol* **85**, 1464–1473 (2019).

120. Zhou, X. *et al.* Phase 1 study to evaluate the effects of rifampin on pharmacokinetics of pevonedistat, a NEDD8-activating enzyme inhibitor in patients with advanced solid tumors. *Invest New Drugs* **40**, 1042–1050 (2022).

121. Zhou, X. *et al.* Effect of Pevonedistat, an Investigational NEDD8-Activating Enzyme Inhibitor, on the QTc Interval in Patients With Advanced Solid Tumors. *Clin Pharmacol Drug Dev* **12**, 257–266 (2023).

122. Sarantopoulos, J. *et al.* Phase I Study of the Investigational NEDD8-Activating Enzyme Inhibitor Pevonedistat (TAK-924/MLN4924) in Patients with Advanced Solid Tumors. *Clin Cancer Res* **22**, 847–857 (2016).

123. Bhatia, S. *et al.* A phase I study of the investigational NEDD8-activating enzyme inhibitor pevonedistat (TAK-924/MLN4924) in patients with metastatic melanoma. *Invest New Drugs* **34**, 439–449 (2016).

124. Shah, J. J. *et al.* Phase I Study of the Novel Investigational NEDD8-Activating Enzyme Inhibitor Pevonedistat (MLN4924) in Patients with Relapsed/Refractory Multiple Myeloma or Lymphoma. *Clin Cancer Res* **22**, 34–43 (2016).

125. Gounder, M. M. *et al.* A First-in-Human Phase I Study of Milademetan, an MDM2 Inhibitor, in Patients With Advanced Liposarcoma, Solid Tumors, or Lymphomas. *J Clin Oncol* **41**, 1714–1724 (2023).

126. Abdul Razak, A. R. *et al.* Co-Targeting of MDM2 and CDK4/6 with Siremadlin and Ribociclib for the Treatment of Patients with Well-Differentiated or Dedifferentiated Liposarcoma: Results from a Proof-of-Concept, Phase Ib Study. *Clin Cancer Res* **28**, 1087–1097 (2022).

127. Erba, H. P. *et al.* Phase 1b study of the MDM2 inhibitor AMG 232 with or without trametinib in relapsed/refractory acute myeloid leukemia. *Blood Adv* **3**, 1939–1949 (2019).

128. Patnaik, A. *et al.* Clinical pharmacology characterization of RG7112, an MDM2 antagonist, in patients with advanced solid tumors. *Cancer Chemother Pharmacol* **76**, 587–595 (2015).

129. de Jonge, M. *et al.* A phase I study of SAR405838, a novel human double minute 2 (HDM2) antagonist, in patients with solid tumours. *Eur J Cancer* **76**, 144–151 (2017).

130. de Weger, V. A. *et al.* A phase I study of the HDM2 antagonist SAR405838 combined with the MEK inhibitor pimasertib in patients with advanced solid tumours. *Br J Cancer* **120**, 286–293 (2019).

131. Yee, K. *et al.* Murine double minute 2 inhibition alone or with cytarabine in acute myeloid leukemia: Results from an idasanutlin phase 1/1b study⋆. *Leuk Res* **100**, 106489 (2021).

132. Italiano, A. *et al.* Phase I study of daily and weekly regimens of the orally administered MDM2 antagonist idasanutlin in patients with advanced tumors. *Invest New Drugs* **39**, 1587–1597 (2021).

133. Bauer, S. *et al.* Pharmacokinetic-pharmacodynamic guided optimisation of dose and schedule of CGM097, an HDM2 inhibitor, in preclinical and clinical studies. *Br J Cancer* **125**, 687–698 (2021).

134. Infante, J. R. *et al.* Phase I dose-escalation study of LCL161, an oral inhibitor of apoptosis proteins inhibitor, in patients with advanced solid tumors. *J Clin Oncol* **32**, 3103–3110 (2014).

135. Crawford, N. *et al.* Clinical Positioning of the IAP Antagonist Tolinapant (ASTX660) in Colorectal Cancer. *Mol Cancer Ther* **20**, 1627–1639 (2021).

136. Sun, X.-S. *et al.* Debio 1143 and high-dose cisplatin chemoradiotherapy in high-risk locoregionally advanced squamous cell carcinoma of the head and neck: a double-blind, multicentre, randomised, phase 2 study. *Lancet Oncol* **21**, 1173–1187 (2020).

137. Hurwitz, H. I. *et al.* Safety, pharmacokinetics, and pharmacodynamic properties of oral DEBIO1143 (AT-406) in patients with advanced cancer: results of a first-in-man study. *Cancer Chemother Pharmacol* **75**, 851–859 (2015).

138. Surka, C. *et al.* CC-90009, a novel cereblon E3 ligase modulator, targets acute myeloid leukemia blasts and leukemia stem cells. *Blood* **137**, 661–677 (2021).

139. Abdul Razak, A. R. *et al.* A phase 1 study of the MDM2 antagonist RO6839921, a pegylated prodrug of idasanutlin, in patients with advanced solid tumors. *Invest New Drugs* **38**, 1156–1165 (2020).

140. Uy, G. L. *et al.* Phase 1 study of the MDM2 antagonist RO6839921 in patients with acute myeloid leukemia. *Invest New Drugs* **38**, 1430–1441 (2020).

141. Wolf, D. & Baier, G. IFNγ Helps CBLB-Deficient CD8+ T Cells to Put Up Resistance to Tregs. *Cancer Immunol Res* **10**, 370 (2022).
